# Chromosome-level assembly and annotation of the blue catfish *Ictalurus furcatus*, an aquaculture species for hybrid catfish reproduction, epigenetics, and heterosis studies

**DOI:** 10.1093/gigascience/giac070

**Published:** 2022-07-09

**Authors:** Haolong Wang, Baofeng Su, Ian A E Butts, Rex A Dunham, Xu Wang

**Affiliations:** Department of Pathobiology, College of Veterinary Medicine, Auburn University, Auburn, AL 36849, USA; Alabama Agricultural Experiment Station, Auburn, AL 36849, USA; Alabama Agricultural Experiment Station, Auburn, AL 36849, USA; School of Fisheries, Aquaculture and Aquatic Sciences, Auburn University, Auburn, AL 36849, USA; Alabama Agricultural Experiment Station, Auburn, AL 36849, USA; School of Fisheries, Aquaculture and Aquatic Sciences, Auburn University, Auburn, AL 36849, USA; Alabama Agricultural Experiment Station, Auburn, AL 36849, USA; School of Fisheries, Aquaculture and Aquatic Sciences, Auburn University, Auburn, AL 36849, USA; Department of Pathobiology, College of Veterinary Medicine, Auburn University, Auburn, AL 36849, USA; Alabama Agricultural Experiment Station, Auburn, AL 36849, USA; HudsonAlpha Institute for Biotechnology, Huntsville, AL 35806, USA

**Keywords:** blue catfish, chromosomal assembly, channel catfish, heterosis, epigenetics, linked-reads technology, PacBio sequencing, D&B, Rio Grande

## Abstract

**Background:**

The blue catfish is of great value in aquaculture and recreational fisheries. The F_1_ hybrids of female channel catfish (*Ictalurus punctatus*) × male blue catfish (*Ictalurusfurcatus*) have been the primary driver of US catfish production in recent years because of superior growth, survival, and carcass yield. The channel–blue hybrid also provides an excellent model to investigate molecular mechanisms of environment-dependent heterosis. However, transcriptome and methylome studies suffered from low alignment rates to the channel catfish genome due to divergence, and the genome resources for blue catfish are not publicly available.

**Results:**

The blue catfish genome assembly is 841.86 Mbp in length with excellent continuity (8.6 Mbp contig N50, 28.2 Mbp scaffold N50) and completeness (98.6% Eukaryota and 97.0% Actinopterygii BUSCO). A total of 30,971 protein-coding genes were predicted, of which 21,781 were supported by RNA sequencing evidence. Phylogenomic analyses revealed that it diverged from channel catfish approximately 9 million years ago with 15.7 million fixed nucleotide differences. The within-species single-nucleotide polymorphism (SNP) density is 0.32% between the most aquaculturally important blue catfish strains (D&B and Rio Grande). Gene family analysis discovered significant expansion of immune-related families in the blue catfish lineage, which may contribute to disease resistance in blue catfish.

**Conclusions:**

We reported the first high-quality, chromosome-level assembly of the blue catfish genome, which provides the necessary genomic tool kit for transcriptome and methylome analysis, SNP discovery and marker-assisted selection, gene editing and genome engineering, and reproductive enhancement of the blue catfish and hybrid catfish.

## Introduction

Catfish is the largest segment of US aquaculture [1], and catfish farming in Mississippi, Alabama, Arkansas, and Texas accounts for 70% of total US freshwater aquaculture production. Blue catfish, *Ictalurus furcatus* (NCBI:txid66913; Fishbase ID: 3019), is an important aquaculture species in the United States, which is native to the Mississippi River basin and along the Atlantic and Gulf coast slopes [2]. The hybrid of female channel catfish (C) and male blue catfish (B), *Ictaluruspunctatus* ♀ × *I. furcatus* ♂ (C × B), constitutes more than 50% of the total harvest [3]. Additionally, blue catfish is the largest catfish species in North America and has special value for the recreational fishery due to the demand for trophy catfish for many anglers [4, 5].

US catfish production peaked in 2003 at around 300 million kg. However, it has been declining since then due to increased feed and energy cost, low catfish market price, and competition from imports, notably Asian catfish [6, 7]. Interspecific hybridization is an efficient way to recover catfish industry prosperity by producing greater genetic enhancement. The F_1_ hybrid (C × B) is superior in many production traits, including faster growth rate [8–10], improved feed conversion efficiency [9, 11], more carcass yield [12], better low oxygen tolerance [13], disease resistance [14], and enhanced harvestability [15]. Collectively, these heterosis characteristics enable a commercial production rate of 13,000 kg/ha, which doubles the yield of traditional channel catfish farming [11, 16, 17]. In this context, paternal genetic contributions from blue catfish are essential for improving industry-relevant traits [18], and yet the blue catfish genome resources are not publicly available. A high-quality genome assembly of the blue catfish genome will provide the essential toolkit for the following research areas to enhance catfish breeding and advance the scientific knowledge of heterosis in fish.

In channel–blue catfish hybrids, only C × B hybrids demonstrated heterobeltiosis characteristics [15], and the superior phenotypes were only observed in pond culture but not in smaller culturing units 1 m^3^ or less [19]. The asymmetric and environment-dependent heterosis remains a mystery in the genetics and evolutionary biology field [20]. Transgressive genes, which are defined as genes with higher or lower expression than both parents in F_1_ hybrids [21], may contribute to the superior performance in heterosis, or misregulation of gene expression resulting in hybrid incompatibility [22]. To understand the molecular mechanism of heterosis, we performed RNA sequencing (RNA-seq) in blue catfish, channel catfish, and their F_1_ hybrids to determine how gene regulation in hybrid catfish was altered. However, only ∼60% of the blue catfish reads can be aligned to the channel catfish genome [23] due to sequence divergence, which greatly diminished the ability to investigate the gene expression differences genome-wide. Thus, a high-quality blue catfish genome assembly is necessary to improve the RNA-seq mapping rate.

Using channel catfish × blue catfish hybrid crosses and backcrosses, previous research identified major genetic loci responsible for the resistance of 3 economically important catfish diseases [24–27]. For certain bacterial pathogens, the blue catfish allele was the most resistant. Blue catfish were almost completely resistant to *Edwardsiella ictaluri*, the pathogen for enteric septicemia of catfish [28, 29], whereas a 26% mortality in C × B hybrids [29] and up to 72.3% mortality in channel catfish were reported [28]. Blue catfish were also more resistant to the *Aeromonas* disease than C × B hybrids, whose mortality (32%) [30, 31] was much lower than the channel catfish (90% mortality reported in [32] and 78% in [33]) under the infection of pathogenic *Aeromonas hydrophila*. Understanding the blue catfish genome will facilitate the selection of the disease-resistant alleles from blue catfish for superior hybrid catfish breeds. As a cost-effective approach, marker-assisted selection (MAS) has been applied to select superior breeders for traits of interest, which relies on the selection of the best representative single-nucleotide polymorphisms (SNPs) from genome-wide association study (GWAS) peaks. Public genome assembly and annotation are already available for channel catfish [23, 34], and the catfish SNP genotyping arrays were designed primarily based on the channel catfish sequences [35, 36]. A high-quality blue catfish genome is required for the selection of ideal SNP marker sets from GWAS/Quantitative trait locus (QTL) mapping results to ensure equal polymerase chain reaction (PCR) amplification efficiency for both the channel catfish and blue catfish allele, as well as avoid SNPs located in the repeat region or paralogous sequences in either species.

In addition to transcriptomic and genomic analyses, a blue catfish reference genome will also enable epigenomic investigations, which is critical for studying the heterosis and reproductive biology of the hybrid catfish. To explain the phenotypic differences between B × C and C × B hybrids, an epigenetic component must be considered because the F_1_ hybrids have identical nuclear genome configurations (29 chromosomes from the blue catfish and 29 from the channel catfish) [37]. As a key epigenetic modification [38], DNA methylation may be differentially marked in the male germline of channel catfish versus blue catfish, affecting the global gene expression profile. The C × B hybrid catfish fry were produced using artificial reproduction techniques by mixing eggs and sperm *in vitro* [39]. Unlike other fish species, in which sperm could be easily obtained by stripping, blue catfish sperm is collected by the removal and maceration of testes, which is a lethal procedure [15, 40]. Since blue catfish males become sexually mature at 4–7 years of age [2], the paternal side (blue catfish) is the bottleneck in hybrid catfish embryo production. Cryopreservation of gametes is a solution to overcome paternal limitations during the spawning season [41], but substantial variations in hatch rate were reported for cryopreserved blue catfish sperm samples, ranging from 0% to 82% [42–46]. Thus, a reliable method for assessing sperm quality is in urgent need within the US catfish industry, and DNA methylation is the most promising biomarker. Sperm methylation has been linked to fertility in fish [47], and it has been reported that cryopreservation can affect the DNA methylation of sperm [48]. To determine whether different storage strategies induce epigenetic lesions, DNA methylome studies have been performed in blue catfish sperm, but the proportion of mapped reads to the channel catfish reference genome is relatively low, resulting in insufficient coverage for DNA methylome analysis. Thus, a high-quality, high-continuity blue catfish genome is required for epigenetic studies to enhance embryo production for hybrid catfish and also as a genetic resource for producing better genetic types of hybrid catfish.

To fill the gap in the catfish genomic toolkit, in this study, we reported the first genome assembly of blue catfish (*I. furcatus*) using PacBio long-read sequencing. This high-quality, high-continuity assembly will allow researchers to better investigate the genomic underpinnings of production phenotypes. The annotation of the blue catfish genome makes it possible to conduct comprehensive functional genomics studies. The blue catfish genome resource will provide a solid molecular basis for investigating the mechanism of heterosis in the C × B hybrids, as well as improving the genetic potential for commercial production by genetic enhancement programs.

## Materials and Methods

### Fish and blood collection

Four healthy adult blue catfish (body weight: 2.8–3.8 kg) were obtained from the brood stock ponds at the Auburn University Fish Genetics Research Unit (Auburn, AL, USA), including 2 blue catfish (1 female and 1 male) from the D&B strain and 2 blue catfish (1 female and 1 male) from Rio Grande strain. All the fish were anesthetized using 100 mg/L buffered MS-222 (tricaine methanesulfonate; Syndel Inc., Ferndale, WA, USA), and blood samples were collected using a syringe from the caudal vasculature and immediately put into the lithium heparin–containing blood collection tubes (The Becton, Dickinson and Company, Franklin Lakes, NJ, USA). After blood collection, the 4 fish were temporarily reared in an indoor tank with dissolved oxygen level >8 mg/L, water temperature 21–22.5°C, and pH 6.8–7.0, for them to recover for a few days before releasing back into the research pond. All experimental animal protocols, including animal care and tissue sample collections, were approved by the Auburn University Institutional Animal Care and Use Committee (IACUC) under PRN# 2019–3520.

### Genomic DNA extraction and quality control

High molecular weight (HMW) genomic DNA (gDNA) was extracted from 1 female D&B, 1 male D&B, 1 female Rio Grande, and 1 male Rio Grande blue catfish blood sample (Supplementary Table S1) using the Monarch^®^ HMW DNA Extraction Kit for Cells & Blood kit (New England BioLabs, Ipswich, MA, USA) following the manufacturer's protocol. A total of 20 μL blood sample was used as input for each extraction. The concentration of gDNA was determined by a Qubit 3.0 Fluorometer instrument (Thermo Fisher Scientific, Waltham, MA, USA). The gDNA integrity and size distribution were checked by an Agilent TapeStation 4200 (Agilent Technologies, Santa Clara, CA, USA).

### PacBio CCS HiFi library preparation and sequencing

PacBio CCS (circular consensus sequencing) library was constructed on 10 μg female D&B blue catfish HMW gDNA sheared into 20-kb fragments, using the SMRTbell Template Prep Kit v2 following the CCS HiFi library protocols (Pacific Biosciences, Menlo Park, CA, USA). The PacBio library was prepared and sequenced on a PacBio Sequel II System  (PacBio Sequel II System, RRID:SCR_017990) at the HudsonAlpha Genome Sequencing Center (HudsonAlpha Institute for Biotechnology, Huntsville, AL, USA).

### The 10× Genomics linked-read library preparation and Illumina short-read sequencing

Four 10× Genomics linked-read libraries (female and male of D&B and Rio Grande strains; Supplementary Table S1) were constructed on a 10× Genomics Chromium Controller (10× Genomics, Inc., San Francisco, CA, USA) using 1.1 ng HMW gDNA input with the Chromium Genome Reagent Kit v2 and Chromium Genome Library & Gel Bead Kit v2 (10× Genomics, Inc.). Individual libraries were barcoded using the Chromium i7 Multiplex Kit (10× Genomics, Inc.). Final library quality and size distribution were determined by Agilent TapeStation 4200 (Agilent Technologies), and the concentrations were measured with Qubit 3.0 Fluorometer (Thermo Fisher Scientific). The libraries were sequenced using a 2 × 150 bp paired-end format on an Illumina NovaSeq 6000 sequencing  (Illumina NovaSeq 6000 Sequencing System, RRID:SCR_016387) at Novogene (Novogene Corporation Inc., Sacramento, CA, USA).

### Genome size estimation

Illumina sequencing reads were used to estimate the genome size of *I. furcatus*. Adapter sequences and low-quality bases in the raw reads were trimmed by Trimmomatic version 0.36 (Trimmomatic, RRID:SCR_011848) [49], with the parameters “ILLUMINACLIP:adapter:2:30:10 LEADING:3 TRAILING:3 SLIDINGWINDOW:4:15 MINLEN:75.” The remaining high-quality reads in the D&B female DNA resequencing data were used for multiple *k*-mer counting using Jellyfish version 2.3.0  (Jellyfish, RRID:SCR_005491) [50] with parameters count “-m 25 -s 180 G -t 48.” The genome size, heterozygosity, and repeat level were estimated using GenomeScope  (GenomeScope, RRID:SCR_017014) [51] based on the *k*-mer frequency distributions.

### Genomic contig assembly

After removing the PacBio sequencing adapters and primers, the CCS HiFi reads were assembled to blue catfish contigs using hifiasm version 0.13 (Hifiasm, RRID:SCR_021069) [52] with default parameters. *De novo* assemblies of the 10× Genomics linked reads were performed using Supernova version 2.1.1  (Supernova assembler, RRID:SCR_016756) with default parameters [53]. The quickmerge software (version 0.3.0) [54] was used to combine the long-read and linked-read assemblies, but the outcome was the same as the PacBio contigs. Potential microbial contaminations were examined by a pipeline described previously [55], and none were identified. The contig assembly statistics were assessed using the stats.sh function in the BBMap package  (BBmap, RRID:SCR_016965) [56].

### Chromosome assembly and polishing

Based on previous cytogenetic studies, blue catfish have the same number of chromosomes as the channel catfish (2 N = 58), and the blue and channel catfish chromosomes cannot be distinguished in the karyotyping results [57]. To assemble the PacBio contigs into chromosomes, blue–channel catfish linkage information was utilized from previous linkage maps constructed using channel catfish × blue catfish crosses [58–62]. Specifically, PCR primer sequences for linkage marker from Ninwichian et al. [61] were identified in the blue catfish contigs using the UCSC In-Silico PCR tool [63] (Supplementary Data S1). The contigs were ordered based on the linkage map positions, and adjacent contigs were separated by 50,000 Ns. The draft genome assembly was polished using Illumina short reads (Supplementary Table S1) for indel and error correction to generate a final high-quality assembly by Pilon (version 1.24; parameter settings: fix = all) (Pilon, RRID:SCR_014731) [64]. Potential bacterial contaminations were checked using a pipeline described in our previous research [65], and no bacterial contamination was found at the chromosome assembly level either.

### Genome completeness and quality assessment

The final genome assembly statistics (Table 1) were determined by the stats.sh function in BBMap [56]. Genome completeness of blue catfish assembly was evaluated by BUSCO version 5.3.2 (RRID:SCR_015008) [66] and compared with the closely related channel catfish (*I. punctatus*) reference genome [23, 67] and tra catfish (*Pangasianodon hypophthalmus*, also known as striped catfish) genome assembled recently [68–70]. Orthologs in eukaryota_odb10 and actinopterygii_odb10 were used to compute the BUSCO scores. To determine the completeness at the chromosome termini, telomeric repeat motifs (TRMs) were identified and quantified from the Illumina sequencing data using the TRIP pipeline [71], and the 6-bp vertebrate-type TRM TTAGGG was identified.

**Table 1:** Summary statistics of the blue catfish (*Ictalurus furcatus*) genome assemblies

Genome assembly	Blue catfish (this assembly)	Channel catfish (ASM400665v3)	Tra catfish (GCA_016801045.1)
**Sequencing data and coverage**			
PacBio sequencing data	21.34 Gb PacBio Sequel II CCS HiFi reads	57.69 Gb PacBio Sequel CLS reads	63.07 Gb Nanopore
Illumina sequencing data	49.41 Gb NovaSeq reads	—	44.23 Gb HiSeq reads
Genome coverage	CCS: 24×; Illumina: 59×	CLS: 58×	Nanopore: 130×; Illumina: 59×
**Assembly statistics**			
Total scaffold length	841,864,377 bp	1,036,985,268 bp	742,562,378 bp
Total contig length	838,964,151 bp	1,018,280,134 bp	771,909,303 bp
Number of scaffolds	271	3,164	402
Scaffold N50	28.24 Mbp	26.68 Mbp	29.53 Mbp
Maximum scaffold length	38.50 Mbp	39.13 Mbp	45.06 Mbp
Number of contigs	563	6,999	808
Contig N50	8.59 Mbp	1.70 Mbp	3.48 Mbp
Maximum contig length	24.51 Mbp	24.09 Mbp	16.11 Mbp
**Completeness (eukaryota_odb10, *n* = 255)**			
BUSCO completeness	98.4%	95.7%	97.2%
Single-copy BUSCO	95.7%	83.9%	94.1%
Duplicated BUSCO	2.7%	11.8%	3.1%
Fragmented BUSCO	0.4%	1.6%	0.4%
Missing BUSCO	1.2%	2.7%	2.4%
**Completeness (actinopterygii_odb10, *n* = 3,640)**			
BUSCO completeness	97.0%	95.0%	96.1%
Single-copy BUSCO	95.4%	84.7%	94.9%
Duplicated BUSCO	1.6%	10.3%	1.2%
Fragmented BUSCO	0.6%	1.2%	0.8%
Missing BUSCO	2.4%	3.8%	3.1%

To assess the quality of the blue catfish reference genome assembly, we compared blue catfish transcriptome and DNA methylome data alignment rate to channel catfish reference genome versus this blue catfish assembly. For RNA-seq, adult liver transcriptome data from our previous research were used with NCBI GEO (Gene Expression Omnibus) databases, accession number GSE186603 [19]. For DNA methylome data, DNA was extracted from 3 cryopreserved blue catfish sperm samples using AllPrep PowerFecal DNA/RNA Kit (Qiagen, Redwood City, CA, USA) following the manufacturer's protocols. Pair-end Enzymatic Methyl-Seq (EM-seq) libraries were constructed using NEBNext Enzymatic Methyl-seq Kit (New England BioLabs). The quality and size distribution of libraries were determined by TapeStation 4200 (Agilent Technologies), before they were sequenced in an Illumina NovaSeq 6000 S4 lane at Novogene (Novogene Corporation Inc.). The raw EM-seq reads were checked by FastQC (FastQC, RRID:SCR_014583) [72] and trimmed by Trimmomatic (version 0.36) [49], with the parameters “ILLUMINACLIP:adapter:2:30:10 LEADING:3 TRAILING:3 SLIDINGWINDOW:4:15 MINLEN:75.” The filtered reads were aligned to blue catfish genome assembly using the BWA aligner (version 0.7.17-r1188) (BWA, RRID:SCR_010910) [73], with parameters “bwa-mem2 mem -t 48 -M -w 3 -O 12 -E 4.”

### Repeat annotation

To compare the quality of blue catfish genome with related species genome, RepeatModeler version 2.0.1  (RepeatModeler, RRID:SCR_015027) [74] was performed to identify the repetitive elements in our blue catfish genome assembly, channel catfish (*I. punctatus*) genome assembly [67], and tra catfish (*P. hypophthalmus*) genome [68]. The interspersed repeat sequences and low-complexity DNA sequences were masked with RepeatMasker version 4.0.6  (RepeatMasker, RRID:SCR_012954) [75].

### SNP identification between channel catfish and blue catfish, and within blue catfish between D&B and Rio Grande strains

A total of 18.6 million PacBio CCS HiFi reads generated from the blue catfish D&B strain were aligned to the channel catfish reference genome version 1.2 [23] using Minimap2  (Minimap2, RRID:SCR_018550) [76, 77]. *De novo* SNP calling was performed in the LongRanger pipeline v2.1.6 using UnifiedGenotyper in GATK version 3.6 (GATK, RRID:SCR_001876) [78] with “-stand_call_conf 50.0 -stand_emit_conf 10.0” parameters. SNP positions with a coverage depth of less than 10 were excluded from subsequent analysis. The 10× Genomics reads from 2 individuals (female and male) of the Rio Grande strain and 2 individuals (female and male) of the D&B strain were aligned to the repeat masked assembly of blue catfish using LongRanger software version 2.1.6 [24]. *De novo* SNP calling was performed in the LongRanger pipeline using GATK version 3.4 with the default parameters [78]. The insertion and deletion variants in the Variant Call Format (VCF) file were filtered out by BCFtools version 1.11  (SAMtools/BCFtools, RRID:SCR_005227) [79]. To obtain high-quality SNPs, positions with mapping quality <250 and coverage depth <4 were excluded from the analysis. The shared high-quality SNPs between the female and male samples were kept for the D&B and the Rio Grande strains.

### Gene prediction and functional annotation

To annotate the protein-coding genes in the blue catfish genome, we exploited *ab initio*, RNA-seq-based, and homology-based approaches for gene prediction in the repeat-masked assembly. Trimmed RNA-seq reads were mapped to the blue catfish genome assembly with Tophat version 2.1.1 [80], and transcript isoforms were extracted using Cufflinks version 2.2.1 (Cufflinks, RRID:SCR_014597) [81]. In addition, *de novo* transcript contig assembly was performed using Trinity version 2.4.0  (Trinity, RRID:SCR_013048) [82]. The blue catfish repeat families and transcriptome assembly were fed to the MAKER annotation pipeline version 2.31.9  (MAKER, RRID:SCR_005309) [83]. Gene models were predicted using *ab initio* gene prediction algorithms with protein and transcriptome evidence by EST2GENOME and PROTEIN2GENOME procedures in MAKER. The RNA-seq GFF3 file and transcript assembly were provided as expressed sequence tag (EST) evidence, and annotated protein sequences of teleost species in the OrthoDB database version 9.1 were utilized as homology evidence [84]. The initially predicted gene models were used to train both the SNAP (SNAP—SNP Annotation and Proxy Search, RRID:SCR_002127) [85] and the AUGUSTUS (Augustus, RRID:SCR_008417) [86, 87] gene predictors. Two additional iterations were performed to generate the final MAKER gene models. For homology-based gene prediction, high-quality channel catfish protein-coding gene models were downloaded from the Ensembl database version 99 [88], and blue catfish gene prediction was performed using Gene Model Mapper (GeMoMa, RRID:SCR_017646, version 1.8) [89], based on the channel gene catfish model and BAM files of blue catfish RNA-seq alignments. Finally, the GeMoMa and MAKER gene sets were compared and merged to select the best representative gene models. To assess the quality of the gene annotation, the sequences of predicted gene models were aligned to the EST sequences from blue catfish using BLAT  (BLAT, RRID:SCR_011919) [90].

### Annotation of noncoding RNA genes

Noncoding RNA genes were predicted by the Rfam (Rfam, RRID:SCR_007891)/INFERNAL version 1.1.4  (Infernal, RRID:SCR_011809) [91] (accessed on 30 January 2022) using Rfam database version 14.7 [92]. The transfer RNA (tRNA) gene models were identified using tRNAscan-SE version 2.0.9 (tRNAscan-SE, RRID:SCR_010835) implemented in the MAKER pipeline [93]. For 28S and 18S ribosomal RNA (rRNA) genes, fragment gene models were excluded from the analysis.

### Catfish 690 K probe alignment to channel and blue catfish genomes

The widely applied catfish 690 K SNP array using Affymetrix Axiom technology [35] was evaluated for SNP coverage in the blue catfish genome. The 5′- and 3′-SNP flanking sequences in the probe were aligned to the channel catfish and blue catfish genome using in the UCSC In-Silico PCR tool with 11-bp tileSize and 30-bp minimum perfect match. *In silico* PCR hits less than or greater than 71 bp or with indels in them were excluded. Channel catfish and blue catfish alleles were determined for the SNP position in each probe. Density patterns of channel–blue SNPs covered by the catfish 690 K array were plotted across each chromosome using the CMplot package in R [94].

### Comparative genome analysis

To compare the genome assembly in blue catfish and channel catfish, the chromosome ideogram was drawn according to a previous karyotyping study [95]. The blue catfish genome sequences were compared with that of channel catfish genome sequences to identify chromosome orthology. Multiple genome alignment and visualization were performed using Mauve version 2.1.0 [96] implemented in Geneious version 11.1.5 (Geneious, RRID:SCR_010519) [97]. Unique genomic regions with high sequencing similarity and genome rearrangement events between blue catfish and channel catfish were highlighted for each chromosome pair comparison. In addition to DNA sequences, we also compared the 2 genomes using protein-coding genes as anchors. Homologous regions in these 2 genomes were identified using MCScan version X  (MCScan, RRID:SCR_017650) [98], a Python package for synteny detection and evolutionary analysis. The inferred gene pairs and linked relationships were visualized and placed in the context of whole-genome collinearity using a genomic circle generated by Circos version 0.69–7  (Circos, RRID:SCR_011798) [99].

### Phylogenetic analysis

To investigate the phylogenetic relationship between blue catfish and other Actinopterygii fish, 10 species were selected from 52 Actinopterygii species in OrthoDB version 10.1 (OrthoDB, RRID:SCR_011980) [100], including channel catfish (*I. punctatus*), Atlantic herring (*Clupea harengus*), zebrafish (*Danio rerio*), northern pike (*Esox lucius*), large yellow croaker (*Larimichthys crocea*), spotted gar (*Lepisosteus oculatus*), Nile tilapia (*Oreochromis niloticus*), guppy (*Poecilia reticulata*), greater amberjack (*Seriola dumerili*), and pufferfish (*Takifugu rubripes*). A total of 5,269 single-copy 1:1 orthologs among these species were identified. Protein sequences were aligned using MAFFT version 7.407 (MAFFT, RRID:SCR_011811) [101] and concatenated into one alignment for phylogenomic analysis. IQ-TREE version 1.6.12 (IQ-TREE, RRID:SCR_017254) [102] was used to estimate the best protein model for phylogenetic tree construction. A maximum likelihood (ML) tree was built with the best-fit model JTT+F+R3, with 1,000 bootstraps or branch support evaluation. The phylogenetic tree was annotated and visualized using FigTree version 1.4.4 (FigTree, RRID:SCR_008515) [103].

### Gene family expansion and contraction analyses

The gene models from the blue catfish and 10 other Actinopterygii species in the phylogenomics analysis were obtained from this assembly and OrthoDB version 10.1 [100], respectively. Protein sequences less than 10 amino acids were excluded from the analyses. Sequence alignments were performed by all-versus-all blast module using Diamond version 2.0.0 (DIAMOND, RRID:SCR_009457) [104], with an E-value cutoff of 1×10^–5^. Gene family clusters were identified using OrthoMCL (version 2.0.9) [105]. The divergence time was estimated based on the phylogenetic tree and protein sequence by r8s version 1.8.1  (r8s, RRID:SCR_021161) [106]. The penalized likelihood (PL) method and truncated Newton optimization (TN) algorithms were used to estimate divergence time and absolute rates of substitution. Three calibration nodes were applied for estimating divergence time, including 1 fixed time point (*I. punctatus* and *Danio rerio*, 142 million years ago) and 2 constraining time points (*Poecilia reticulata* and *Oreochromis niloticus*, 88–139 million years ago and *Larimichthys crocea* and *Takifugu rubripes*, 102–127 million years ago) from the TimeTree (TimeTree, RRID:SCR_021162) [107]. Based on estimated divergence times and phylogenetic relationships, CAFE version 4.2.1  (CAFE, RRID:SCR_005983) [108] was used to analyze the gene family expansion and contraction. The cutoff for significantly changed gene families was a *P*value less than 0.05. The genes in significantly expanded gene families were annotated by orthology in other species from OrthoDB version 10.1 or the eggNOG-mapper version 2.1.7 (eggNOG-mapper, RRID:SCR_021165) [109].

## Results

### Genome assembly and statistics

**Figure 1.**
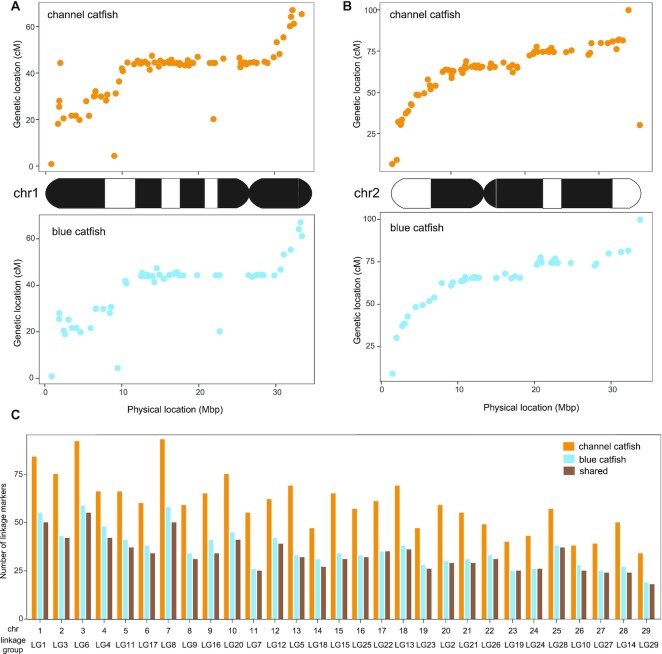
Markers in the genetic linkage map of channel catfish, *Ictalurus punctatus*, and blue catfish, *I. furcatus*. (A) The linkage map position (*y*-axis) and physical location (*x*-axis) of 84 channel catfish genetic makers and 55 blue catfish genetic markers on chromosome 1. (B) The linkage map position (*y*-axis) and physical location (*x*-axis) of 76 channel catfish genetic makers and 43 blue catfish genetic markers on chromosome 2. (C) A total of 1,739 and 1,009 molecular markers were anchored onto 29 chromosomes in channel catfish (orange bar) and blue catfish (blue bar), respectively. The brown bars represent the shared makers in both channel catfish and blue catfish.

The estimated total genome length of *I. furcatus* is 839,021,413 bp, based on the *k*-mer distribution and depth in the Illumina short-read data generated in this study, and the inferred overall rate of heterozygosity is 0.597%. A total of 316.9 Gb PacBio raw reads were generated from the blood DNA of a single female blue catfish (D&B strain). Genome assembly was performed using 21.3 Gb PacBio CCS HiFi reads, and a total of 563 contigs were assembled, which was much fewer than the long-read assemblies of channel catfish and tra catfish genomes (Table 1 and Supplementary Table S1). The assembled contigs had an N50 of 8.59 Mb, indicating excellent contiguity. The contigs were further assembled into chromosomes based on linkage markers and linkage map information constructed using F_1_ backcrosses in 2016 [61]. Both species had 29 pairs of homologous chromosomes, and no karyotype differences can be detected at the cytogenetic level [57, 95]. The genetic maps and physical distances were consistent between blue catfish and channel catfish chromosomes (Fig. 1A–B). On average, channel catfish had 60.0 linkage markers per chromosome, whereas blue catfish assembly had 34.8 markers, with 32.8 overlapped in both species (Fig. 1C and Supplementary Data S1). Given that the number of markers was much larger than the number of scaffolds per chromosome (*n* = 11), the channel–blue linkage markers were sufficient for the chromosomal assembly. The final assembly consisted of 29 chromosomes, a circularized mitochondrial genome, and 241 unplaced contigs. The final genome size was 841,864,377 bp, with a scaffold N50 of 28.24 Mb (Table 1).

### Assessment of genome completeness

**Figure 2.**
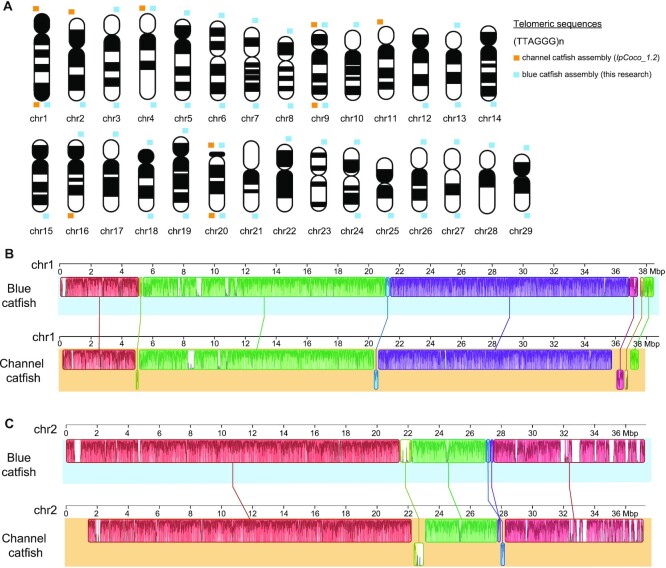
Synteny alignments of blue catfish and channel catfish chromosomes based on DNA sequence similarity. (A) Presence and absence of telomeric repeat motif (TRM) at termini of assembled chromosomes shown in karyogram. The orange boxes represent channel catfish telomere, and blue boxes represent blue catfish telomeric assembly. (B, C) Synteny analysis of chromosomes 1 and 2 between channel catfish and blue catfish (synteny analyses for remaining chromsomes are shown in Supplementary Fig. S2).

The completeness of blue catfish genome assembly was assessed using the BUSCO tool (see Materials and Methods). The completeness score against Actinopterygii BUSCO was 97.0%, which was slightly higher than the channel catfish (95.0%) and tra catfish (96.1%; see Table 1). The proportions of duplicated or fragmented BUSCO genes were low in the blue catfish genome, suggesting a highly complete reference assembly (Table 1). Similar results were obtained when assessed using Eukaryota BUSCOs (Table 1). We examined the termini of the assembled chromosomes and discovered that 43 of the 58 telomeres start or end with the vertebrate-type telomeric repeat motif TTAGGG, indicating telomere-to-telomere continuity for the majority of the chromosomes (Fig. 2A and Supplementary Table S2). In contrast, only 10 telomeric regions were present in the channel catfish assembly (Fig. 2A and Supplementary Table S3). Over 90% of the 10× Genomics reads from the female D&B library can be uniquely aligned to the blue catfish assembly, and 99.5% of the blue catfish genome was covered by the Illumina reads (Supplementary Table S1).

### Protein-coding gene annotations

A total of 17,945 gene models were annotated by SNAP, and 10,591 genes were predicted by AUGUSTUS in the MAKER pipeline (see Materials and Methods). Homology-based GeMoMa algorithm identified 20,460 protein-coding genes based on channel catfish gene models in Ensembl version 99. These gene models were merged based on BLAT results to the blue catfish genome assembly, resulting in 33,677 predicted protein-coding genes. Among these gene models, 30,971 (92.0%) are complete with a start codon and a stop codon. The average coding region length was 1,195 bp, and the mean number of coding exons per gene model was 7.2. There were a total of 21,781 genes with RNA-seq reads aligned to the gene region (Supplementary Table S4), and 21,330 gene models were supported by blue catfish EST sequences.

### Annotation of noncoding RNA genes

A total of 6,192 tRNAs were identified using tRNA-scan (Table 2 and Supplementary Data S2). Among them, 1,712 decode the 20 standard amino acids, 8 decode selenocysteine (TCA), and 10 are predicted suppressor tRNAs. The remaining ones are predicted pseudogenes. A total of 55 complete clusters of 18S–5.8S–28S rRNA were predicted (Table 2 and Supplementary Data S3) and located on chromosome 4 and 4 unplaced scaffolds (chrUn008, chrUn028, chrUn032, chrUn034). The blue catfish 28S rRNA gene has a 99.2% sequence identity with the channel catfish gene on chromosome 24 and a 92.4% identity with tra catfish (XR_004 577 711). The blue catfish 18S rRNA gene has a 98.8% sequence identity with channel catfish (AF021880) and a 98.3% identity with tra catfish (XR_004577708). The blue catfish 5.8S rRNA genes have a 100% sequence identity with channel catfish (GQ465242) and a 99.4% identity with tra catfish (XR_003402644). A total of 12,448 copies of predicted 5S rRNA genes were identified (Table 2), 95.6% of which were located in a 4-Mbp cluster on the chromosome 14 subtelomeric region between positions 29,127,406 bp and 33,137,707 bp (Supplementary Data S3). Spliceosome-related small nuclear RNAs (snRNAs) include U1, U2, U4, U5, U6, and other members (Supplementary Data S4). Altogether, there were 601 predicted copies of snRNAs (Table 2), and they were organized in major clusters in the blue catfish genome (U1 on chr1, U2 on chr19, U4 on chr14, U5 on chr19, and U6 on chr22). Small nucleolar RNAs (snoRNAs) are responsible for nucleotide modifications in rRNAs. A total of 135 C/D-box snoRNAs and 72 H/ACA-box snoRNAs were predicted in the blue catfish genome (Table 2 and Supplementary Data S5). A total of 2,079 pre–microRNA (miRNA) genes were predicted in the blue catfish genome (Table 2 and Supplementary Data S6), which encode 105 putative miRNAs.

**Table 2:** Summary of predicted noncoding RNA genes annotated in blue catfish, *Ictalurus furcatus*, genome

Noncoding RNAs	Counts (full copies)	Average unit length	Total length	Percent of genome
**tRNA genes**				
tRNAs decoding standard 20 amino acids	1,712	75 bp	128,864 bp	0.015%
Selenocysteine tRNAs	8	75 bp	597 bp	<0.001%
tRNAs with undetermined isotypes	13	73 bp	953 bp	<0.001%
Suppressor tRNAs	10	81 bp	813 bp	<0.001%
Predicted pseudogenes	4,359	75 bp	338,738 bp	0.040%
**tRNA total**	6,102	—	469,965 bp	0.056%
**rRNA genes**				
28S rRNA	55	4,180 bp	229,935 bp	0.027%
18S rRNA	55	1,872 bp	102,983 bp	0.012%
5.8S rRNA	57	154 bp	8,782 bp	0.001%
5S rRNA	12,448	117 bp	1,451,889 bp	0.173%
**rRNA total**	12,615	—	1,793,589 bp	0.214%
**snRNA genes**				
U1	74	163 bp	12,047 bp	0.001%
U2	215	215 bp	39,363 bp	0.005%
U4	42	141 bp	5,903 bp	0.001%
U5	207	115 bp	23,753 bp	0.003%
U6	51	106 bp	5,390 bp	0.001%
Other	12	105 bp	1,258 bp	<0.001%
**snRNA total**	601	—	87,714 bp	0.010%
**snoRNA genes**				
C/D-box snoRNA	135	123 bp	17,611 bp	0.002%
H/ACA-box snoRNA	72	150 bp	9,572 bp	0.001%
**snoRNA total**	207	—	27,183 bp	0.003%
**miRNA genes**	2,079	81 bp	167,979 bp	0.020%

### Ancient whole-genome duplication in blue catfish

Teleost species underwent a fish-specific ancient whole-genome duplication (WGD) event during evolution [110]. To investigate this, we performed synteny analysis within the blue catfish genome. A significant amount of reminiscent paralogy was found in a few chromosome pairs (chr4–chr5, chr9–chr25, and chr15–chr21), as well as local regions among other chromosomes (Supplementary Fig. S1).

### Comparative genomics between the blue catfish and the channel catfish

**Figure 3.**
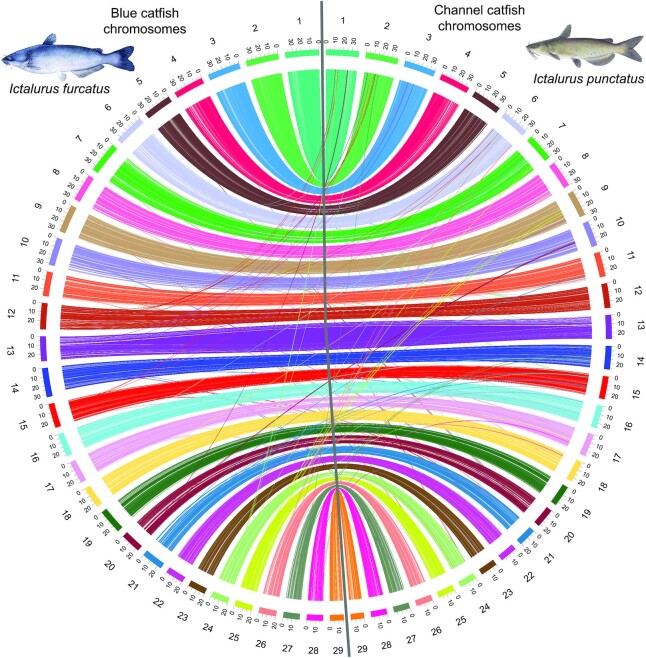
Genome comparisons between channel catfish, *Ictalurus punctatus*, and blue catfish, *I. furcatus*, based on orthologous genes. A total of 29 chromosomes in the blue catfish genome assembly (on the left in the Circos plot) showed a one-to-one homologous relationship with 29 chromosomes in the channel catfish genome (on the right in the Circos plot). The outside ring represents chromosomes.

Comparative genomic analysis was performed using the whole-genome alignment tool Mauve (see Materials and Methods), and blue–channel pairwise chromosomal alignment revealed large syntenic blocks between homologous chromosomes (Fig. 2B–C and Supplementary Fig. S2). For example, chromosome 1 only had 4 inversion events involving small regions near the centromere and telomere (Fig. 2B). Local genome expansions and contractions were observed, but they only accounted for a small fraction of the genome. Gene model and gene order–based comparisons of the 2 genomes were also performed (Fig. 3), and a high level of synteny between the corresponding chromosomes was also detected, with a few minor genome rearrangements (Fig. 3).

### Divergence between D&B strain and Rio Grande blue catfish strains

Through *de novo* SNP calling using GATK, we identified a total of 1,433,465 SNP positions between the D&B and the Rio Grande strains. Among these, 607,059 were fixed differences between D&B and Rio Grande, and 826,406 were segregating within the Rio Grande strain. We estimated that the intraspecific SNP density between these 2 strains was 0.0032. The assembled and circularized D&B mitochondrial (MT) genome is 16,499 bp in length, which is the same length as a previously assembled blue catfish MT genome (NCBI GenBank accession number NC_028 151). We identified 1 fixed nucleotide difference between our D&B MT genome assembly and NC_028 151 (C12307T), which is a synonymous substitution at the third position in a codon encoding Phe. We assembled the first MT genome in the Rio Grande strain, and the circularized genome size is also 16,499 bp. There are 53 substitutions between D&B and Rio Grande (36 in protein-coding genes, 2 in rRNA, and 2 in tRNA genes), resulting in a sequence divergence of 0.0032 in the entire genome and 0.0026 in the genic region (Supplementary Table S5).

### SNP annotation and channel genome-based pseudo–blue catfish genome

**Figure 4.**
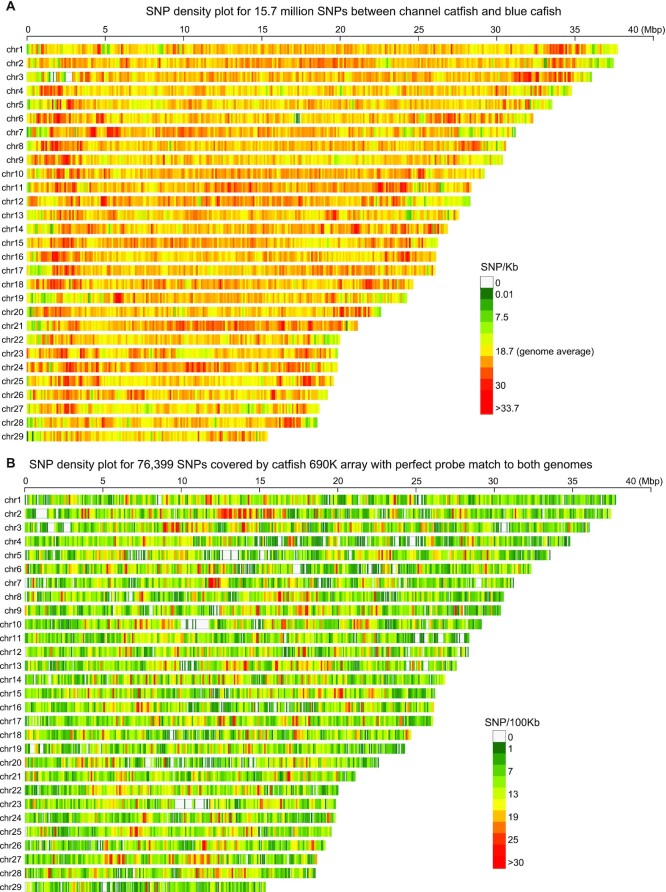
Density plots of channel–blue catfish (*Ictalurus*) genome-wide SNPs and informative SNPs covered by catfish 690 K array. (A) SNP density plot showing the distribution of polymorphisms per kilobase pairs across 29 chromosomes. The scale for the number of SNPs is shown on the right. (B) Density plot showing the distribution of informative, perfect-match SNP probes per 100 kilobase pairs across 29 chromosomes. The scale for the number of SNPs is shown on the right.

The interspecific sequence differences between channel catfish and blue catfish were previously estimated to be 13 to 15 SNPs per Kb based on EST data [111]. In this study, with the blue catfish genome assembly and resequencing data of 2 important aquaculture strains (D&B and Rio Grande), *de novo* SNP calling revealed a total of 15,685,661 fixed differences between the blue catfish and the channel catfish, which is 18.7 SNPs per Kb. This genome-wide estimation is higher than in the more conserved EST sequences. Regions of lower than average SNP density were often located at the chromosome ends (Fig. 4A). The blue catfish and channel catfish MT genomes have a total of 1,041 fixed differences in the coding region (9.1% coding sequence divergence; Supplementary Table S6). Among these substitutions, only 79 (0.069% in the coding region) were nonsynonymous, suggesting strong purifying selection.

### Evaluation of the catfish 690 K SNP array on blue catfish genome

The 690 K catfish SNP array was designed in 2017 [35], incorporating SNP information from the channel catfish reference genome [23] and tissue-specific RNA-seq data sets, as well as blue catfish RNA-seq and Genotyping-by-sequencing (GBS) data. To validate the probes in the newly assembled blue catfish genome, the probes were aligned to both channel and blue reference genomes. A total of 99.0% of 690 K probes were aligned to the channel catfish genome, and 98.2% had a unique perfect hit without any indels or mismatches in the flanking probe sequences (Table 3), suggesting excellent accuracy. When these probes were mapped to the blue catfish genome, 29.3% were mapped, and only 23.9% of probes had unique perfect hits (Table 3). Among these probes, only 76,399 (11.0% of 690 K) overlapped with a channel–blue catfish SNP position (Table 3). These interspecific informative SNP probes were sparsely distributed across the chromosomes (Fig. 4B), with <5 SNPs per 100 Kb for 20% of the genome.

**Table 3:** Evaluation of 690 K catfish SNP array probes for the channel catfish, *Ictalurus punctatus*, genome, blue catfish, *I. furcatus*, genome, and channel–blue informative SNPs

Catfish 690 K SNP array statistics	Channel catfish (IpCoco v1.2)	Blue catfish (this assembly)
Total number of probes	693,567	693,567
Number (%) of probes mapped to genome	686,675 (99.0%)	203,559 (29.3%)
Number (%) of probes mapped to genome without indels	685,939 (98.9%)	201,580 (29.1%)
Number (%) of probes mapped to multiple regions	4,560 (0.7%)	1,921 (0.9%)
Number (%) of probes with mismatches in SNP flanking sequence	677 (0.1%)	33,731 (16.8%)
Number (%) of probes with unique/perfect hit (**valid set**)	680,856 (98.2%)	165,928 (23.9%)
Number (%) of probes for channel–blue SNPs (**informative set**)	253,292 (36.5%)	76,399 (11.0%)
Number (%) of probes for blue catfish within-species SNPs	—	2,978 (1.80%)

### Improvement of transcriptome and DNA methylome alignments to the newly assembled blue catfish genome

For the blue catfish liver RNA-seq data generated in our previous research, only 65.4% of RNA-seq reads can be aligned to the channel catfish reference genome [67] due to the reference bias (Supplementary Table S4). We constructed a pseudogenome for blue catfish based on the channel reference genome IpCoco v1.2, by replacing the channel alleles with blue alleles at the 15,685,661 channel–blue SNP positions we identified. The average RNA-seq alignment rate improved to 71.8%, with a 9.8% increase in uniquely mapped reads. However, the pseudogenome mapping rate is still relatively low, which may result in poor coverage for some blue catfish genes (Supplementary Table S4). When the newly assembled blue catfish genome was used as the reference, the average percentage of uniquely mapped reads increased to 85.1% (a 30% increase compared to using the channel reference), which maximized the read coverage for expressed genes (Supplementary Table S4). Blue catfish sperm DNA methylome sequence mapping rate was also significantly improved using the blue catfish genome assembly, with ∼80% of the EM-seq reads uniquely mapped to the blue catfish genome.

### Repeat annotation

Repetitive sequence annotation identified that 47% (395.5 Mb) of the blue catfish genome belongs to repetitive regions (Table 4), which was in between the channel catfish (51%) [112] and tra catfish (40%). Among the known repeats in blue catfish, the DNA transposon superfamily *pogo* was the most abundant, accounting for 43% of all known repeats, or 8.2% of the entire genome (Table 4). *Pogo* has the bacteria *IS630* transposase and was repeatedly domesticated in vertebrates, including fish [113]. Channel catfish genome also had 7.8% of *Pogo* repeats, but it is less abundant in tra catfish (4.7%). The following classes of repeat accounted for more than 1% of the blue catfish genome: LINEs (3.0%), Gypsy/DIRS1 elements (2.3%), L2/CR1/Rex clade (1.9%), and simple repeats (3.3%).

**Table 4:** Summary repeat element classes in blue catfish (*Ictalurus furcatus*), channel catfish (*Ictalurus punctatus*), and tra catfish (*Pangasianodon hypophthalmus*) genomes

	*Ictalurus furcatus* (this assembly)		*Ictalurus punctatus* (ASM400665v3)		*Pangasianodon hypophthalmus* (GCA_016801045.1)	
	# of elements	Length (%)	# of elements	Length (%)	# of elements	Length (%)
**Retroelements**						
Penelope	1,717	838,078 (0.1%)	1,214	922,008 (0.09%)	1,988	488,401 (0.07%)
LINEs	58,734	24,968,273 (2.96%)	89,843	35,225,182 (3.53%)	47,975	18,520,943 (2.49%)
L2/CR1/Rex	43,100	16,253,105 (1.93%)	57,766	20,111,601 (2.01%)	37,713	14,893,881 (2.01%)
R1/LOA/Jockey	763	350,294 (0.04%)	2,542	1,043,469 (0.1%)	537	175,472 (0.02%)
R2/R4/NeSL	592	266,497 (0.03%)	691	234,736 (0.02%)	576	228,226 (0.03%)
RTE/Bov-B	3,485	1,381,797 (0.16%)	4,718	1,666,345 (0.17%)	4,156	1491,021 (0.02%)
L1/CIN4	2,354	2,261,930 (0.27%)	10,403	4,543,711 (0.46%)	799	348,555 (0.05%)
**LTR elements**						
BEL/Pao	380	241,716 (0.03%)	207	336,942 (0.03)	193	177,582 (0.02%)
Retroviral	4,006	3,275,694 (0.39%)	3,719	4,848,411 (0.49%)	497	398,835 (0.05%)
Gypsy/DIRS1	32,774	19,609,302 (2.33%)	39,817	22,167,664 (2.22%)	21,919	9,258,407 (1.25%)
**DNA transposons**						
hobo-Activator	21,792	5,726,207 (0.68%)	18,044	5,537,205 (0.55%)	10,850	2,191,374 (0.3%)
Tc1-IS630-Pogo	189,344	68,689,788 (8.16%)	213,390	78,079,336 (7.82%)	112,905	34,735,533 (4.68%)
PiggyBac	906	347,427 (0.04%)	552	215,961 (0.02%)	323	138,058 (0.02%)
Tourist/Harbinger	2,084	586,919 (0.07%)	3,613	589,965 (0.06%)	2,226	637,457 (0.09%)
**Unclassified**	958,235	219,639,183 (26.08%)	1,197,919	263,899,746 (26.43%)	932,206	186,441,198 (25.11%)
**Simple repeats**	575,417	27,950,071 (3.32%)	706,627	34,869,604 (3.49%)	544,794	28,043,320 (3.78%)
**Low complexity**	43,673	3,139,550 (0.37%)	52,048	3,441,656 (0.34%)	42,087	2,746,467 (0.37%)
**Total**	1,939,356	395,525,831 (46.96%)	2,403,113	477,733,542 (50.8%)	1,761,744	300,914,730 (40.36%)

### Phylogenomic analysis with teleost genomes

**Figure 5.**
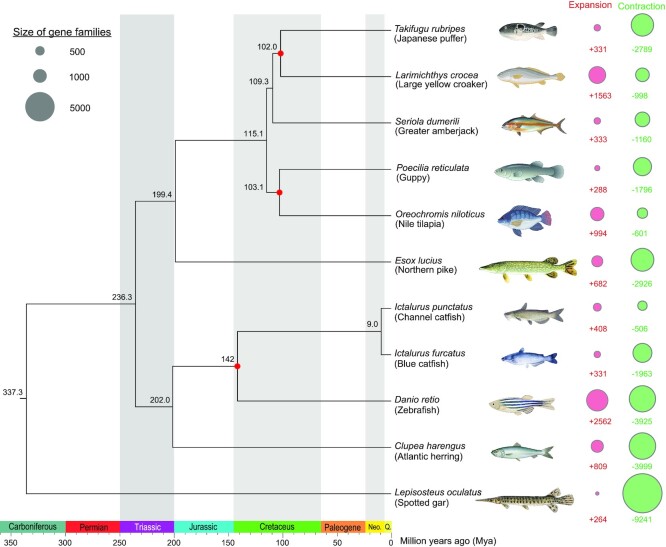
Phylogenomic and gene family expansion and contraction analysis of blue catfish *Ictalurus furcatus*. A maximum likelihood phylogenetic tree of *Ictalurus furcatus* with 10 other Actinopterygii species was constructed based on 4,698 shared 1:1 single-copy proteins using IQ-TREE. The bootstrap values are supported at 100/100 for all branching points. Gene family evolution was analyzed using CAFÉ. The number of gene family expansions and contractions is shown in a bubble plot for each species. The species divergence time was estimated and labeled at each branch site (millions of years ago). The calibration nodes for divergence time are labeled in red from fossil evidence.

To understand the phylogenetic relationship of *I. furcatus* with other Actinopterygii species, we used 4,698 single-copy 1:1 orthologs to construct a phylogenetic tree of 11 species (see Methods). The phylogenomic analysis provides highly supported internal branches with 100 bootstrap values (Fig. 5). The blue catfish fall into the same clade with channel catfish, which is consistent with the known phylogenetic relationships (Fig. 5).

### Gene family expansion and contraction in blue catfish genome

To reveal the gene family evolution among blue catfish and 10 other fish species, divergence times and gene family expansion/contraction were determined for each species (see Methods). Phylogenomic analysis indicated that the divergence of blue catfish (*I. furcatus*) and channel catfish (*I. punctatus*) occurred approximately 9 million years ago, according to 4,698 single-copy ortholog genes in these 11 species (Fig. 5). A total of 25,027 gene families were examined for expansion/contraction analysis. In blue catfish, 331 expanded gene families and 1,963 contracted families were identified (Fig. 5 and Supplementary Table S7). Among them, 50 families underwent rapid expansion (Supplementary Table S8), containing 263 blue catfish genes (Supplementary Data S7). These expanded gene families include immunoglobulin domain proteins (9 families and 44 genes) and gene families involved in immune functions (9 families and 59 genes; see Supplementary Table S8). Seven families of transposable element genes (*n* = 55), such as reverse transcriptases and transposases, are the second-largest category of rapidly expanded families.

## Discussion

### A chromosome-level genome assembly of an aquaculturally important species

In this study, we assembled and reported the first blue catfish genome using a combination of Pacific Biosciences CCS (circular consensus sequence) and 10× Genomics linked-read technologies. The final assembly is chromosome-level, telomere-to-telomere for many chromosomes, with scaffold N50 of 28.2 Mbp and contig N50 of 8.6 Mbp. The BUSCO completeness score is 98.4% when assessed using Eukaryota BUSCO genes and 97.0% using Actinopterygii BUSCOs. The assembly statistics data indicate that our assembly is excellent in terms of both genome continuity and completeness.

### High-quality blue catfish genome and well-annotated gene catalog provide the necessary reference genome for genotyping, genome editing, transcriptome, and methylome analyses

Previously, the channel catfish genome was used as the reference for blue catfish sperm RNA-seq and DNA methylome analyses, which had significantly lower mapping rates, and many critical blue catfish genes were not covered due to higher divergence. Our novel blue catfish assembly dramatically improved the sequencing alignment rate for RNA-seq, Methyl-seq, and genome resequencing data in blue catfish. Using computational prediction and RNA-seq evidence-based approaches, we identified a total of 33,686 protein-coding genes. Of these, over 20,000 genes are with RNA-seq and blue catfish EST support. The number of gene models is comparable to the channel catfish annotation [23], which is a well-annotated genome. Genetic enhancement of catfish production has been under way using MAS, and further improvement could be achieved through targeted gene editing in the hybrid catfish background, which has gained great momentum in aquaculture. In channel catfish, the CRISPR-Cas system has been successfully applied to understand genetic regulation and mechanisms of economically important traits and attributes, including the chemokine C-C motif ligand 33 in barbel regeneration [114], myostatin in body weight regulation [115], an exogenous alligator cathelicidin gene in pathogen defense [116], and interleukin 1 receptor and lectin genes for improved immune response and disease resistance [117]. The availability of a high-quality channel catfish reference genome provided essential information for gRNA design, homologous arms, and noncoding region screening. In hybrid catfish, many superior alleles came from the blue catfish. Without the blue catfish reference genome sequence, it is not possible to design gRNAs and predict potential off-target effects for genome editing to improve economically important traits. Furthermore, one of the major limiting factors in hybrid catfish reproduction is the lack of technical standards for sperm collection and quality assessment. DNA methylation is the most reliable indicator of sperm quality and ensuing offspring performance. Research is under way to elucidate the utility of DNA methylation status for the prediction of hatch rate and inform the industry of best practices. The availability of this high-quality blue catfish genome is critical to enable the paternal side genetic and epigenetic enhancement of hybrid catfish genetic types.

### Blue catfish genome assembly facilitates the understanding of environment-dependent heterosis, an intriguing phenomenon in hybrid catfish

The phenomenon of heterosis was first reported by Charles Darwin [118] and has been under extensive study by evolutionary biologists since then. Hybrids of blue catfish sire and channel catfish dam are superior in many disease resistance and production traits. The molecular mechanism of heterosis is worth investigating. However, without a blue catfish genome and a genome-wide SNP data set, it is impossible to accurately profile allele-specific gene expression and DNA methylation in hybrids. In this study, we identified over 15 million SNPs between the channel catfish and the blue catfish, and the number of SNPs in the transcripts and regulatory regions is sufficient for genome-wide profiling of parent-of-origin expression [119]. The availability of blue catfish reference genome will help elucidate the regulatory and epigenetic mechanism of environment-dependent heterosis in hybrid catfish.

### The blue catfish genome provides new insight into blue–channel divergence and Siluriformes evolution

In the present study, we estimated that blue catfish diverged from channel catfish approximately 9 million years ago, based on 1:1 single-copy orthologs. The estimate is significantly more recent than a previous prediction (16.6 million years) based on the divergence of the cytochrome b gene alone [112], which might be an overestimation due to the accelerated substitution rate in the mitochondrial genome. The comparative genomic analysis confirmed that the 29 chromosomes are mainly synteny between the channel catfish and blue catfish with a few local rearrangements. Phylogenomic analysis of reference fish genomes in OrthoDB revealed that zebrafish was the closest outgroup species to catfish species, which is congruent with the known phylogeny of ray-finned fishes [120] and is also consistent with previous findings that the most closely related model fish species is zebrafish [121]. Many genomes are available in Siluriformes, including the Asian redtail catfish (*Hemibagrus wyckioides*) [122] and yellow catfish (*Tachysurus fulvidraco*) [123] in the family of Bagridae, giant devil catfish (*Bagarius yarrelli*) in Sisoridae [124], 12 Pangasiidae species [68–70], 3 Clariidae species [125–127], and channel catfish (*Ictalurus punctatus*) and black bullhead catfish (*Ameiurus melas*, GCA_012411365) in the family of Ictaluridae. Our blue catfish genome will provide an invaluable resource to investigate molecular phylogeny and comparative analysis in Siluriformes.

### Comparative analysis of channel and blue catfish genomes reveals subchromosomal level differences and the expansion of immune function related genes in blue catfish

Although blue catfish and channel catfish have morphologically indistinguishable chromosomes with essentially identical Giemsa banding patterns [57, 95], in this study, we identified 15 million SNPs and demonstrated structural rearrangement events between the 2 species, as well as differences in gene and repeat content. These genetic differences may explain the myriad of species-specific traits related to growth, disease resistance, body conformation and coloration, the incidence of albino fish, behavior, seinability, age and size of maturity, and responses to hormone-induced spawning.

Interestingly, we identified blue catfish lineage-specific gene family expansions. It is not surprising that transposases and reverse transcriptases are among the rapidly expanding family because of the active TE (transposable elements) turnover. Genes with immune-related functions account for 40% of the known expanded families, including T-cell receptor delta, lectin, complement control proteins, glucocorticoid receptor, chemokine interleukin 8, CD225/Dispanin, and others. This is consistent with our previous findings that the blue catfish had the highest immune activity compared to the channel and hybrid catfish at 10 months of age [19], and the gene family expansion may contribute to the superior phenotypes of lysozyme and alternative complement activities in blue catfish. Further understanding of the evolution of immune-related genes will provide valuable information for the genetic enhancement of hybrid catfish in pathogenic disease resistance.

### A third-generation catfish SNP array is necessary for improved resolution in future GWAS analysis for disease resistance and growth improvement

Pathogenic infection disease is the number one cause of catfish production loss. Paternal genetic contributions from blue catfish are essential for improving industry-relevant traits [18], and F_1_ hybrid catfish can reduce loss from 40% to 20% by carrying disease-resistant alleles from the blue catfish genome. Our research team, along with other researchers, has identified the genetic loci responsible for the resistance of 3 major catfish diseases, Enteric Septicemia of Catfish (ESC), columnaris, and *Aeromonas* diseases [24–27], and in most cases, the blue catfish allele is the resistance allele. Based on the knowledge learned from these GWAS and QTL mapping studies, MAS using F_2_ and F_1_ backcrosses would be an ideal and effective approach to select superior breeders for traits of interest. Choosing the best representative blue–channel SNPs from the GWAS peaks requires a high-quality blue catfish genome due to the following reasons. First, equal PCR amplification efficiency or probe affinity is ideal for the SNP typing assays, and sequence information is needed from both channel and blue genomes for proper primer design. Second, the presence of paralogous sequences will result in spurious SNP calls, and the blue catfish genome is needed to exclude these positions. Last but not least, historical whole-genome duplications in fish genomes further complicate accurate SNP genotyping [128]. Our report in this study meets this urgent need for a high-quality blue catfish genome.

Additional genetic enhancement of the hybrid catfish is essential for better profitability and sustainability. To further improve disease resistance through genomic techniques, we must understand the blue catfish genome in single-base pair resolution. With the new blue catfish assembly, we identified 15 million fixed differences between blue and channel catfish, with a density of 18.7 SNPs per Kb. This is higher than the previous estimation from the blue EST database (13 to 15 SNPs per Kb) [111], which is exactly expected due to higher conservation in transcribed genes. Our study provided a correct genome-wide estimation of the blue–channel divergence and the necessary information for SNP typing primer/probe design. SNP arrays are the widely used approach for cost-effective genotyping experiments. The first-generation 250 K SNP array was designed based on channel catfish sequences [36], and the second-generation 690 K SNP array was designed based on channel catfish reference genome plus blue catfish EST sequences [35]. Because the 690 K array is more comprehensive and replaced the previous generation, we evaluated the probes of the 690 K array using the new blue catfish genome. Only 24% of the probes had unique perfect matches to the blue catfish genome, and more than half of them were targeting invariant positions between the channel catfish and blue catfish genome (some probes were designed to target segregating SNPs within channel catfish [35]). Overall, only 11% of the 690 K SNP array probes are informative for channel–blue SNPs, with fairly uneven distribution in the genome. Therefore, we think a third-generation catfish genotyping array is needed to fully leverage the channel–blue SNPs for MAS and GWAS studies with improved informativeness, genome resolution, and statistical power, which would be versatile for both initial genome screen and fine mapping purposes. The new SNP array could benefit from a gene region-enriched design based on the blue catfish gene models predicted in this study, which may help identify the causal SNPs in coding regions for specific traits of interest.

### SNP analysis between the D&B and Rio Grande strains provides the genetic toolkit for blue catfish and hybrid catfish breed enhancement

The D&B blue catfish strain was widely used in commercial aquaculture, which was obtained originally from rivers in Arkansas, Mississippi, and Texas [129]. D&B was selected for PacBio sequencing because it has been considered the reference strain in farming practices since 2010 [130]. Because the growth and disease resistance advantages of the hybrid catfish are unidirectional (channel catfish female × blue catfish male), researchers must focus on the blue catfish for the reproductive enhancement of the male side. In this context, another blue catfish strain, Rio Grande, which originated from the Rio Grande River, native to Texas [129], was developed by Auburn University [7, 131, 132]. Recent breed development discovered that the Rio Grande strain is superior to D&B in terms of maturation rate, testis size, and the quality/quantity of sperm production [130, 133, 134]. Rio Grande males reach sexual maturity at age 3–5, which is significantly earlier than D&B. Due to these superior traits in reproduction, Rio Grande has been recently included in the USDA–ARS Catfish Genetic Enhancement Program, and USDA is releasing them to stakeholders. In addition, blue catfish strains were shown to have significant variability in disease resistance and mortality [135]. Big differences also exist in growth, body coloration (Rio Grande is the only catfish strain with spots), and seinability. However, the genetic diversity between D&B and Rio Grande was not investigated. To understand the genetic architecture of blue catfish strains, we sequenced the genomes of female and male Rio Grande broodstock and performed genomic analysis. A total of 1.4 million SNPs were identified in the nuclear genome (1.7 per Kb), including 600 K fixed differences between the 2 strains and 826 K SNPs segregating within Rio Grande. The level of genetic diversity is sufficient for the genetic enhancement of male gamete production and potential disease resistance. In contrast, the mitochondrial genome divergence is fairly low (2.6 SNPs per Kb in the genic region), with only 5/36 nonsynonymous substitution between D&B and Rio Grande, suggesting potential purifying selection. The nuclear and mitochondrial SNPs provide informative genetic markers to genotype the D&B and the Rio Grande strains. The genetic background differences between D&B and Rio Grande may explain the variations in sperm quality, male side reproductive traits, growth, body color, and pathogenic disease resistance.

## Data Availability

The draft genome assembly of *Ictalurus furcatus* has been deposited at NCBI under Assembly accession number JAJOLW000000000 and project ID PRJNA785621. Pacbio raw sequencing data have been deposited at NCBI SRA (Sequence Read Archive) under accession number SRR18963096. Illumina sequencing data of the 10× Genomics libraries have been deposited at NCBI SRA under accession numbers SRR18966193, SRR18966194, SRR18966195, and SRR18966196. RNA-seq data were deposited at NCBI with accession numbers SRR16609847, SRR16609846, SRR18989496, and SRR18989495. The mitochondrial genome of the blue catfish D&B strain is submitted to NCBI GenBank under accession number ON022108. The mitochondrial genome of the blue catfish Rio Grande strain is submitted to NCBI GenBank under accession number ON022107. All additional supporting data and materials are available in the *GigaScience* GigaDB database [136].

## Additional Files

**Supplementary Fig. S1**. Circos plot showing paralogous gene pairs in the blue catfish genome.

**Supplementary Fig. S2**. Synteny alignments of blue catfish and channel catfish chromosomes based on DNA sequence similarity.

**Supplementary Table S1**. Summary of PacBio and Illumina (10× Genomics) sequencing data generated for blue catfish genome assembly.

**Supplementary Table S2**. Chromosomal locations of telomeric regions in the blue catfish genome.

**Supplementary Table S3**. Chromosomal locations of telomeric regions in the channel catfish genome.

**Supplementary Table S4**. RNA sequencing data yield, quality control, and alignment statistics to channel catfish and blue catfish genomes.

**Supplementary Table S5**. Summary nucleotide substitutions in the mitochondrial genome between blue catfish D&B and Rio Grande strains.

**Supplementary Table S6**. Summary nucleotide substitutions in the mitochondrial genome between blue catfish and channel catfish.

**Supplementary Table S7**. Summary of gene family expansion and contraction results.

**Supplementary Table S8**. List of gene families underwent rapid expansion in blue catfish.

**Supplementary Data S1**. Locations of 1,800 linkage markers of *Ictalurus punctatus* × *Ictalurus furcatus* crosses in the channel catfish and blue catfish genomes.

**Supplementary Data S2**. Annotation of tRNA genes in blue catfish genome.

**Supplementary Data S3**. Annotation of rRNA gene clusters and 5S rRNA genes in blue catfish genome.

**Supplementary Data S4**. Annotation of snRNA genes in blue catfish genome.

**Supplementary Data S5**. Annotation of snoRNA genes in blue catfish genome.

**Supplementary Data S6**. Annotation of miRNA genes in blue catfish genome.

**Supplementary Data S7**. List of genes of significant expansion gene family in blue catfish and their annotation from orthoDB and eggNOG-mapper.

giac070_GIGA-D-22-00096_Original_Submission

giac070_Response_to_Reviewer_Comments_Original_Submission

giac070_Reviewer_1_Report_Original_Submission Mikhail Ozerov -- 5/23/2022 Reviewed

giac070_Reviewer_2_Report_Original_Submission Jie Mei -- 5/23/2022 Reviewed

giac070_Reviewer_2_Report_Revision_1 Jie Mei -- 6/2/2022 Reviewed

giac070_Supplemental_Files

## Funding

This project is supported by the USDA National Institute of Food and Agriculture Hatch project 1018100 and an Alabama Agriculture Experiment Station (AAES) Agriculture Research Enhancement, Exploration, and Development (AgR-SEED) award. X.W. is supported by the National Science Foundation EPSCoR RII Track-4 award (OIA1928770) and a laboratory startup fund from Auburn University College Veterinary Medicine. H.W. is supported by the Auburn University Presidential Graduate Research Fellowship, College of Veterinary Medicine Dean's Fellowship, and the China Scholarship Council.

## Conflict of Interest

The authors declare no competing interests.

## Abbreviations

BLAST: Basic Local Alignment Search Tool; bp: base pair; BUSCO: Benchmarking Universal Single-Copy Orthologs; CCS: circular consensus sequencing; DNA: deoxyribonucleic acid; EM-seq: Enzymatic Methyl-Seq; ESC: Enteric Septicemia of Catfish; EST: Expressed sequence tag; GATK: Genome Analysis Tool Kit; Gb: gigabase pairs; GBS: Genotyping-by-sequencing; gDNA: genomic DNA; GEO: Gene Expression Omnibus; GWAS: Genome-Wide Association Study; HiFi: high fidelity; HMW: High molecular weight; IACUC: Institutional Animal Care and Use Committee; Kb: kilobase pairs; LINE: Long Interspersed Nuclear Element; MAS: Marker-Assisted Selection; Mb: megabase pairs; miRNA: microRNA; ML: maximum likelihood ; MT: mitochondria; NCBI: National Center for Biotechnology Information; PacBio: Pacific Biosciences; PCR: polymerase chain reaction; PL: penalized likelihood; QTL: Quantitative trait locus; RNA: Ribonucleic acid; RNA-seq: RNA sequencing; rRNA: Ribosomal RNA; snoRNA: Small nucleolar RNA; SNP: single-nucleotide polymorphism; snRNA: Small nuclear RNA; TRIP: Telomeric Repeats Identification Pipeline; TRMs: telomeric repeat motifs; tRNA: Transfer RNA; UCSC: University of California, Santa Cruz; VCF: Variant Call Format; WGD: whole-genome duplication.
