# Supplementary material for: Chromosome-level assembly and annotation of the blue catfish Ictalurus furcatus, an aquaculture species for hybrid catfish reproduction, epigenetics, and heterosis studies
Source: Gigascience. 2022 Jul 9;11:giac070. doi: 10.1093/gigascience/giac070 (PMC9270728; doi:10.1093/gigascience/giac070)
Supplement: giac070_GIGA-D-22-00096_Original_Submission [file giac070_giga-d-22-00096_original_submission.pdf]

GigaScience

Chromosome-level assembly and annotation of the blue catfish Ictalurus furcatus, an aquaculture species for hybrid catfish reproduction, epigenetics, and heterosis studies

--Manuscript Draft--

**Manuscript Number:**	GIGA-D-22-00096
**Full Title:**	Chromosome-level assembly and annotation of the blue catfish Ictalurus furcatus, an aquaculture species for hybrid catfish reproduction, epigenetics, and heterosis studies
**Article Type:**	Research
**Funding Information:**	
**Abstract:**	Background<br><br>The blue catfish is of great value in aquaculture and recreational fisheries. The F 1 hybrids of female channel catfish (Ictalurus punctatus) × male blue catfish (I. furcatus) have been the primary driver of US catfish production in recent years because of superior growth, survival, and carcass yield. The channel-blue hybrid also provides an excellent model to investigate molecular mechanisms of environment-dependent heterosis. However, transcriptome and methylome studies suffered from low alignment rates to the channel catfish genome due to divergence, and the genome resources for blue catfish are not publicly available.<br><br>Results<br><br>The blue catfish genome assembly is 841.86 Mbp in length with excellent continuity (8.6 Mbp contig N50, 28.2 Mbp scaffold N50) and completeness (98.6% BUSCO). A total of 30,971 protein-coding genes were predicted, of which 21,781 were supported by RNA-seq evidence. Phylogenomic analyses revealed that it diverged from channel catfish approximately 9 million years ago with 15.7 million fixed nucleotide differences. The within-species SNP density is 0.32% between the most agriculturally important blue catfish strains (D&B and Rio Grande). Gene family analysis discovered significant expansion of immune-related families in the blue catfish lineage, which may contribute to disease resistance in blue catfish.<br><br>Conclusions<br><br>We reported the first high-quality, chromosome-level assembly of the blue catfish genome, which provides the necessary genomic tool kit for transcriptome and methylome analysis, SNP discovery and marker-assisted selection, gene editing and genome engineering, as well as reproductive enhancement of the blue catfish and hybrid catfish.
**Corresponding Author:**	Xu Wang<br>Auburn University College of Veterinary Medicine<br>Auburn, AL UNITED STATES
**Corresponding Author Secondary Information:**	
**Corresponding Author's Institution:**	Auburn University College of Veterinary Medicine
**Corresponding Author's Secondary Institution:**	
**First Author:**	Haolong Wang
**First Author Secondary Information:**	
**Order of Authors:**	Haolong Wang
	Baofeng Su
	Ian A.E. Butts
	Rex A. Dunham

Powered by Editorial Manager® and ProduXion Manager® from Aries Systems Corporation

	Xu Wang
**Order of Authors Secondary Information:**	
**Additional Information:**	
**Question**	**Response**
Are you submitting this manuscript to a special series or article collection?	No
**Experimental design and statistics**<br><br>Full details of the experimental design and statistical methods used should be given in the Methods section, as detailed in our Minimum Standards Reporting Checklist. Information essential to interpreting the data presented should be made available in the figure legends.<br><br>Have you included all the information requested in your manuscript?	Yes
**Resources**<br><br>A description of all resources used, including antibodies, cell lines, animals and software tools, with enough information to allow them to be uniquely identified, should be included in the Methods section. Authors are strongly encouraged to cite Research Resource Identifiers (RRIDs) for antibodies, model organisms and tools, where possible.<br><br>Have you included the information requested as detailed in our Minimum Standards Reporting Checklist?	Yes
**Availability of data and materials**<br><br>All datasets and code on which the conclusions of the paper rely must be either included in your submission or deposited in publicly available repositories (where available and ethically appropriate), referencing such data using a unique identifier in the references and in	Yes

the “Availability of Data and Materials” section of your manuscript.<br><br>Have you have met the above requirement as detailed in our Minimum Standards Reporting Checklist?	

Powered by Editorial Manager® and ProduXion Manager® from Aries Systems Corporation

Manuscript
Click here to access/download;Manuscript;Blue_catfish_genome_GigaScienc

Chromosome-level assembly and annotation of the blue catfish *Ictalurus furcatus*, an aquaculture species for hybrid catfish reproduction, epigenetics, and heterosis studies

Haolong Wang[1,2], Baofeng Su[2,3], Ian A.E. Butts[2,3], Rex A. Dunham[2,3], and Xu Wang[1,2,4†]

[1]*Department of Pathobiology, College of Veterinary Medicine, Auburn University, Auburn, AL 36849*
[2]*Alabama Agricultural Experiment Station, Auburn, AL 36849*
[3]*School of Fisheries, Aquaculture and Aquatic Sciences, Auburn University, Auburn, AL 36849*
[4]*HudsonAlpha Institute for Biotechnology, Huntsville, AL 35806*

[†]corresponding author:
Xu Wang
Phone: (334) 844-7511
Fax: (334) 844-2618
E-mail: xzw0070@auburn.edu
ORCID: 0000-0002-7594-5004

Co-author E-mail addresses: H.W., hzw0088@auburn.edu; B.S., BZS0014@auburn.edu; I.A.E.B., iab0007@auburn.edu; R.A.D., dunhara@auburn.edu.

Running title: Genome assembly of blue catfish

Keywords: blue catfish, chromosomal assembly, channel catfish, heterosis, epigenetics, linked-reads technology, PacBio sequencing, D&B, Rio Grande

Abstract

Background

The blue catfish is of great value in aquaculture and recreational fisheries. The F_1 hybrids of female channel catfish (*Ictalurus punctatus*) × male blue catfish (*I. furcatus*) have been the primary driver of US catfish production in recent years because of superior growth, survival, and carcass yield. The channel-blue hybrid also provides an excellent model to investigate molecular mechanisms of environment-dependent heterosis. However, transcriptome and methylome studies suffered from low alignment rates to the channel catfish genome due to divergence, and the genome resources for blue catfish are not publicly available.

Results

The blue catfish genome assembly is 841.86 Mbp in length with excellent continuity (8.6 Mbp contig N50, 28.2 Mbp scaffold N50) and completeness (98.6% BUSCO). A total of 30,971 protein-coding genes were predicted, of which 21,781 were supported by RNA-seq evidence. Phylogenomic analyses revealed that it diverged from channel catfish approximately 9 million years ago with 15.7 million fixed nucleotide differences. The within-species SNP density is 0.32% between the most agriculturally important blue catfish strains (D&B and Rio Grande). Gene family analysis discovered significant expansion of immune-related families in the blue catfish lineage, which may contribute to disease resistance in blue catfish.

Conclusions

We reported the first high-quality, chromosome-level assembly of the blue catfish genome, which provides the necessary genomic tool kit for transcriptome and methylome analysis, SNP discovery and marker-assisted selection, gene editing and genome engineering, as well as reproductive enhancement of the blue catfish and hybrid catfish.

Introduction

Catfish is the largest segment of US aquaculture [1], and catfish farming in Mississippi, Alabama, Arkansas, and Texas accounts for 70% of total US freshwater aquaculture production. Blue catfish (*Ictalurus furcatus*) is an important aquaculture species in the US, which is native to the Mississippi River basin and along the Atlantic and Gulf coast slopes [2]. The hybrid of female channel catfish (C) and male blue catfish (B), *I. punctatus* ♀ × *I. furcatus* ♂ (C×B), constitutes more than 50% of the total harvest [3]. Additionally, blue catfish is the largest catfish species in North America and has special value for the recreational fishery due to the demand for trophy catfish for many anglers [4, 5].

US catfish production peaked in 2003 at around 300 million kg. However, it has been declining since then due to increased feed and energy cost, low catfish market price, and competition from imports, notably Asian catfish [6, 7]. Interspecific hybridization is an efficient way to recover catfish industry prosperity by producing greater genetic enhancement. The F_1 hybrid (C×B) is superior in many production traits, including faster growth rate [8-10], improved feed conversion efficiency [9, 11], more carcass yield [12], better low oxygen tolerance [13], disease resistance [14], and enhanced harvestability [15]. Collectively, these heterosis characteristics enable a commercial production rate of 13,000 kg/ha, which doubles the yield of traditional channel catfish farming [11, 16, 17]. In this context, paternal genetic contributions from blue catfish are essential for improving industry-relevant traits [18], and yet the blue catfish genome resources are not publicly available. A high-quality genome assembly of the blue catfish genome will provide the essential toolkit for the following research areas to enhance catfish breeding and advance the scientific knowledge of heterosis in fish.

In channel-blue catfish hybrids, only C×B hybrids demonstrated heterobeltiosis characteristics [15], and the superior phenotypes were only observed in pond culture but not in smaller culturing units one cubic meter or less [19]. The asymmetric and environment-dependent heterosis remains a mystery in the genetics and evolutionary biology field [20]. Transgressive genes, which are defined as genes with higher or lower expression than both parents in F_1 hybrids [21], may contribute to the superior performance in heterosis, or misregulation of gene expression resulting in hybrid incompatibility [22]. To understand the molecular mechanism of heterosis, we performed RNA-seq in blue catfish, channel catfish, and their F_1 hybrids to determine how gene regulation in hybrid catfish was altered. However, only ~60% of the blue catfish reads can be aligned to the channel catfish genome [23] due to sequence divergence, which greatly diminished the ability to investigate the gene expression differences genome-wide. Thus, a high-quality blue catfish genome assembly is necessary to improve the RNA-seq mapping rate.

Using channel catfish × blue catfish hybrid crosses and backcrosses, previous research identified major genetic loci responsible for the resistance of three economically important catfish diseases [24-27]. For certain bacterial pathogens, the blue catfish allele was the most resistant. Blue catfish were almost completely resistant to *Edwardsiella ictaluri*, the pathogen for Enteric Septicemia of Catfish [28, 29], whereas a 26% mortality in C×B hybrids [29] and up to 72.3% mortality in channel catfish were reported [28]. Blue catfish were also more resistant to the Aeromonas disease than C×B hybrids, whose mortality (32%) [30, 31] was much lower than the channel catfish (90% mortality reported in [32] and 78% in [33]) under the infection of pathogenic *Aeromonas hydrophila*. Understanding the blue catfish genome will facilitate the

selection of the disease-resistant alleles from blue catfish for superior hybrid catfish breeds. As a cost-effective approach, marker-assisted selection (MAS) has been applied to select superior breeders for traits of interest, which relies on the selection of the best representative SNPs from genome-wide association study (GWAS) peaks. Public genome assembly and annotation are already available for channel catfish [23, 34], and the catfish SNP genotyping arrays were designed primarily based on the channel catfish sequences [35, 36]. A high-quality blue catfish genome is required for the selection of ideal SNP marker sets from GWAS/QTL mapping results to ensure equal PCR amplification efficiency for both the channel catfish and blue catfish allele, and avoid SNPs located in the repeat region or paralogous sequences in either species.

In addition to transcriptomic and genomic analyses, a blue catfish reference genome will also enable epigenomic investigations, which is critical for studying the heterosis and reproduction biology of the hybrid catfish. To explain the phenotypic differences between B×C and C×B hybrids, an epigenetic component must be considered because the F_1 hybrids have identical nuclear genome configurations (29 chromosomes from the blue catfish and 29 from the channel catfish) [37]. As a key epigenetic modification [38], DNA methylation may be differentially marked in the male germline of channel catfish vs. blue catfish, affecting the global gene expression profile. The C×B hybrid catfish fry were produced using artificial reproduction techniques by mixing eggs and sperm *in vitro* [39]. Unlike other fish species, in which sperm could be easily obtained by stripping, blue catfish sperm is collected by the removal and maceration of testes, which is a lethal procedure [15, 40]. Since blue catfish males become sexually mature at 4-7 years of age [2], the paternal side (blue catfish) is the bottleneck in hybrid catfish embryo production. Cryopreservation of gametes is a solution to overcome paternal

limitations during the spawning season [41], but substantial variations in hatch rate were reported for cryopreserved blue catfish sperm samples, ranging from 0% to 82% [42-46]. Thus, a reliable method for assessing sperm quality is in urgent need within the US catfish industry, and DNA methylation is the most promising biomarker. Sperm methylation has been linked to fertility in fish [47], and it has been reported that cryopreservation can affect the DNA methylation of sperm [48]. To determine whether different storage strategies induce epigenetic lesions, DNA methylome studies have been performed in blue catfish sperm, but the proportion of mapped reads to the channel catfish reference genome is relatively low, resulting in insufficient coverage for DNA methylome analysis. Thus, a high-quality, high continuity blue catfish genome is required for epigenetic studies to enhance embryo production for hybrid catfish, and also as a genetic resource for producing better genetic types of hybrid catfish.

To fill the gap in the catfish genomic toolkit, in this study, we reported the first genome assembly of blue catfish (*I. furcatus*) using PacBio long-read sequencing. This high-quality, high-continuity assembly will allow researchers to better investigate the genomic underpinnings of production phenotypes. The annotation of the blue catfish genome makes it possible to conduct comprehensive functional genomics studies. The blue catfish genome resource will provide a solid molecular basis for investigating the mechanism of heterosis in the C×B hybrids, as well as improving the genetic potential for commercial production by genetic enhancement programs.

Materials and Methods

Fish and blood collection

Four healthy adult blue catfish (body weight: 2.8 to 3.8 kg) were obtained from the brood stock pond of Auburn University Fish Genetics Research Unit (Auburn, AL, USA), including two blue catfish (one female and one male) from the D&B strain and two blue catfish (one female and one male) from Rio Grande strain. All the fish were anesthetized using 100 mg/L buffered MS-222 (tricaine methanesulfonate, Syndel Inc., Ferndale, WA, USA), and blood samples were collected using a syringe from the caudal vasculature and immediately put into the lithium heparin-containing blood collection tubes (The Becton, Dickinson and Company, Franklin Lakes, NJ, USA). After blood collection, the four fish were temporarily reared in an indoor tank with dissolved oxygen level > 8 mg/L, water temperature 21 to 22.5 C, and pH 6.8 to 7.0, for them to recover for a few days before releasing back into the research pond. All experimental animal protocols, including animal care and tissue sample collections, were approved by the Auburn University Institutional Animal Care and Use Committee (IACUC) under PRN# 2019-3520.

Genomic DNA extraction, library preparation, and PacBio long-read sequencing

High molecular weight (HMW) genomic DNA was extracted from one female D&B, one male D&B, one female Rio Grande, and one male Rio Grande blue catfish blood samples (Table S1) using Monarch® HMW DNA Extraction Kit for Cells & Blood kit (New England BioLabs, Ipswich, MA, USA) following the manufacturer’s protocol. A total of 20 µL blood sample was used as input for each extraction. The concentration of genomic DNA was determined by a Qubit 3.0 Fluorometer instrument (Thermo Fisher Scientific, Waltham, MA, USA). The gDNA integrity and size distribution were checked by an Agilent TapeStation 4200 (Agilent

Technologies, Santa Clara, CA, USA). PacBio CCS (circular consensus sequencing) library was constructed on 10 μg female D&B blue catfish HMW genomic DNA sheared into 20 kb fragments, using SMRTbell Template Prep Kit v2 following the CCS HiFi library protocols (Pacific Biosciences, Menlo Park, CA, USA). The PacBio library was prepared and sequenced on a PacBio Sequel II System at the HudsonAlpha Genome Sequencing Center (HudsonAlpha Institute for Biotechnology, Huntsville, AL, USA).

The 10× Genomics Linked-read library preparation and Illumina short-read sequencing

Four 10× Genomics Linked-read libraries (female and male of D&B and Rio Grande strains; Table S1) were constructed on a 10× Genomics Chromium Controller (10× Genomics, Inc., San Francisco, CA, USA) using 1.1 ng HMW genomic DNA input with the Chromium Genome Reagent Kit v2 and Chromium Genome Library & Gel Bead Kit v2 (10× Genomics, Inc., San Francisco, CA, USA). Individual libraries were barcoded using the Chromium i7 Multiplex Kit (10× Genomics, Inc., San Francisco, CA, USA). Final library quality and size distribution were determined by Agilent TapeStation 4200 (Agilent Technologies, Santa Clara, CA, USA), and the concentrations were measured with Qubit 3.0 Fluorometer (Thermo Fisher Scientific, Waltham, MA, USA). The libraries were sequencing using a 2×150 bp Paired-End format on an Illumina NovoSeq 6000 sequencing at Novogene (Novogene Corporation Inc., Sacramento, CA, USA).

Genomic contig assembly

After removing the PacBio sequencing adapters and primers, the CCS HiFi reads were assembled to blue catfish contigs using hifiasm version 0.13 [49] with default parameters. *De novo* assemblies of the 10× Genomics linked-reads were performed using Supernova version

2.1.1 with default parameters [50]. The quickmerge software (version 0.3.0) [51] was used to combine the long-read and linked-read assemblies, but the outcome was the same as the PacBio contigs. Potential microbial contaminations were examined by a pipeline described previously [52], and none were identified. The contig assembly statistics were assessed using the stats.sh function in the BBMap package [53].

Chromosome assembly and polishing

Based on previous cytogenetic studies, blue catfish have the same number of chromosomes as the channel catfish (2N = 58), and the blue and channel catfish chromosomes cannot be distinguished in the karyotyping results [54]. To assemble the PacBio contigs into chromosomes, blue-channel catfish linkage information was utilized from previous linkage maps constructed using channel catfish × blue catfish crosses [55-59]. Specifically, PCR primer sequences for linkage marker from Ninwichian et al. [58] were identified in the blue catfish contigs using the UCSC In-Silico PCR tool [60] (Data S1). The contigs were ordered based on the linkage map positions, and adjacent contigs were separated by 50,000 Ns. The draft genome assembly was polished using Illumina short-reads (Table S1) for indel and error correction to generate a final high-quality assembly by Pilon (version 1.23; parameter settings: fix=all) [61]. Potential bacterial contaminations were checked using a pipeline described in our previous research [62], and no bacterial contamination was found.

Genome completeness and quality assessment

The final genome assembly statistics (Table 1) were determined by the assembly-stats script (10.5281/zenodo.594927) and the stats.sh function in BBMap [53]. Genome completeness of

blue catfish assembly was evaluated by Benchmarking Universal Single-Copy Orthologs (BUSCO) version 4.0.6 [63] and compared with the closely-related channel catfish (*Ictalurus punctatus*) reference genome [23, 64], and tra catfish (*Pangasianodon hypophthalmus*, also known as striped catfish) genome assembled recently [65-67]. Orthologs in actinopterygii_odb10 were used to compute the BUSCO scores. To determine the completeness at the chromosome termini, telomeric repeat motifs (TRM) were identified and quantified from the Illumina sequencing data using the TRIP pipeline [68], and the 6-bp vertebrate-type TRM (TTAGGG)n was identified.

To assess the quality of the blue catfish reference genome assembly, we compared blue catfish transcriptome and DNA methylome data alignment rate to channel catfish reference genome vs. this blue catfish assembly. For RNA-seq, adult liver transcriptome data from our previous research were used with NCBI GEO (Gene Expression Omnibus) databases accession number GSE186603 [19]. For DNA methylome data, DNA was extracted from three cryopreserved blue catfish sperm samples using AllPrep PowerFecal DNA/RNA Kit (Qiagen, Redwood City, CA, USA) following the manufacturer’s protocols. Pair-end EM-seq libraries were constructed using NEBNext Enzymatic Methyl-seq Kit (New England BioLabs, Ipswich, MA, USA). The quality and size distribution of libraries were determined by TapeStation 4200 (Agilent Technologies, Santa Clara, CA, USA), before they were sequenced in an Illumina NoveSeq 6000 S4 lane at Novogene (Novogene Corporation Inc., Sacramento, CA, USA). The raw EM-seq reads were checked by FastQC [69] and trimmed by Trimmomatic (version 0.36) [70]. The filtered reads were aligned to blue catfish genome assembly using the BWA aligner [71].

SNP identification between channel catfish and blue catfish, and within blue catfish between D&B and Rio Grande strains

A total of 18.6 million PacBio CCS HiFi reads generated from the blue catfish D&B strain were aligned to the channel catfish reference genome version 1.2 [23] using Minimap2 [72, 73]. *De novo* SNP calling was performed in the LongRanger pipeline v2.1.6 using UnifiedGenotyper in GATK version 3.6 [74] with "-stand_call_conf 50.0 -stand_emit_conf 10.0" parameters. SNP positions with a coverage depth of less than 10 were excluded from subsequent analysis. The 10× Genomics reads from two individuals (female and male) of the Rio Grande strain, and two individuals (female and male) of D&B strain were aligned to the repeat masked assembly of blue catfish using LongRanger software version 2.1.6 [24]. *De novo* SNP calling was performed in the LongRanger pipeline using GATK version 3.6 with the default parameters [74]. The insertion and deletion variants in VCF file were filtered out by BCFtools version 1.11 [75]. To obtain high-quality SNPs, positions with mapping quality < 250 and coverage depth < 4 were excluded from the analysis. The shared high-quality SNPs between the female and male samples were kept for the D&B and the Rio Grande strains.

Gene prediction and functional annotation

To annotate the protein-coding genes in the blue catfish genome, we exploited *ab initio*, RNA-seq based, and homology-based approaches for gene prediction in the repeat-masked assembly. Trimmed RNA-seq reads were mapped to the blue catfish genome assembly with Tophat version 2.1.1 [76], and transcript isoforms were extracted using cufflinks version 2.2.1 [77]. In addition, *de novo* transcript contig assembly was performed using Trinity version 2.4.0 [78]. The blue catfish repeat families and transcriptome assembly were fed to the MAKER annotation pipeline

version 2.31.9 [79]. Gene models were predicted using *ab initio* gene prediction algorithms with protein and transcriptome evidence by EST2GENOME and PROTEIN2GENOME procedures in MAKER. The RNA-seq GFF3 file and transcript assembly were provided as expressed sequence tags (ESTs) evidence, and annotated protein sequences of teleost species in the OrthoDB database version 9.1 were utilized as homology evidence [80]. The initially predicted gene models were used to train both the SNAP [81] and the AUGUSTUS [82, 83] gene predictors. Two additional iterations were performed to generate the final MAKER gene models. For homology-based gene prediction, high-quality channel catfish protein-coding gene models were downloaded from the Ensembl database version 99 [84], and blue catfish gene prediction was performed using Gene Model Mapper (GeMoMa) [85], based on the channel gene catfish model and BAM files of blue catfish RNA-seq alignments. Finally, the GeMoMa and MAKER gene sets were compared and merged to select the best representative gene models. To assess the quality of the gene annotation, the sequences of predicted gene models were aligned to the EST sequences from blue catfish using BLAT [86].

Annotation of non-coding RNA genes

Non-coding RNA genes were predicted by the Rfam/INFERNAL version 1.1.4 (http://eddylab.org/infernal/; accessed on 1/30/2022) using Rfam database version 14.7 [87]. The tRNA gene models were identified using tRNAscan-SE version 2.0.9 implemented in the MAKER pipeline [88]. For 28S and 18S rRNA genes, fragments gene models were excluded from the analysis.

Catfish 690K probe alignment to channel and blue catfish genomes

The widely applied catfish 690K SNP array using Affymetrix Axiom technology [35] was evaluated for SNP coverage in the blue catfish genome. The 5'- and 3'- SNP flanking sequences in the probe were aligned to the channel catfish and blue catfish genome using in the UCSC In-Silico PCR tool with 11bp tileSize and 30 bp minimum perfect match. In-Silico PCR hits less than or great than 71 bp, or with indels in them were excluded. Channel catfish and blue catfish alleles were determined for the SNP position in each probe. Density patterns of channel-blue SNPs covered by the catfish 690K array were plotted across each chromosome using the CMplot package in R [89].

Comparative genome analysis

To compare the genome assembly in blue catfish and channel catfish, the chromosome ideogram was drawn according to a previous karyotyping study [90]. The blue catfish genome sequences were compared with that of channel catfish genome sequences to identify chromosome orthology. Multiple genome alignment and visualization were performed using Mauve version 2.1.0 [91]. Unique genomic regions with high sequencing similarity and genome rearrangement events between blue catfish and channel catfish were highlighted for each chromosome pair comparison. In addition to DNA sequences, we also compared the two genomes using protein-coding genes as anchors. Homologous regions in these two genomes were identified using MCScanX [92], a Python package for synteny detection and evolutionary analysis. The inferred gene pairs and linked relationships were visualized and placed in the context of whole-genome collinearity using a genomic circle generated by Circos [93].

Repeat annotation

To compare the quality of blue catfish genome with related species genome, RepeatModeler version 2.0.1 [94] was performed to identify the repetitive elements in our blue catfish genome assembly, channel catfish (*Ictalurus punctatus*) genome assembly [64], and tra catfish (*Pangasianodon hypophthalmus*) genome [65]. The interspersed repeats sequences and low complexity DNA sequences were masked with RepeatMasker version 4.0.6 [95].

Phylogenetic analysis

To investigate the phylogenetic relationship between blue catfish and other Actinopterygii fish, ten species were selected from 52 Actinopterygii species in OrthoDB version 10.1 [96], including channel catfish (*I. punctatus*), Atlantic herring (*Clupea harengus*), zebrafish (*Danio rerio*), northern pike (*Esox lucius*), large yellow croaker (*Larimichthys crocea*), spotted gar (*Lepisosteus oculatus*), nile tilapia (*Oreochromis niloticus*), guppy (*Poecilia reticulata*), greater amberjack (*Seriola dumerili*), and pufferfish (*Takifugu rubripes*). A total of 5269 single-copy 1:1 orthologs among these species were identified. Protein sequences were aligned using MAFFT version 7.407 [97] and concatenated into one alignment for phylogenomic analysis. IQ-TREE [98] was used to estimate the best protein model for phylogenetic tree construction. A Maximum Likelihood (ML) tree was built with the best-fit model JTT+F+R3, with 1,000 bootstraps or branch support evaluation. The phylogenetic tree was annotated and visualized using FigTree version 1.4.4 [99].

Gene family expansion and contraction analyses

The gene models from the blue catfish and other 10 Actinopterygii species in the phylogenomics analysis were obtained from this assembly and OrthoDB version 10.1 [96], respectively. Protein

sequences less than ten amino acids were excluded from the analyses. Sequence alignments were performed by all-vs-all blast module using Diamond (version 2.0.0) [100], with an E-value cutoff of 1×10^{-5}. Gene family clusters were identified using OrthoMCL (version 2.0.9) [101]. The divergence time was estimated based on the phylogenetic tree and protein sequence by r8s (version 1.8.1) [102]. The PL (penalized likelihood) method and TN algorithms were used to estimate divergence time and absolute rates of substitution. Three calibration nodes were applied for estimating divergence time, including one fixed time point (*I. punctatus* & *Danio rerio*, 142 million years ago) and two constraining time points (*Poecilia reticulata* & *Oreochromis niloticus*, 88-139 million years ago and *Larimichthys crocea* & *Takifugu rubripes*, 102-127 million years ago) from the TimeTree [103]. Based on estimated divergence times and phylogenetic relationships, CAFE (version 4.2.1) [104] was used to analyze the gene family expansion and contraction. The cutoff for significantly changed gene families was *P*-value less than 0.05. The genes in significantly expanded gene families were annotated by orthology in other species from OrthoDB version 10.1 or the eggNOG-mapper [105].

Results

Genome assembly and statistics

A total of 316.9 Gb PacBio raw reads were generated from the blood DNA of a single female blue catfish (D&B strain). Genome assembly was performed using 21.3 Gb PacBio CCS HiFi reads, and a total of 563 contigs were assembled, which was much fewer than the long-read assemblies of channel catfish and tra catfish genomes (Table 1 and Table S1). The assembled contigs had an N50 of 8.59 Mb, indicating excellent contiguity. The contigs were further assembled into chromosomes based on linkage markers and linkage map information constructed using F1 backcrosses in 2016 [58]. Both species had 29 pairs of homologous chromosomes, and no karyotype differences can be detected at the cytogenetic level [54, 90]. The genetic maps and physical distances were consistent between blue catfish and channel catfish chromosomes (Figure 1A-B). On average, channel catfish had 60.0 linkage markers per chromosome, whereas blue catfish assembly had 34.8 markers, with 32.8 overlapped in both species (Figure 1C and Data S1). This was because the PCR primers were designed based on channel catfish sequences when the blue catfish genome was not available. Given that the number of markers was much larger than the number of scaffolds per chromosome (n = 11), the channel-blue linkage markers were sufficient for the chromosomal assembly. The final assembly consisted of 29 chromosomes, a circularized mitochondrial genome, and 241 unplaced contigs. The final genome size was 841,864,377 bp, with a scaffold N50 of 28.24 Mb (Table 1).

Assessment of genome completeness

The completeness of blue catfish genome assembly was assessed using the BUSCO tool (see Materials and Methods). The BUSCO completeness score was 98.6%, which was slightly higher

than the channel catfish (97.1%) and tra catfish (93.3%; see Table 1). No duplicated or fragmented BUSCO genes were identified in the blue catfish genome, suggesting a highly complete reference assembly free of redundancy and contamination (Table 1). We examined the termini of the assembled chromosomes and discovered that 43 of the 58 telomeres start or end with the vertebrate-type telomeric repeat motif TTAGGG, indicating telomere-to-telomere continuity for the majority of the chromosomes (Figure 2A and Table S2). In contrast, only ten telomeric regions were present in the channel catfish assembly (Figure 2A and Table S3). Over 90% of the 10× Genomics reads from the female D&B library can be uniquely aligned to the blue catfish assembly, and 99.5% of the blue catfish genome was covered by the Illumina reads (Table S1).

Protein-coding gene annotations

A total of 17,945 gene models were annotated by SNAP, and 10,591 genes were predicted by AUGUSTUS in the MAKER pipeline (see Materials and Methods). Homology-based GeMoMa algorithm identified 20,460 protein-coding genes based on channel catfish gene models in Ensembl version 99. These gene models were merged based on BLAT results to the blue catfish genome assembly, resulting in 33,677 predicted protein-coding genes. Among these gene models, 30,971 (92.0%) are complete with a start codon and a stop codon. The average coding region length was 1,195 bp, and the mean number of coding exons per gene model was 7.2. There were a total of 21,781 genes with RNA-seq reads aligned to the gene region (Table S4), and 21,330 gene models were supported by blue catfish EST sequences.

Annotation of non-coding RNA genes

A total of 6,192 tRNAs were identified using tRNA-scan (Table 2 and Data S2). Among them, 1,712 decode the twenty standard amino acids, eight decode selenocysteine (TCA), and ten are predicted suppressor tRNAs. The remaining ones are predicted pseudogenes. A total of 55 complete clusters of 18S-5.8S-28S rRNA gene clustered were predicted (Table 2 and Data S3), and which were located on chromosome 4 and four unplaced scaffolds (chrUn008, chrUn028, chrUn032, chrUn034). The blue catfish 28S rRNA gene has a 99.2% sequence identity with the channel catfish gene on chromosome 24, and a 92.4% identity with Tra catfish (XR_004577711). The blue catfish 18S rRNA gene has a 98.8% sequence identity with channel catfish (AF021880), and a 98.3% identity with Tra catfish (XR_004577708). The blue catfish 5.8S rRNA genes have a 100% sequence identity with channel catfish (GQ465242), and a 99.4% identity with Tra catfish (XR_003402644). A total of 12,448 copies of predicted 5S rRNA genes were identified (Table 2), 95.6% of which were located in a 4 Mbp cluster on chromosome 14 subtelomeric region between positions 29,127,406 bp and 33,137,707 bp (Data S3). Spliceosome-related small nuclear RNAs (snRNAs) include U1, U2, U4, U5, U6, and other members (Data S4). Altogether, there were 601 predicted copies of snRNAs (Table 2), and they were organized in major clusters in the blue catfish genome (U1 on chr1, U2 on chr19, U4 on chr14, U5 on chr19, and U6 on chr22). Small nucleolar RNAs (snoRNAs) are responsible for nucleotide modifications in rRNAs. A total of 135 C/D-box snoRNAs and 72 H/ACA-box snoRNAs were predicted in the blue catfish genome (Table 2 and Data S5). A total of 2,079 pre-miRNA genes were predicted in the blue catfish genome (Table 2 and Data S6), which encode 105 putative miRNAs.

Ancient whole genome duplication in blue catfish

Teleost species underwent a fish-specific ancient whole-genome duplication (WGD) event during evolution [106]. To investigate this, we performed synteny analysis within the blue catfish genome. A significant amount of reminiscent paralogy was found in a few chromosome pairs (chr4-chr5, chr9-chr25, and chr15-chr21), as well as local regions among other chromosomes (Figure S1).

Comparative genomics between the blue catfish and the channel catfish

Comparative genomic analysis was performed using the whole-genome alignment tool Mauve (see Materials and Methods), and blue-channel pairwise chromosomal alignment revealed large syntenic blocks between homologous chromosomes (Figure 2B-C and Figure S2). For example, chromosome 1 only had four inversion events involving small regions near the centromere and telomere (Figure 2B). Local genome expansions and contractions were observed, but they only accounted for a small fraction of the genome. Gene model and gene order based comparisons of the two genomes were also performed (Figure 3), and a high level of synteny between the corresponding chromosomes was also detected, with a few minor genome rearrangements (Figure 3).

Divergence between D&B strain and Rio Grande blue catfish strains

Through *de novo* SNP calling using GATK, we identified a total of 1,433,465 SNP positions between the D&B and the Rio Grande strains. Among these, 607,059 were fixed differences between D&B and Rio Grande, and 826,406 were segregating within the Rio Grande strain. We estimated that the intraspecific SNP density between these two strains was 0.0032. The assembled and circularized D&B mitochondrial (MT) genome is 16,499 bp in length, which is

the same length as a previously assembled blue catfish MT genome (NCBI GenBank accession number NC_028151). We identified one fixed nucleotide difference between our D&B MT genome assembly and NC_028151 (C12307T), which is a synonymous substitution at the third position in a codon encoding Phe. We assembled the first MT genome in the Rio Grande strain, and the circularized genome size is also 16,499 bp. There are 53 substitutions between D&B and Rio Grande (36 in protein-coding genes, 2 in rRNA, and 2 in tRNA genes), resulting in a sequence divergence of 0.0032 in the entire genome and 0.0026 in the genic region (Table S5).

SNP annotation and channel genome-based pseudo blue catfish genome

The interspecific sequence differences between channel catfish and blue catfish were previously estimated to be 13 to15 SNPs per Kb based on EST data [107]. In this study with the blue catfish genome assembly and resequencing data of two important aquaculture strains (D&B and Rio Grande), *de novo* SNP calling revealed a total of 15,685,661 fixed differences between the blue catfish and the channel catfish (depth > 10, PacBio alignment quality > 150), which is 18.7 SNPs per Kb. This genome-wide estimation is higher than in the more conserved EST sequences. Regions of lower than average SNP density were often located at the chromosome ends (Figure 4A). The blue catfish and channel catfish MT genome have a total of 1,041 fixed differences in the coding region, which is 9.1% coding sequence divergence (Table S6). Among these substitutions, only 79 (0.069% in the coding region) were non-synonymous, suggesting strong purifying selection.

Evaluation of the catfish 690 K SNP array on blue catfish genome

The 690K catfish SNP array was designed in 2017 [35], incorporating SNP information from the

channel catfish reference genome [23] and tissue-specific RNA-seq datasets, as well as blue catfish RNA-seq and GBS data. To validate the probes in the newly assembled blue catfish genome, the probes were aligned to both channel and blue reference genomes. A total of 99.0% of 690K probes were aligned to the channel catfish genome, and 98.2% had a unique perfect hit without any indels or mismatches in the flanking probe sequences (Table 3), suggesting excellent accuracy. When these probes were mapped to the blue catfish genome, 29.3% were mapped, and only 23.9% of probes had unique perfect hits (Table 3). Among these probes, only 76,399 (11.0% of 690K) overlapped with a channel-blue catfish SNP position (Table 3). These interspecific informative SNP probes were sparsely distributed across the chromosomes (Figure 4B), with < 5 SNP per 100 Kb for 20% of the genome.

Improvement of transcriptome and DNA methylome alignments to the newly assembled blue catfish genome

For the blue catfish liver RNA-seq data generated in our previous research, only 65.4% of RNA-seq reads can be aligned to the channel catfish reference genome [64] due to the reference bias (Table S4). We constructed a pseudogenome for blue catfish based on the channel reference genome IpCoco 1.2, by replacing the channel alleles with blue alleles at the 15,685,661 channel-blue SNP positions we identified. The average RNA-seq alignment rate improved to 71.8%, which had a 9.8% increase in uniquely mapped reads. However, the pseudogenome mapping rate is still relatively low, which may result in poor coverage for some blue catfish genes (Table S4). When the newly assembled blue catfish genome was used as the reference, the average percentage of uniquely mapped reads increased to 85.1% (a 30% increase compared to using the channel reference), which maximized the read coverage for expressed genes (Table S4). Blue

catfish sperm DNA methylome sequence mapping rate was also significantly improved using the blue catfish genome assembly, with ~80% of the EM-seq reads uniquely mapped to the blue catfish genome.

Repeat annotation

Repetitive sequence annotation identified that 47% (395.5 Mb) of the blue catfish genome belongs to repetitive regions (Table 4), which was in between the channel catfish (51%) [108] and Tra catfish (40%). Among the known repeats in blue catfish, the DNA transposon superfamily *pogo* was the most abundant, accounting for 43% of all known repeats, or 8.2% of the entire genome (Table 4). *Pogo* has the bacteria *IS630* transposase, and was repeatedly domesticated in vertebrates, including fish [109]. Channel catfish genome also had 7.8% of *Pogo* repeats, but it is less abundant in Tra catfish (4.7%). The following classes of repeat accounted for more than 1% of the blue catfish genome: LINEs (3.0%), Gypsy/DIRS1 elements (2.3%), L2/CR1/Rex clade (1.9%), and simple repeats (3.3%).

Phylogenomic analysis with teleost genomes

To understand the phylogenetic relationship of *I. furcatus* with other Actinopterygii species, we used 4,698 single-copy 1:1 orthologs to construct a phylogenetic tree of eleven species, including blue catfish (*I. furcatus*), channel catfish (*I. punctatus*), Atlantic herring (*Clupea harengus*), zebrafish (*Danio rerio*), northern pike (*Esox lucius*), large yellow croaker (*Larimichthys crocea*), spotted gar (*Lepisosteus oculatus*), Nile tilapia (*Oreochromis niloticus*), guppy (*Poecilia reticulata*), greater amberjack (*Seriola dumerili*), and pufferfish (*Takifugu rubripes*). The phylogenomic analysis provides highly supported internal branches with 100 bootstrap values.

The blue catfish fall into the same clade with channel catfish, which is consistent with the known phylogenetic relationships.

Gene family expansion and contraction in blue catfish genome

To reveal the gene family evolution among blue catfish and other ten fish species, divergence times and gene family expansion/contraction were determined for each species (see Materials and Methods). Phylogenomic analysis indicated that the divergence of blue catfish (*I. furcatus*) and channel catfish (*I. punctatus*) occurred approximately 9 million years ago, according to 4,698 single-copy ortholog genes in these 11 species (Figure 6). A total of 25,027 gene families were examined for expansion/contraction analysis. In blue catfish, 331 expanded gene families and 1,963 contracted families were identified (Figure 6 and Table S7). Among them, 50 families underwent rapid expansion (Table S8), containing 263 blue catfish genes (Data S7). These expanded gene families include immunoglobulin domain proteins (9 families and 44 genes) and gene families involved in immune functions (9 families and 59 genes; see Table S8). Seven families of transposable element genes (n = 55), such as reverse transcriptases and transposases, are the second-largest category of rapidly expanded families.

Discussion

A chromosome-level genome assembly of an aquaculturally important species

Currently, both industry and academic researchers are focusing on improving catfish production efficiency. As the paternal species for hybrid catfish, blue catfish is undoubtedly important for the genetic and reproductive enhancement of hybrid catfish production, but a reference genome is not publicly available yet. To fill in this gap, we assembled and reported the first blue catfish genome using a combination of Pacific Biosciences CCS (circular consensus sequence) and 10× Genomics linked-read technologies. The final assembly is chromosome-level, telomere-to-telomere for many chromosomes, with scaffold N50 of 28.2 Mbp and contig N50 of 8.6 Mbp. The BUSCO completeness score is 98.6%, with no duplicated or fragmented BUSCOs. The assembly statistics data indicates our assembly is excellent in terms of both genome continuity and completeness.

High-quality blue catfish genome and well-annotated gene catalog provide the necessary reference genome for genotyping, genome editing, transcriptome, and methylome analyses.

Previously, the channel catfish genome was used as the reference for blue catfish sperm RNA-seq and DNA methylome analyses, which had significantly lower mapping rates, and many critical blue catfish genes were not covered due to higher divergence. Our novel blue catfish assembly dramatically improved the sequencing alignment rate for RNA-seq, Methyl-seq, and genome resequencing data in blue catfish. Using computational prediction and RNA-seq evidence-based approaches, we identified a total of 33,686 protein-coding genes. Of these, over twenty thousand genes are with RNA-seq and blue catfish EST support. The number of gene models is comparable to the channel catfish annotation [23], which is a well-annotated genome.

Genetic enhancement of catfish production has been underway using MAS, and further improvement could be achieved through targeted gene editing in the hybrid catfish background, which has gained great momentum in aquaculture. In channel catfish, the CRISPR-Cas system has been successfully applied to understand genetic regulation and mechanisms of economically important traits and attributes, including the chemokine C-C motif ligand 33 in babel regeneration [110], myostatin in body weight regulation [111], an exogenous alligator cathelicidin gene in pathogen defense [112], and interleukin 1 receptor and lectin genes for improved immune response and disease resistance [113]. The availability of high-quality channel catfish reference genome provided essential information for guide RNA design, homologous arms, and non-coding region screening. In hybrid catfish, many superior alleles came from the blue catfish. Without the blue catfish reference genome sequence, it is not possible to design guide RNAs and predict potential off-target effects for genome editing to improve economically important traits. Furthermore, one of the major limiting factors in hybrid catfish reproduction is the lack of technical standards for sperm collection and quality assessment. DNA methylation is the most reliable indicator of sperm quality and ensuing offspring performance. Research is underway to elucidate the utility of DNA methylation status for the prediction of hatch rate and inform the industry of best practices. The availability of this high-quality blue catfish genome is critical to enable the paternal side genetic and epigenetic enhancement of hybrid catfish genetic types.

Blue catfish genome assembly facilitates the understanding of environment-dependent heterosis, an intriguing phenomenon in hybrid catfish

The phenomenon of heterosis was first reported by Charles Darwin [114], and has been under extensive study by evolutionary biologists since then. Hybrids of blue catfish sire and channel catfish dam are superior in many disease resistance and productive traits. Intriguingly, this heterosis was only observed for growth in pond culture. In smaller culturing units, such as tanks and aquaria, channel catfish are superior to blue catfish or hybrid catfish [19], which was the first report of environment-dependent heterosis in any vertebrate species. The molecular mechanism of this unique environment-dependent heterosis is worth investigating. However, without a blue catfish genome and a genome-wide SNP dataset, it is impossible to accurately profile allele-specific gene expression and DNA methylation in hybrids. In this study, we identified over 15 million SNPs between the channel catfish and the blue catfish, and the number of SNPs in the transcripts and regulatory regions is sufficient for genome-wide profiling of parent-of-origin expression [115]. The availability of blue catfish reference genome will help elucidate the regulatory and epigenetic mechanism of environment-dependent heterosis in hybrid catfish.

The blue catfish genome provides new insight into blue-channel divergence and Siluriformes evolution

In the present study, we estimated that blue catfish diverged from channel catfish approximately 9 million years ago, based on 1:1 single-copy orthologs. The estimate is significantly more recent than a previous prediction (16.6 million years) based on the divergence of the cytochrome b gene alone [108], which might be an overestimation due to the accelerated substitution rate in the mitochondrial genome. The comparative genomic analysis confirmed that the 29 chromosomes are mainly synteny between the channel catfish and blue catfish with a few local rearrangements. Phylogenomic analysis of reference fish genomes in OrthoDB revealed that zebrafish was the

closest outgroup species to catfish species, which is congruent with the known phylogeny of ray-finned fishes [116] and is also consistent with previous findings that the most closely related model fish species is zebrafish [117]. Many genomes are available in Siluriformes, including the Asian redtail catfish (*Hemibagrus wyckioides*) [118] and yellow catfish (*Tachysurus fulvidraco*) [119] in the family of Bagridae, giant devil catfish (*Bagarius yarrelli*) in Sisoridae [120], 12 Pangasiidae species [65-67], three Clariidae species [121-123], as well as channel catfish (*Ictalurus punctatus*) and black bullhead catfish (*Ameiurus melas*, GCA_012411365) in the family of Ictaluridae. Our blue catfish genome will provide an invaluable resource to investigate molecular phylogeny and comparative analysis in Siluriformes.

Comparative analysis of channel and blue catfish genomes reveals subchromosomal level differences and the expansion of immune function related genes in blue catfish

Although blue catfish and channel catfish have morphologically indistinguishable chromosomes with essentially identical Giemsa banding patterns [54, 90], in this study, we identified 15 million SNPs, and demonstrated structural rearrangement events between the two species, as well as differences in gene and repeat content. These genetic differences may explain the myriad of species-specific traits related to growth, disease resistance, body conformation and coloration, the incidence of albino fish, behavior, seinablity, age and size of maturity, as well as responses to hormone-induced spawning.

Interestingly, we identified blue catfish lineage-specific gene family expansions. It is not surprising that transposases and reverse transcriptases are among the rapidly expanding family because of the active TE (transposable elements) turnover. Genes with immune-related functions

account for 40% of the known expanded families, including T-cell receptor delta, lectin, complement control proteins, glucocorticoid receptor, chemokine interleukin 8, CD225/Dispanin, and others. This is consistent with our previous findings that the blue catfish had the highest immune activity compared to the channel and hybrid catfish at 10-month of age [19], and the gene family expansion may contribute to the superior phenotypes of lysozyme and alternative complement activities in blue catfish. Further understanding of the evolution of immune-related genes will provide valuable information for the genetic enhancement of hybrid catfish in pathogenic disease resistance.

A third-generation catfish SNP array is necessary for improved resolution in future GWAS analysis for disease resistance and growth improvement

Pathogenic infection disease is the number one cause of catfish production loss. Paternal genetic contributions from blue catfish are essential for improving industry-relevant traits [18], and F1 hybrid catfish can reduce loss from 40% to 20% by carrying disease-resistant alleles from the blue catfish genome. Our research team along with other researchers have identified the genetic loci responsible for the resistance of three major catfish diseases, ESC, columnaris, and Aeromonas diseases [24-27], and in most cases, the blue catfish allele is the resistance allele. Based on the knowledge learned from these GWAS and QTL mapping studies, marker-assisted selection (MAS) using F2 and F1 backcrosses would be an ideal and effective approach to select superior breeders for traits of interest. Choosing the best representative blue-channel SNPs from the GWAS peaks requires a high-quality blue catfish genome due to the following reasons. First, equal PCR amplification efficiency or probe affinity is ideal for the SNP typing assays, and sequence information is needed from both channel and blue genomes for proper primer design.

Second, the presence of paralogous sequences will result in spurious SNP calls, and the blue catfish genome is needed to exclude these positions. Last but not least, historical whole-genome duplications in fish genomes further complicate accurate SNP genotyping [124]. Our report in this study meets this urgent need for a high-quality blue catfish genome.

Additional genetic enhancement of the hybrid catfish is essential for better profitability and sustainability. To further improve disease resistance through genomic techniques, we must understand the blue catfish genome in single-base pair resolution. With the new blue catfish assembly, we identified 15 million fixed differences between blue and channel catfish, with a density of 18.7 SNPs per Kb. This is higher than the previous estimation from the blue EST database (13 to 15 SNPs per Kb) [107], which is exactly expected due to higher conservation in transcribed genes. Our study provided a correct genome-wide estimation of the blue-channel divergence, and the necessary information for SNP typing primer/probe design. SNP arrays are the widely used approach for cost-effective genotyping experiments. The first generation 250K SNP array was designed based on channel catfish sequences [36], and the second generation 690K SNP array was designed based on channel catfish reference genome plus blue catfish EST sequences [35]. Because the 690K array is more comprehensive and replaced the previous generation, we evaluated the probes of the 690K array using the new blue catfish genome. Only 24% of the probes had unique perfect matches to the blue catfish genome, and more than half of them were targeting invariant positions between the channel catfish and blue catfish genome (some probes were designed to target segregating SNPs within channel catfish [35]). Overall, only 11% of the 690K SNP array probes are informative for channel-blue SNPs, with fairly uneven distribution in the genome. Therefore, we think a third-generation catfish genotyping

array is needed to fully leverage the channel-blue SNPs for MAS and GWAS studies with improved informativeness, genome resolution, and statistical power, which would be versatile for both initial genome screen and fine mapping purposes. The new SNP array could benefit from a gene region enriched design based on the blue catfish gene models predicted in this study, which may help identify the causal SNPs in coding regions for specific traits of interest.

SNP analysis between the D&B and Rio Grande strains provides the genetic toolkit for blue catfish and hybrid catfish breed enhancement

The D&B blue catfish strain was widely used in commercial aquaculture, which was obtained originally from the Arkansas and Mississippi River [125]. D&B was selected for PacBio sequencing because it has been considered the reference strain in farming practices since 2010 [126]. Because the growth and disease resistance advantages of the hybrid catfish are unidirectional (channel catfish female × blue catfish male), researchers must focus on the blue catfish for the reproductive enhancement of the male side. In this context, another blue catfish strain, Rio Grande, which originated from the Rio Grande native to Texas [125], was developed by Auburn University [7, 127, 128]. Recent breed development discovered that the Rio Grande strain is superior to D&B in terms of maturation rate, testis size, and the quality/quantity of sperm production [126, 129, 130]. Rio Grande males reach sexual maturity at age 3-4, which is significantly earlier than D&B. Due to these superior traits in reproduction, Rio Grande has been recently included in the USDA-ARS Catfish Genetic Enhancement Program, and USDA is releasing them to stakeholders. In addition, blue catfish strains were shown to have significant variability in disease resistance and mortality [131]. Big differences also exist in growth, body coloration (Rio Grande is the only catfish strain with spots), and seinability. However, the

genetic diversity between D&B and Rio Grande was not investigated. To understand the genetic architecture of blue catfish strains, we sequenced the genomes of female and male Rio Grande broodstock, and performed genomic analysis. A total of 1.4 million SNPs were identified in the nuclear genome (1.7 per Kb), including 600K fixed differences between the two strains and 826K SNPs segregating within Rio Grande. The level of genetic diversity is sufficient for the genetic enhancement of male gamete production and potential disease resistance. In contrast, the mitochondrial genome divergence is fairly low (2.6 SNP per Kb in the genic region), with only 5/36 non-synonymous substitution between D&B and Rio Grande, suggesting potential purifying selection. The nuclear and mitochondrial SNPs provide informative genetic markers to genotype the D&B and the Rio Grande strains. The genetic background differences between D&B and Rio Grande may explain the variations in sperm quality, male side reproductive traits, growth, body color, and pathogenic disease resistance.

Data Availability Statement

Supplemental Data is available at github.com/XuWangLab/DB_genome_assembly. The draft genome assembly of *Ictalurus furcatus* has been deposited at NCBI under Assembly accession number JAJOLW000000000 and project ID PRJNA785621. The mitochondrial genome of the blue catfish D&B strain is submitted to NCBI GenBank under accession number ON022108. The mitochondrial genome of the blue catfish Rio Grande strain is submitted to NCBI GenBank under accession number ON022107.

Acknowledgments

This project is supported by the USDA National Institute of Food and Agriculture Hatch project 1018100 and an Alabama Agriculture Experiment Station (AAES) Agriculture Research Enhancement, Exploration, and Development (AgR-SEED) award. X.W. is supported by the National Science Foundation EPSCoR RII Track-4 award (OIA1928770), and a laboratory start-up fund from Auburn University College Veterinary Medicine. H.W. is supported by the Auburn University Presidential Graduate Research Fellowship and College of Veterinary Medicine Dean's Fellowship. We thank the HudsonAlpha Genome Sequencing Center for assistance with the PacBio sequencing. We acknowledge the Auburn University Easley Cluster for support of this work.

References

1. Engle CR, Kumar G and van Senten J. Cost drivers and profitability of US pond, raceway, and RAS aquaculture. Journal of the World Aquaculture Society. 2020;51 4:847-73.
2. Graham K. A review of the biology and management of blue catfish. In: *Catfish 2000: proceedings of the international ictalurid symposium American Fisheries Society, Symposium* 1999, pp.37-49.
3. Torrans L and Ott B. Effect of grading fingerling hybrid catfish (♀ channel catfish×♂ blue catfish) on growth, production, feed conversion, and food fish size distribution. North American Journal of Aquaculture. 2018;80 2:187-92.
4. Hyman AA, DiCenzo VJ and Murphy BR. Muddling management: heterogeneity of blue catfish anglers. Lake and Reservoir Management. 2017;33 1:23-31.
5. Bunch AJ, Greenlee RS and Brittle EM. Blue catfish density and biomass in a tidal tributary in coastal Virginia. Northeastern Naturalist. 2018;25 2:333-40.
6. Hanson T and Sites D. 2010 Catfish Database. Information Report 2011–01. 2011.
7. Dunham RA, Ramboux AC and Perera DA. Effect of strain on the growth, survival and sexual dimorphism of channel× blue catfish hybrids grown in earthen ponds. Aquaculture. 2014;420:S20-S4.
8. Giudice JJ. Growth of a blue x channel catfish hybrid as compared to its parent species. The Progressive Fish-Culturist. 1966;28 3:142-5.
9. Dunham RA, Umali GM, Beam R, Kristanto AH and Trask M. Comparison of production traits of NWAC103 channel catfish, NWAC103 channel catfish× blue catfish hybrids, Kansas Select 21 channel catfish, and blue catfish grown at commercial densities and exposed to natural bacterial epizootics. North American Journal of Aquaculture. 2008;70 1:98-106.
10. Dunham RA and Brummett RE. Response of two generations of selection to increased body weight in channel catfish, Ictalurus punctatus, compared to hybridization with blue catfish, I. furcatus, males. Journal of Applied Aquaculture. 1999;9 3:37-45.
11. Brown TW, Chappell JA and Boyd CE. A commercial-scale, in-pond raceway system for Ictalurid catfish production. Aquacultural engineering. 2011;44 3:72-9.
12. Bosworth BG. Effects of winter feeding on growth, body composition, and processing traits of co-cultured Blue Catfish, Channel Catfish, and Channel Catfish× Blue Catfish hybrids. North American Journal of Aquaculture. 2012;74 4:553-9.
13. Dunham RA, Smitherman RO and Webber C. Relative tolerance of channel x blue hybrid and channel catfish to low oxygen concentrations. The Progressive Fish-Culturist. 1983;45 1:55-7.
14. Arias CR, Cai W, Peatman E and Bullard SA. Catfish hybrid Ictalurus punctatus× I. furcatus exhibits higher resistance to columnaris disease than the parental species. Diseases of aquatic organisms. 2012;100 1:77-81.
15. Dunham RA and Masser MP. Production of hybrid catfish. Southern Regional Aquaculture Center Stoneville, Mississippi, USA; 2012.
16. Bott LB, Roy LA, Hanson TR, Chappell J and Whitis GN. Research verification of production practices using intensive aeration at a hybrid catfish operation. North American Journal of Aquaculture. 2015;77 4:460-70.
17. Kumar G, Engle CR, Hanson TR, Tucker CS, Brown TW, Bott LB, et al. Economics of alternative catfish production technologies. Journal of the World Aquaculture Society. 2018;49 6:1039-57. doi:https://doi.org/10.1111/jwas.12555.

18. Myers JN, Chatakondi NG, Dunham RA and Butts IAE. Genetic architecture of early life history traits for channel catfish, Ictalurus punctatus ♀ × blue catfish, I. furcatus ♂ hybrid production. Aquaculture. 2020;514:734436. doi:https://doi.org/10.1016/j.aquaculture.2019.734436.
19. Wang H, Bruce TJ, Su B, Li S, Dunham RA and Wang X. Environment-Dependent Heterosis and Transgressive Gene Expression in Reciprocal Hybrids between the Channel Catfish Ictalurus punctatus and the Blue Catfish Ictalurus furcatus. Biology. 2022;11 1:117.
20. Govindaraju DR. An elucidation of over a century old enigma in genetics—Heterosis. PLoS biology. 2019;17 4:e3000215.
21. Banho CA, Mérel V, Oliveira TY, Carareto CM and Vieira C. Comparative transcriptomics between Drosophila mojavensis and D. arizonae reveals transgressive gene expression and underexpression of spermatogenesis-related genes in hybrid testes. Scientific reports. 2021;11 1:1-15.
22. Crow J and Pandey S. Dominance and overdominance. Genetics and exploitation of heterosis in crops: Based on the International Symposium on the genetics and exploitation of heterosis in crops organized and hosted by the CIMMYT in México City, 17-22 Aug 1997. International Maize and Wheat Improvement Center, México (México). 1999.
23. Liu Z, Liu S, Yao J, Bao L, Zhang J, Li Y, et al. The channel catfish genome sequence provides insights into the evolution of scale formation in teleosts. Nat Commun. 2016;7:11757. doi:10.1038/ncomms11757.
24. Shi H, Zhou T, Wang X, Yang Y, Wu C, Liu S, et al. Genome-wide association analysis of intra-specific QTL associated with the resistance for enteric septicemia of catfish. Molecular Genetics and Genomics. 2018;293 6:1365-78.
25. Tan S, Zhou T, Wang W, Jin Y, Wang X, Geng X, et al. GWAS analysis using interspecific backcross progenies reveals superior blue catfish alleles responsible for strong resistance against enteric septicemia of catfish. Molecular Genetics and Genomics. 2018;293 5:1107-20.
26. Geng X, Sha J, Liu S, Bao L, Zhang J, Wang R, et al. A genome-wide association study in catfish reveals the presence of functional hubs of related genes within QTLs for columnaris disease resistance. BMC genomics. 2015;16 1:1-12.
27. Zhou T, Liu S, Geng X, Jin Y, Jiang C, Bao L, et al. GWAS analysis of QTL for enteric septicemia of catfish and their involved genes suggest evolutionary conservation of a molecular mechanism of disease resistance. Molecular genetics and genomics. 2017;292 1:231-42.
28. Wolters WR and Johnson MR. Enteric Septicemia Resistance in Blue Catfish and Three Channel Catfish Strains. Journal of Aquatic Animal Health. 1994;6 4:329-34. doi:10.1577/1548-8667(1994)006<0329:ESRIBC>2.3.CO;2.
29. Wolters WR, Wise DJ and Klesius PH. Survival and Antibody Response of Channel Catfish, Blue Catfish, and Channel Catfish Female × Blue Catfish Male Hybrids after Exposure to Edwardsiella ictaluri. Journal of Aquatic Animal Health. 1996;8 3:249-54. doi:10.1577/1548-8667(1996)008<0249:SAAROC>2.3.CO;2.
30. Li C, Beck B, Su B, Terhune J and Peatman E. Early mucosal responses in blue catfish (Ictalurus furcatus) skin to Aeromonas hydrophila infection. Fish Shellfish Immunol. 2013;34 3:920-8. doi:10.1016/j.fsi.2013.01.002.
31. Zhou T, Yuan Z, Tan S, Jin Y, Yang Y, Shi H, et al. A Review of Molecular Responses of Catfish to Bacterial Diseases and Abiotic Stresses. Front Physiol. 2018;9:1113. doi:10.3389/fphys.2018.01113.
32. Zhang D, Xu D-H and Shoemaker C. Experimental induction of motile Aeromonas septicemia in channel catfish (Ictalurus punctatus) by waterborne challenge with virulent Aeromonas hydrophila. Aquaculture Reports. 2016;3:18-23. doi:https://doi.org/10.1016/j.aqrep.2015.11.003.

33. Zhang D, Moreira GS, Shoemaker C, Newton JC and Xu DH. Detection and quantification of virulent Aeromonas hydrophila in channel catfish tissues following waterborne challenge. FEMS Microbiol Lett. 2016;363 9 doi:10.1093/femsle/fnw080.
34. Chen X, Zhong L, Bian C, Xu P, Qiu Y, You X, et al. High-quality genome assembly of channel catfish, Ictalurus punctatus. Gigascience. 2016;5 1:s13742-016-0142-5.
35. Zeng Q, Fu Q, Li Y, Waldbieser G, Bosworth B, Liu S, et al. Development of a 690 K SNP array in catfish and its application for genetic mapping and validation of the reference genome sequence. Sci Rep. 2017;7:40347. doi:10.1038/srep40347.
36. Liu S, Sun L, Li Y, Sun F, Jiang Y, Zhang Y, et al. Development of the catfish 250K SNP array for genome-wide association studies. BMC Res Notes. 2014;7:135. doi:10.1186/1756-0500-7-135.
37. Liu Z, Karsi A, Li P, Cao D and Dunham R. An AFLP-based genetic linkage map of channel catfish (Ictalurus punctatus) constructed by using an interspecific hybrid resource family. Genetics. 2003;165 2:687-94.
38. Schagdarsurengin U and Steger K. Epigenetics in male reproduction: effect of paternal diet on sperm quality and offspring health. Nature Reviews Urology. 2016;13 10:584-95.
39. Hu E, Yang H and Tiersch TR. High-throughput cryopreservation of spermatozoa of blue catfish (Ictalurus furcatus): establishment of an approach for commercial-scale processing. Cryobiology. 2011;62 1:74-82.
40. Bart AN and Dunham RA. Factors affecting survival of channel catfish after surgical removal of testes. The Progressive Fish-Culturist. 1990;52 4:241-6.
41. Abualreesh M, Myers JN, Gurbatow J, Johnson A, Xing D, Wang J, et al. Development of a spermatogonia cryopreservation protocol for blue catfish, Ictalurus furcatus. Cryobiology. 2020;97:46-52.
42. Butts I, Litvak M and Trippel E. Seasonal variations in seminal plasma and sperm characteristics of wild-caught and cultivated Atlantic cod, Gadus morhua. Theriogenology. 2010;73 7:873-85.
43. Butts I, Babiak I, Ciereszko A, Litvak M, Słowińska M, Soler C, et al. Semen characteristics and their ability to predict sperm cryopreservation potential of Atlantic cod, Gadus morhua L. Theriogenology. 2011;75 7:1290-300.
44. Butts IA, Alavi SMH, Mokdad A and Pitcher TE. Physiological functions of osmolality and calcium ions on the initiation of sperm motility and swimming performance in redside dace, Clinostomus elongatus. Comparative Biochemistry and Physiology Part A: Molecular & Integrative Physiology. 2013;166 1:147-57.
45. Butts IA, Prokopchuk G, Kašpar V, Cosson J and Pitcher TE. Ovarian fluid impacts flagellar beating and biomechanical metrics of sperm between alternative reproductive tactics. Journal of Experimental Biology. 2017;220 12:2210-7.
46. Butts IAE, Hilmarsdóttir GS, Zadmajid V, Gallego V, Støttrup JG, Jacobsen C, et al. Dietary amino acids impact sperm performance traits for a catadromous fish, Anguilla anguilla reared in captivity. Aquaculture. 2020;518:734602.
47. Woods III LC, Li Y, Ding Y, Liu J, Reading BJ, Fuller SA, et al. DNA methylation profiles correlated to striped bass sperm fertility. BMC genomics. 2018;19 1:244.
48. de Mello F, Garcia JS, Godoy LC, Depincé A, Labbé C and Streit Jr DP. The effect of cryoprotectant agents on DNA methylation patterns and progeny development in the spermatozoa of Colossoma macropomum. General and comparative endocrinology. 2017;245:94-101.
49. Cheng H, Concepcion GT, Feng X, Zhang H and Li H. Haplotype-resolved de novo assembly using phased assembly graphs with hifiasm. Nature Methods. 2021;18 2:170-5.
50. Weisenfeld NI, Kumar V, Shah P, Church DM and Jaffe DB. Direct determination of diploid genome sequences. Genome research. 2017;27 5:757-67.

51. Chakraborty M, Baldwin-Brown JG, Long AD and Emerson J. Contiguous and accurate de novo assembly of metazoan genomes with modest long read coverage. Nucleic acids research. 2016;44 19:e147-e.
52. Wang X, Kelkar YD, Xiong X, Martinson EO, Lynch J, Zhang C, et al. Genome report: Whole genome sequence and annotation of the parasitoid jewel wasp Nasonia giraulti laboratory strain RV2X [u]. G3: Genes, Genomes, Genetics. 2020;10 8:2565-72.
53. Brian B: BBMap: A Fast, Accurate, Splice-Aware Aligner. https://www.osti.gov/servlets/purl/1241166 (2014).
54. LeGrande WH, Dunham RA and Smitherman RO. Karyology of Three Species of Catfishes (Ictaluridae: Ictalurus) and Four Hybrid Combinations. Copeia. 1984;1984 4:873-8. doi:10.2307/1445331.
55. Liu S, Li Y, Qin Z, Geng X, Bao L, Kaltenboeck L, et al. High-density interspecific genetic linkage mapping provides insights into genomic incompatibility between channel catfish and blue catfish. Anim Genet. 2016;47 1:81-90. doi:10.1111/age.12372.
56. Liu Z, Karsi A and Dunham RA. Development of Polymorphic EST Markers Suitable for Genetic Linkage Mapping of Catfish. Mar Biotechnol (NY). 1999;1 5:437-0447. doi:10.1007/pl00011800.
57. Liu Z, Karsi A, Li P, Cao D and Dunham R. An AFLP-based genetic linkage map of channel catfish (Ictalurus punctatus) constructed by using an interspecific hybrid resource family. Genetics. 2003;165 2:687-94. doi:10.1093/genetics/165.2.687.
58. Ninwichian P, Peatman E, Liu H, Kucuktas H, Somridhivej B, Liu S, et al. Second-generation genetic linkage map of catfish and its integration with the BAC-based physical map. G3 (Bethesda). 2012;2 10:1233-41. doi:10.1534/g3.112.003962.
59. Waldbieser GC, Bosworth BG, Nonneman DJ and Wolters WR. A microsatellite-based genetic linkage map for channel catfish, Ictalurus punctatus. Genetics. 2001;158 2:727-34. doi:10.1093/genetics/158.2.727.
60. Karolchik D, Hinrichs AS, Furey TS, Roskin KM, Sugnet CW, Haussler D, et al. The UCSC Table Browser data retrieval tool. Nucleic Acids Res. 2004;32 Database issue:D493-6. doi:10.1093/nar/gkh103.
61. Walker BJ, Abeel T, Shea T, Priest M, Abouelliel A, Sakthikumar S, et al. Pilon: an integrated tool for comprehensive microbial variant detection and genome assembly improvement. PloS one. 2014;9 11:e112963.
62. Wang X, Xiong X, Cao W, Zhang C, Werren JH and Wang X. Genome assembly of the A-group Wolbachia in Nasonia oneida using linked-reads technology. Genome biology and evolution. 2019;11 10:3008-13.
63. Seppey M, Manni M and Zdobnov EM. BUSCO: assessing genome assembly and annotation completeness. Methods in molecular biology (Clifton, NJ). 2019;1962:227-45.
64. Bao L, Tian C, Liu S, Zhang Y, Elaswad A, Yuan Z, et al. The Y chromosome sequence of the channel catfish suggests novel sex determination mechanisms in teleost fish. BMC Biol. 2019;17 1:6. doi:10.1186/s12915-019-0627-7.
65. Gao Z, You X, Zhang X, Chen J, Xu T, Huang Y, et al. A chromosome-level genome assembly of the striped catfish (Pangasianodon hypophthalmus). Genomics. 2021;113 5:3349-56. doi:10.1016/j.ygeno.2021.07.026.
66. Kim OTP, Nguyen PT, Shoguchi E, Hisata K, Vo TTB, Inoue J, et al. A draft genome of the striped catfish, Pangasianodon hypophthalmus, for comparative analysis of genes relevant to development and a resource for aquaculture improvement. BMC Genomics. 2018;19 1:733. doi:10.1186/s12864-018-5079-x.
67. Wen M, Pan Q, Jouanno E, Montfort J, Zahm M, Cabau C, et al. An ancient truncated duplication of the anti-Mullerian hormone receptor type 2 gene is a potential conserved master sex

determinant in the Pangasiidae catfish family. Mol Ecol Resour. 2022; doi:10.1111/1755-0998.13620.
68. Zhou Y, Wang Y, Xiong X, Appel AG, Zhang C and Wang X. Profiles of telomeric repeats in Insecta reveal diverse forms of telomeric motifs in Hymenopterans. Life Sci Alliance. 2022;5 7 doi:10.26508/lsa.202101163.
69. Andrews S, Krueger F, Segonds-Pichon A, Biggins L, Krueger C and Wingett S. FastQC. 2010.
70. Bolger AM, Lohse M and Usadel B. Trimmomatic: a flexible trimmer for Illumina sequence data. Bioinformatics. 2014;30 15:2114-20.
71. Li H and Durbin R. Fast and accurate short read alignment with Burrows–Wheeler transform. bioinformatics. 2009;25 14:1754-60.
72. Li H. Minimap2: pairwise alignment for nucleotide sequences. Bioinformatics. 2018;34 18:3094-100. doi:10.1093/bioinformatics/bty191.
73. Li H. New strategies to improve minimap2 alignment accuracy. Bioinformatics. 2021; doi:10.1093/bioinformatics/btab705.
74. McKenna A, Hanna M, Banks E, Sivachenko A, Cibulskis K, Kernytsky A, et al. The Genome Analysis Toolkit: a MapReduce framework for analyzing next-generation DNA sequencing data. Genome research. 2010;20 9:1297-303.
75. Li H. A statistical framework for SNP calling, mutation discovery, association mapping and population genetical parameter estimation from sequencing data. Bioinformatics. 2011;27 21:2987-93.
76. Kim D, Pertea G, Trapnell C, Pimentel H, Kelley R and Salzberg SL. TopHat2: accurate alignment of transcriptomes in the presence of insertions, deletions and gene fusions. Genome biology. 2013;14 4:1-13.
77. Trapnell C, Roberts A, Goff L, Pertea G, Kim D, Kelley DR, et al. Differential gene and transcript expression analysis of RNA-seq experiments with TopHat and Cufflinks. Nature protocols. 2012;7 3:562-78.
78. Haas BJ, Papanicolaou A, Yassour M, Grabherr M, Blood PD, Bowden J, et al. De novo transcript sequence reconstruction from RNA-seq using the Trinity platform for reference generation and analysis. Nature protocols. 2013;8 8:1494-512.
79. Cantarel BL, Korf I, Robb SM, Parra G, Ross E, Moore B, et al. MAKER: an easy-to-use annotation pipeline designed for emerging model organism genomes. Genome research. 2008;18 1:188-96.
80. Zdobnov EM, Tegenfeldt F, Kuznetsov D, Waterhouse RM, Simao FA, Ioannidis P, et al. OrthoDB v9.1: cataloging evolutionary and functional annotations for animal, fungal, plant, archaeal, bacterial and viral orthologs. Nucleic Acids Res. 2017;45 D1:D744-D9. doi:10.1093/nar/gkw1119.
81. Korf I. Gene finding in novel genomes. BMC Bioinformatics. 2004;5:59. doi:10.1186/1471-2105-5-59.
82. Stanke M and Waack S. Gene prediction with a hidden Markov model and a new intron submodel. Bioinformatics. 2003;19 Suppl 2:ii215-25. doi:10.1093/bioinformatics/btg1080.
83. Stanke M, Schöffmann O, Morgenstern B and Waack S. Gene prediction in eukaryotes with a generalized hidden Markov model that uses hints from external sources. BMC Bioinformatics. 2006;7 1:62. doi:10.1186/1471-2105-7-62.
84. Howe KL, Achuthan P, Allen J, Allen J, Alvarez-Jarreta J, Amode MR, et al. Ensembl 2021. Nucleic Acids Res. 2021;49 D1:D884-D91. doi:10.1093/nar/gkaa942.
85. Keilwagen J, Hartung F and Grau J. GeMoMa: Homology-Based Gene Prediction Utilizing Intron Position Conservation and RNA-seq Data. Methods Mol Biol. 2019;1962:161-77. doi:10.1007/978-1-4939-9173-0_9.
86. Kent WJ. BLAT--the BLAST-like alignment tool. Genome Res. 2002;12 4:656-64. doi:10.1101/gr.229202.

87. Kalvari I, Nawrocki EP, Ontiveros-Palacios N, Argasinska J, Lamkiewicz K, Marz M, et al. Rfam 14: expanded coverage of metagenomic, viral and microRNA families. Nucleic Acids Res. 2021;49 D1:D192-D200. doi:10.1093/nar/gkaa1047.
88. Chan PP, Lin BY, Mak AJ and Lowe TM. tRNAscan-SE 2.0: improved detection and functional classification of transfer RNA genes. Nucleic Acids Res. 2021;49 16:9077-96. doi:10.1093/nar/gkab688.
89. Yin L, Zhang H, Tang Z, Xu J, Yin D, Zhang Z, et al. rMVP: A Memory-efficient, Visualization-enhanced, and Parallel-accelerated tool for Genome-wide Association Study. Genomics Proteomics Bioinformatics. 2021; doi:10.1016/j.gpb.2020.10.007.
90. Zhang Q. Cytogenetic and molecular analysis of the channel catfish (Ictalurus punctatus) genome. Louisiana State University and Agricultural & Mechanical College; 1996.
91. Darling AC, Mau B, Blattner FR and Perna NT. Mauve: multiple alignment of conserved genomic sequence with rearrangements. Genome research. 2004;14 7:1394-403.
92. Wang Y, Tang H, DeBarry JD, Tan X, Li J, Wang X, et al. MCScanX: a toolkit for detection and evolutionary analysis of gene synteny and collinearity. Nucleic acids research. 2012;40 7:e49-e.
93. Krzywinski M, Schein J, Birol I, Connors J, Gascoyne R, Horsman D, et al. Circos: an information aesthetic for comparative genomics. Genome research. 2009;19 9:1639-45.
94. Flynn JM, Hubley R, Goubert C, Rosen J, Clark AG, Feschotte C, et al. RepeatModeler2 for automated genomic discovery of transposable element families. Proceedings of the National Academy of Sciences. 2020;117 17:9451-7.
95. Tarailo-Graovac M and Chen N. Using RepeatMasker to identify repetitive elements in genomic sequences Curr Protoc Bioinformatics. 2009. Chapter.
96. Kriventseva EV, Kuznetsov D, Tegenfeldt F, Manni M, Dias R, Simão FA, et al. OrthoDB v10: sampling the diversity of animal, plant, fungal, protist, bacterial and viral genomes for evolutionary and functional annotations of orthologs. Nucleic Acids Research. 2018;47 D1:D807-D11. doi:10.1093/nar/gky1053.
97. Katoh K and Standley DM. MAFFT: iterative refinement and additional methods. Multiple sequence alignment methods. Springer; 2014. p. 131-46.
98. Nguyen L-T, Schmidt HA, Von Haeseler A and Minh BQ. IQ-TREE: a fast and effective stochastic algorithm for estimating maximum-likelihood phylogenies. Molecular biology and evolution. 2015;32 1:268-74.
99. Rambaut A. FigTree v1. 3.1. http://tree bio ed ac uk/software/figtree/. 2009.
100. Buchfink B, Xie C and Huson DH. Fast and sensitive protein alignment using DIAMOND. Nature methods. 2015;12 1:59-60.
101. Li L, Stoeckert CJ and Roos DS. OrthoMCL: identification of ortholog groups for eukaryotic genomes. Genome research. 2003;13 9:2178-89.
102. Sanderson MJ. r8s: inferring absolute rates of molecular evolution and divergence times in the absence of a molecular clock. Bioinformatics. 2003;19 2:301-2.
103. Hedges SB, Marin J, Suleski M, Paymer M and Kumar S. Tree of life reveals clock-like speciation and diversification. Molecular biology and evolution. 2015;32 4:835-45.
104. De Bie T, Cristianini N, Demuth JP and Hahn MW. CAFE: a computational tool for the study of gene family evolution. Bioinformatics. 2006;22 10:1269-71.
105. Huerta-Cepas J, Forslund K, Coelho LP, Szklarczyk D, Jensen LJ, Von Mering C, et al. Fast genome-wide functional annotation through orthology assignment by eggNOG-mapper. Molecular biology and evolution. 2017;34 8:2115-22.
106. Meyer A and Van de Peer Y. From 2R to 3R: evidence for a fish-specific genome duplication (FSGD). Bioessays. 2005;27 9:937-45. doi:10.1002/bies.20293.

107. Wang S, Peatman E, Abernathy J, Waldbieser G, Lindquist E, Richardson P, et al. Assembly of 500,000 inter-specific catfish expressed sequence tags and large scale gene-associated marker development for whole genome association studies. Genome biology. 2010;11 1:1-14.
108. Yuan Z, Zhou T, Bao L, Liu S, Shi H, Yang Y, et al. The annotation of repetitive elements in the genome of channel catfish (Ictalurus punctatus). PLoS One. 2018;13 5:e0197371. doi:10.1371/journal.pone.0197371.
109. Gao B, Wang Y, Diaby M, Zong W, Shen D, Wang S, et al. Evolution of pogo, a separate superfamily of IS630-Tc1-mariner transposons, revealing recurrent domestication events in vertebrates. Mob DNA. 2020;11:25. doi:10.1186/s13100-020-00220-0.
110. Zhou T, Li N, Jin Y, Zeng Q, Prabowo W, Liu Y, et al. Chemokine C-C motif ligand 33 is a key regulator of teleost fish barbel development. Proc Natl Acad Sci U S A. 2018;115 22:E5018-E27. doi:10.1073/pnas.1718603115.
111. Khalil K, Elayat M, Khalifa E, Daghash S, Elaswad A, Miller M, et al. Generation of Myostatin Gene-Edited Channel Catfish (Ictalurus punctatus) via Zygote Injection of CRISPR/Cas9 System. Sci Rep. 2017;7 1:7301. doi:10.1038/s41598-017-07223-7.
112. Simora RMC, Xing, Bangs MR, Wang W, Ma X, Su B, et al. CRISPR/Cas9-mediated knock-in of alligator cathelicidin gene in a non-coding region of channel catfish genome. Sci Rep. 2020;10 1:22271. doi:10.1038/s41598-020-79409-5.
113. Elaswad A, Khalil K, Ye Z, Liu Z, Liu S, Peatman E, et al. Effects of CRISPR/Cas9 dosage on TICAM1 and RBL gene mutation rate, embryonic development, hatchability and fry survival in channel catfish. Sci Rep. 2018;8 1:16499. doi:10.1038/s41598-018-34738-4.
114. Darwin C. The Works of Charles Darwin, Volume 25: The Effects of Cross and Self Fertilization in the Vegetable Kingdom. NYU Press; 2010.
115. Wang X and Clark AG. Using next-generation RNA sequencing to identify imprinted genes. Heredity (Edinb). 2014;113 2:156-66. doi:10.1038/hdy.2014.18.
116. Hughes LC, Ortí G, Huang Y, Sun Y, Baldwin CC, Thompson AW, et al. Comprehensive phylogeny of ray-finned fishes (Actinopterygii) based on transcriptomic and genomic data. Proceedings of the National Academy of Sciences. 2018;115 24:6249-54.
117. Jiang Y, Gao X, Liu S, Zhang Y, Liu H, Sun F, et al. Whole genome comparative analysis of channel catfish (Ictalurus punctatus) with four model fish species. BMC genomics. 2013;14 1:1-11.
118. Shao F, Pan H, Li P, Ni L, Xu Y and Peng Z. Chromosome-Level Genome Assembly of the Asian Red-Tail Catfish (Hemibagrus wyckioides). Front Genet. 2021;12:747684. doi:10.3389/fgene.2021.747684.
119. Gong G, Dan C, Xiao S, Guo W, Huang P, Xiong Y, et al. Chromosomal-level assembly of yellow catfish genome using third-generation DNA sequencing and Hi-C analysis. GigaScience. 2018;7 11:giy120.
120. Jiang W, Lv Y, Cheng L, Yang K, Bian C, Wang X, et al. Whole-Genome Sequencing of the Giant Devil Catfish, Bagarius yarrelli. Genome Biol Evol. 2019;11 8:2071-7. doi:10.1093/gbe/evz143.
121. Duong TY, Tan MH, Lee YP, Croft L and Austin CM. Dataset for genome sequencing and de novo assembly of the Vietnamese bighead catfish (Clarias macrocephalus Gunther, 1864). Data Brief. 2020;31:105861. doi:10.1016/j.dib.2020.105861.
122. Kushwaha B, Pandey M, Das P, Joshi CG, Nagpure NS, Kumar R, et al. The genome of walking catfish Clarias magur (Hamilton, 1822) unveils the genetic basis that may have facilitated the development of environmental and terrestrial adaptation systems in air-breathing catfishes. DNA Res. 2021;28 1 doi:10.1093/dnares/dsaa031.
123. Li N, Bao L, Zhou T, Yuan Z, Liu S, Dunham R, et al. Genome sequence of walking catfish (Clarias batrachus) provides insights into terrestrial adaptation. BMC Genomics. 2018;19 1:952. doi:10.1186/s12864-018-5355-9.

124. Hufton AL, Groth D, Vingron M, Lehrach H, Poustka AJ and Panopoulou G. Early vertebrate whole genome duplications were predated by a period of intense genome rearrangement. Genome Research. 2008;18 10:1582-91.

125. Dunham RA and Smitherman RO. Ancestry and breeding of catfish in the United States. Ancestry and breeding of catfish in the United States. 1984; 273.

126. Bosworth B, Quiniou S and Chatakondi N. Effects of Season, Strain, and Body Weight on Testes Development and Quality in Three Strains of Blue Catfish, Ictalurus furcatus. Journal of the World Aquaculture Society. 2018;49 1:175-82. doi:https://doi.org/10.1111/jwas.12419.

127. Dunham RA, Ramboux AC and Perera DA. Effect of strain on tolerance of low dissolved oxygen of channel X blue catfish hybrids. Aquaculture. 2014;420:S25-S8.

128. Ramboux ACR. Evaluation of four genetic groups of channel-blue catfish hybrids grown in earthen ponds. 1992.

129. Chatakondi NG, Yant RD and Dunham RA. Effect of Paternal Blue Catfish Strain Effects on Hatchery Fry Production and Performance of Channel Catfish × Blue Catfish F1 Hybrid Fry Production and Fingerling Performance Under Commercial Conditions. North American Journal of Aquaculture. 2016;78 4:301-6. doi:https://doi.org/10.1080/15222055.2016.1185065.

130. Dunham RA, Hyde C, Masser M, Plumb JA, Smitherman RO, Perez R, et al. Comparison of culture traits of channel catfish, Ictalurus punctatus, and blue catfish I. furcatus. Journal of Applied Aquaculture. 1994;3 3-4:257-68.

131. Xu DH, Klesius P, Bosworth BG and Chatakondi N. Susceptibility of three strains of blue catfish, Ictalurus furcatus (Valenciennes), to I chthyophthirius multifiliis. Journal of fish diseases. 2012;35 12:887-95.

Table 1. Summary statistics of the blue catfish (*Ictalurus furcatus*) genome assemblies.

**Genome assembly**	**Blue catfish** (this assembly)	**Channel catfish** (ASM400665v3)	**Tra catfish** (GCA_016801045.1)
**Sequencing data and coverage**			
PacBio sequencing data	21.34 Gb PacBio Sequel II CCS HiFi reads	57.69 Gb PacBio Sequel CLS reads	63.07 Gb Nanopore
Illumina sequencing data	49.41Gb NovaSeq reads	-	44.23 Gb HiSeq reads
Genome coverage	CCS: 24×; Illumina: 59×	CLS: 58×	Nanopore: 130×; Illumina: 59×
**Assembly statistics**			
Total scaffold length	841,864,377 bp	1,036,985,268 bp	742,562,378 bp
Total contig length	838,964,151 bp	1,018,280,134 bp	771,909,303 bp
Number of scaffolds	271	3,164	402
Scaffold N50	28.24 Mbp	26.68 Mbp	29.53 Mbp
Maximum scaffold length	38.50 Mbp	39.13 Mbp	45.06 Mbp
Number of contigs	563	6,999	808
Contig N50	8.59 Mbp	1.70 Mbp	3.48 Mbp
Maximum contig length	24.51 Mbp	24.09 Mbp	16.11 Mbp
**Completeness**			
BUSCO completeness	98.6%	97.1%	93.3%
single-copy BUSCO	98.6%	95.8%	89.6%
duplicated BUSCO	0%	1.3%	3.7%
fragmented BUSCO	0%	1.0%	2.5%
missing BUSCO	1.4%	1.9%	4.2%

Table 2. Summary of predicted non-coding RNA genes annotated in blue catfish, *Ictalurus furcatus*, genome.

Non-coding RNAs	Counts (full copies)	Average unit length	Total length	Percent of genome
**tRNA genes**				
tRNAs decoding standard 20 amino acids	1,712	75 bp	128,864 bp	0.015%
Selenocysteine tRNAs	8	75 bp	597 bp	<0.001%
tRNAs with undetermined isotypes	13	73 bp	953 bp	<0.001%
suppressor tRNAs	10	81 bp	813 bp	<0.001%
predicted pseudogenes	4,359	75 bp	338,738 bp	0.040%
**tRNA total**	6,102	-	469,965 bp	0.056%
**rRNA genes**				
28S rRNA	55	4,180 bp	229,935 bp	0.027%
18S rRNA	55	1,872 bp	102,983 bp	0.012%
5.8S rRNA	57	154 bp	8,782 bp	0.001%
5S rRNA	12,448	117 bp	1,451,889 bp	0.173%
**rRNA total**	12,615	-	1,793,589 bp	0.214%
**snRNA genes**				
U1	74	163 bp	12,047 bp	0.001%
U2	215	215 bp	39,363 bp	0.005%
U4	42	141 bp	5,903 bp	0.001%
U5	207	115 bp	23,753 bp	0.003%
U6	51	106 bp	5,390 bp	0.001%
Other	12	105 bp	1,258 bp	<0.001%
**snRNA total**	601	-	87,714 bp	0.010%
**snoRNA genes**				
C/D-box snoRNA	135	123 bp	17,611 bp	0.002%
H/ACA-box snoRNA	72	150 bp	9,572 bp	0.001%
**snoRNA total**	207	-	27,183 bp	0.003%
**miRNA genes**	2079	81 bp	167,979 bp	0.020%

Table 3. Evaluation of 690K catfish SNP array probes for the channel catfish, *Ictalurus punctatus*, genome, blue catfish, *I. furcatus*, genome, and channel-blue informative SNPs.

**Catfish 690K SNP array statistics**	**Channel catfish** (IpCoco v1.2)	**Blue catfish** (this assembly)
Total number of probes	693,567	693,567
Number (%) of probes mapped to genome	686,675 (99.0%)	203,559 (29.3%)
Number (%) of probes mapped to genome without indels	685,939 (98.9%)	201,580 (29.1%)
Number (%) of probes mapped to multiple regions	4,560 (0.7%)	1,921 (0.9%)
Number (%) of probes with mismatches in SNP flanking sequence	677 (0.1%)	33,731 (16.8%)
Number (%) of probes with unique/perfect hit (**valid set**)	680,856 (98.2%)	165,928 (23.9%)
Number (%) of probes for channel-blue SNPs (**informative set**)	253,292 (36.5%)	76,399 (11.0%)
Number (%) of probes for blue catfish within-species SNPs	-	2,978 (1.80%)

Table 4. Summary repeat element classes in blue catfish (*Ictalurus furcatus*), channel catfish (*Ictalurus punctatus*), and tra catfish (*Pangasianodon hypophthalmus*) genomes.

	Ictalurus furcatus (this assembly)		***Ictalurus punctatus*** (ASM400665v3)		***Pangasianodon hypophthalmus*** (GCA_016801045.1)	
	# of elements	Length (%)	# of elements	Length (%)	# of elements	Length (%)
**Retroelements**						
Penelope	1,717	838,078 (0.1%)	1,214	922,008 (0.09%)	1,988	488,401 (0.07%)
LINEs	58,734	24,968,273 (2.96%)	89,843	35,225,182 (3.53%)	47,975	18,520,943 (2.49%)
L2/CR1/Rex	43,100	16,253,105 (1.93%)	57,766	20,111,601 (2.01%)	37,713	14,893,881 (2.01%)
R1/LOA/Jockey	763	350,294 (0.04%)	2,542	1,043,469 (0.1%)	537	175,472 (0.02%)
R2/R4/NeSL	592	266,497 (0.03%)	691	234,736 (0.02%)	576	228,226 (0.03%)
RTE/Bov-B	3,485	1,381,797 (0.16%)	4,718	1,666,345 (0.17%)	4,156	1,491,021 (0.02%)
L1/CIN4	2,354	2,261,930 (0.27%)	10,403	4,543,711 (0.46%)	799	348,555 (0.05%)
**LTR elements**						
BEL/Pao	380	241,716 (0.03%)	207	336,942 (0.03)	193	177,582 (0.02%)
Retroviral	4,006	3,275,694 (0.39%)	3,719	4,848,411 (0.49%)	497	398,835 (0.05%)
Gypsy/DIRS1	32,774	19,609,302 (2.33%)	39,817	22,167,664 (2.22%)	21,919	9,258,407 (1.25%)
**DNA transposons**						
hobo-Activator	21,792	5,726,207 (0.68%)	18,044	5,537,205 (0.55%)	10,850	2,191,374 (0.3%)
Tc1-IS630-Pogo	189,344	68,689,788 (8.16%)	213,390	78,079,336 (7.82%)	11,2905	34,735,533 (4.68%)
PiggyBac	906	347,427 (0.04%)	552	215,961 (0.02%)	323	138,058 (0.02%)
Tourist/Harbinger	2,084	586,919 (0.07%)	3,613	589,965 (0.06%)	2,226	637,457 (0.09%)
**Unclassified**	958,235	219,639,183 (26.08%)	1,197,919	263,899,746 (26.43%)	932,206	186,441,198 (25.11%)
**Simple repeats**	575,417	27,950,071 (3.32%)	706,627	34,869,604 (3.49%)	544,794	28,043,320 (3.78%)
**Low complexity**	43,673	3,139,550 (0.37%)	52,048	3,441,656 (0.34%)	42,087	2,746,467 (0.37%)
**Total**	1,939,356	395,525,831 (46.96%)	2,403,113	477,733,542 (50.8%)	1,761,744	300,914,730 (40.36%)

Figure legends

Figure 1. Markers in the genetic linkage map of channel catfish, *Ictalurus punctatus*, and blue catfish, *I. furcatus*.
(**A**) The linkage map position (*y*-axis) and physical location (*x*-axis) of 84 channel catfish genetic makers and 55 blue catfish genetic markers on chromosome 1.
(**B**) The linkage map position (*y*-axis) and physical location (*x*-axis) of 76 channel catfish genetic makers and 43 blue catfish genetic markers on chromosome 2.
(**C**) A total of 1,739 and 1,009 molecular markers were anchored onto 29 chromosomes in channel catfish (orange bar) and blue catfish (blue bar), respectively. The brown bars represent the shared makers in both channel catfish and blue catfish.

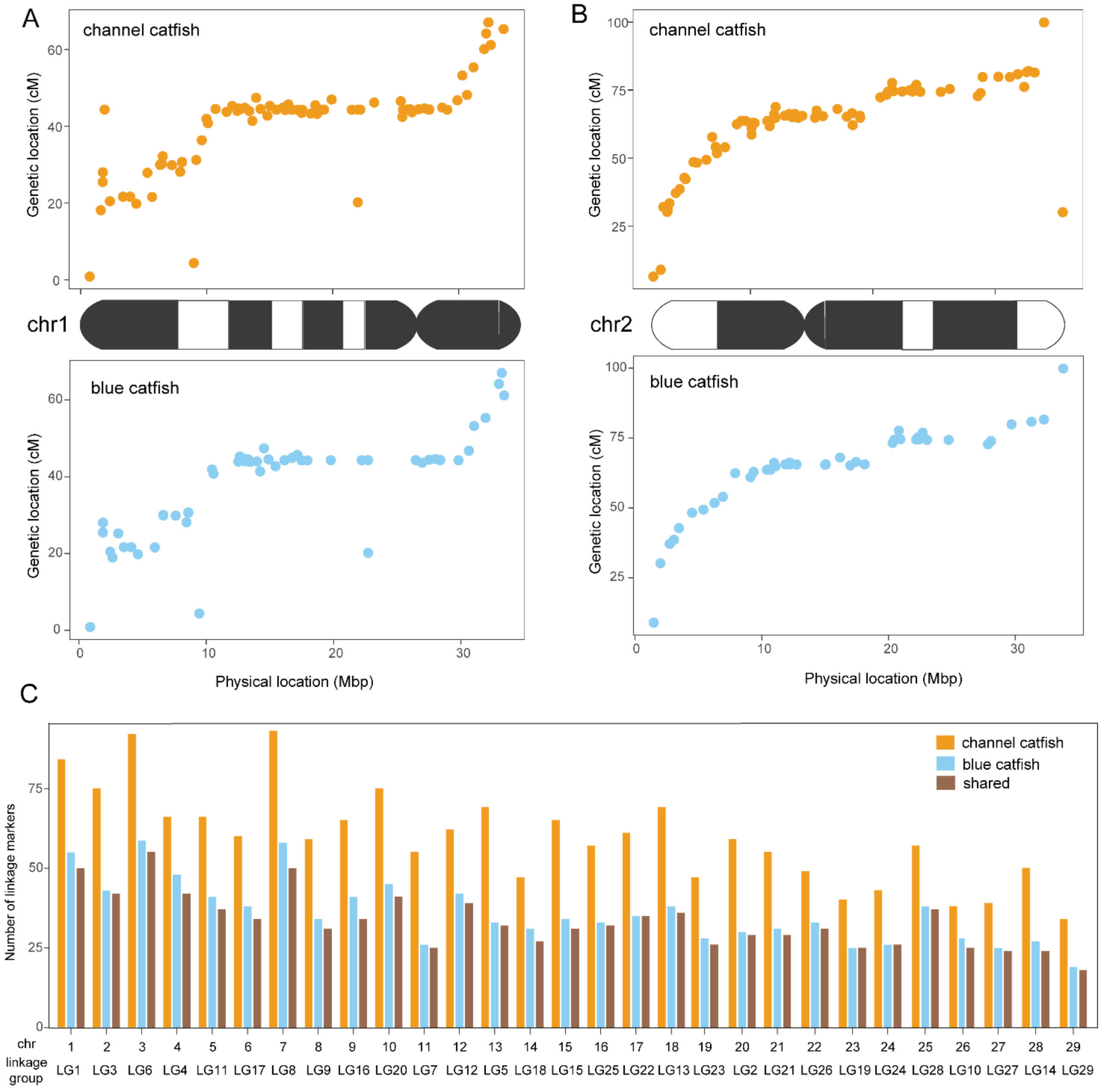

Figure 2. Synteny alignments of blue catfish and channel catfish chromosomes based on DNA sequence similarity.

(**A**) Presence and absence of telomeric repeat motif (TRM) at termini of assembled chromosomes shown in karyogram. The orange boxes represent channel catfish telomere, and blue boxes represent blue catfish telomeric assembly. (B-C) Synteny analysis of chromosome 1 and 2 between channel catfish and blue catfish (synteny analyses for remaining chromsomes were showed in Figure S2).

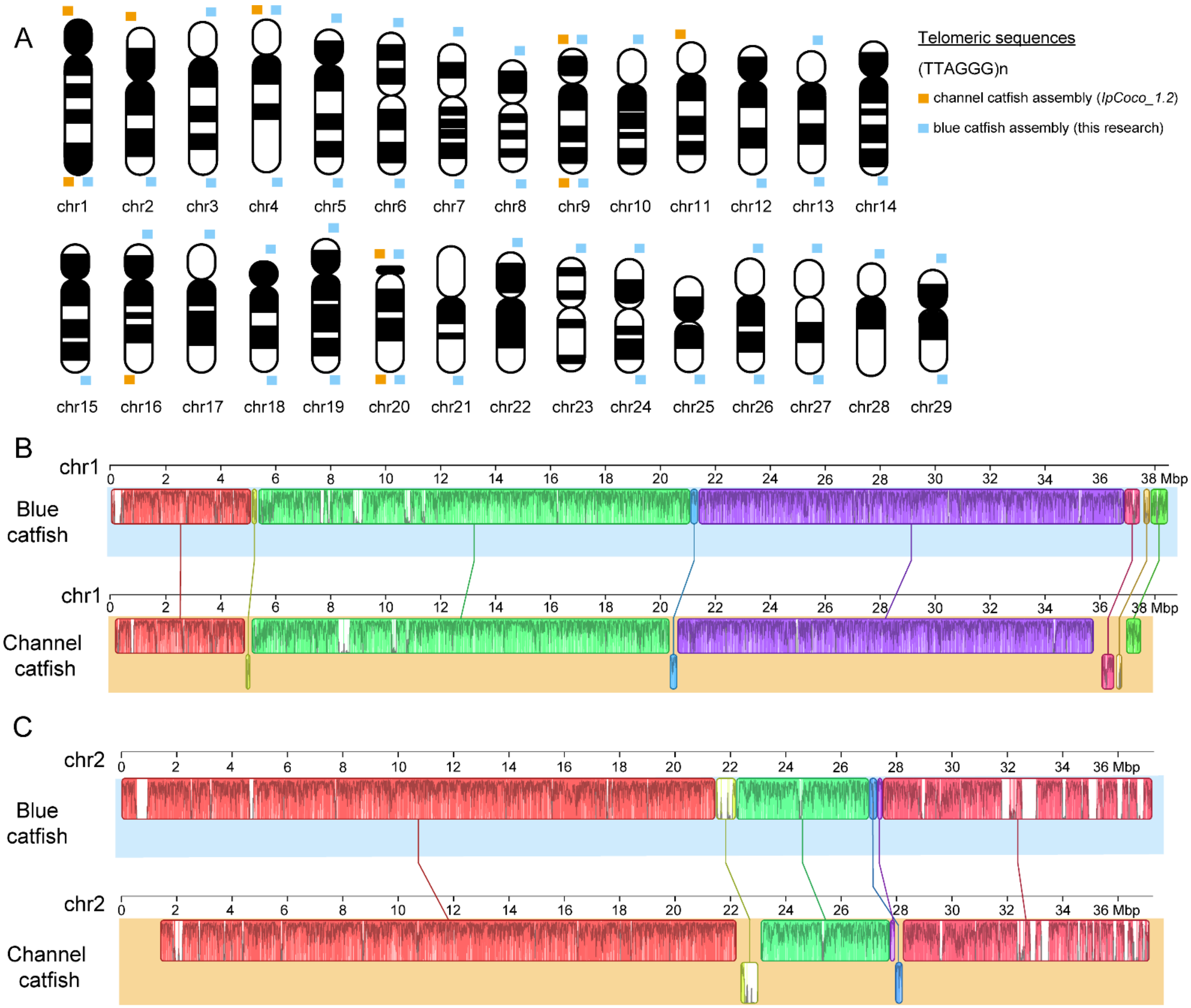

Figure 3. Genome comparisons between channel catfish, *Ictalurus punctatus,* and blue catfish, *I. furcatus,* based on orthologous genes.

A total of 29 chromosomes in the blue catfish genome assembly (on the left in the Circos plot) showed a one-to-one homologous relationship with 29 chromosomes in the channel catfish genome (on the right in the Circos plot). The outside ring represents chromosomes.

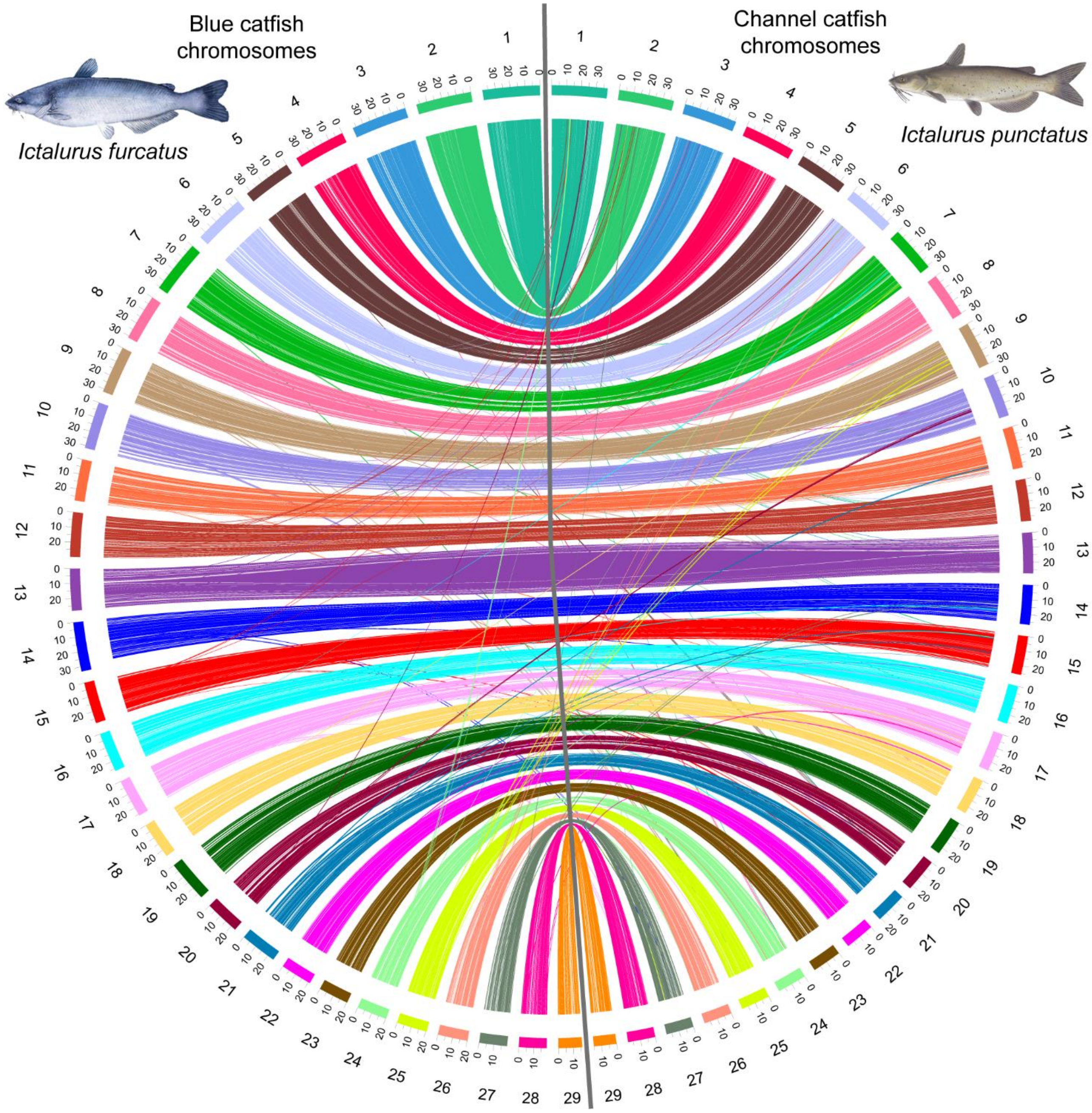

Figure 4. Density plots of channel-blue catfish (*Ictalurus*) genome-wide SNPs and informative SNPs covered by catfish 690K array.

(**A**) SNP density plot showing the distribution of polymorphisms per kilobase pairs across 29 chromosomes. The scale for the number of SNPs is shown on the right. (**B**) Density plot showing the distribution of informative, perfect-match SNP probes per 100 kilobase pairs across 29 chromosomes. The scale for the number of SNPs is shown on the right.

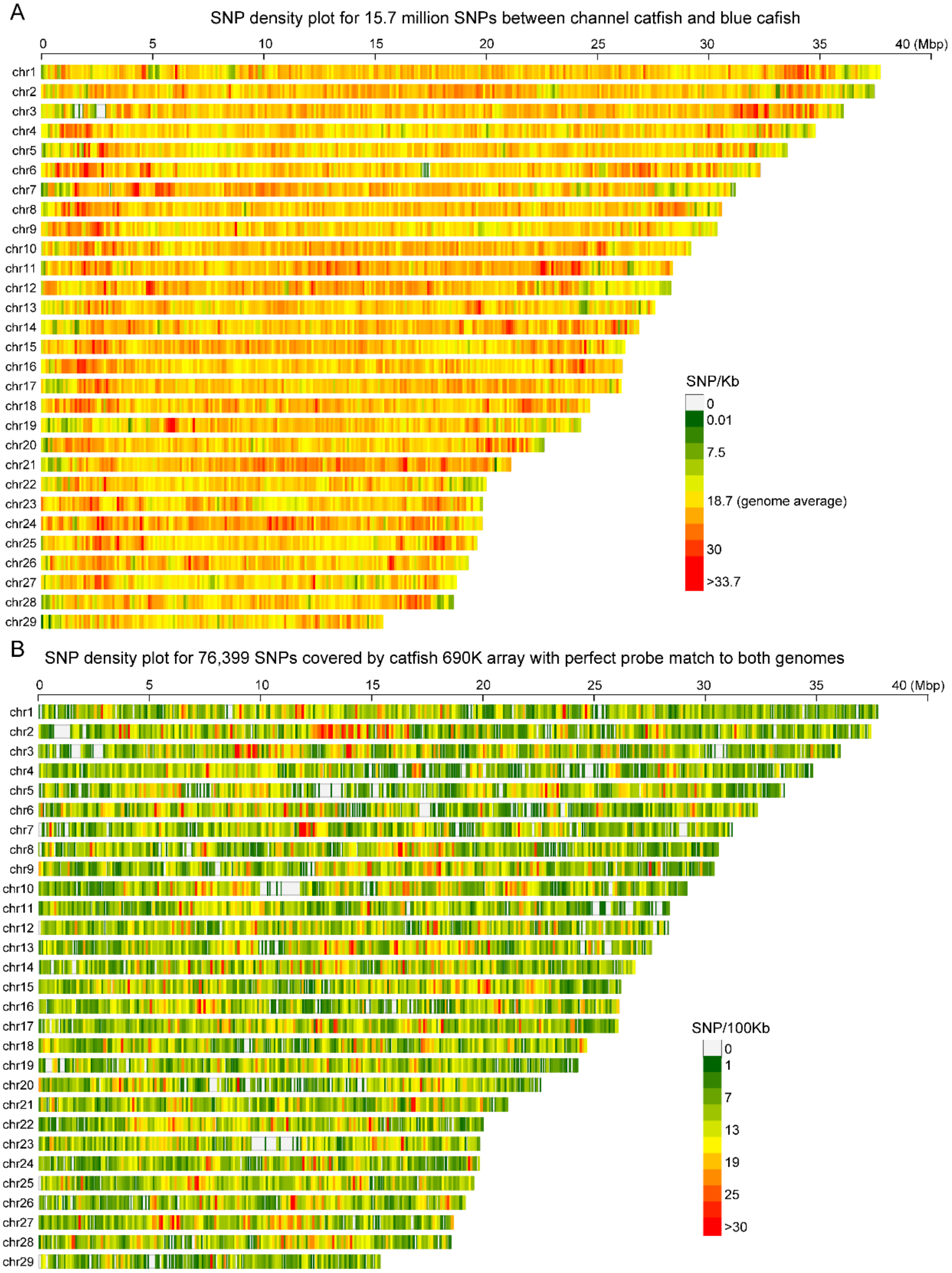

Figure 5. Phylogenetic relationship between *Ictalurus furcatus* and ten representative Actinopterygii species.

A maximum-likelihood phylogenetic tree of *Ictalurus furcatus* with ten other Actinopterygii species was constructed based on 4,698 shared 1:1 single-copy proteins using IQ-TREE. The bootstrap values are supported at 100/100. The length of each branch is shown on the branches. The spotted gar was used as the outgroup.

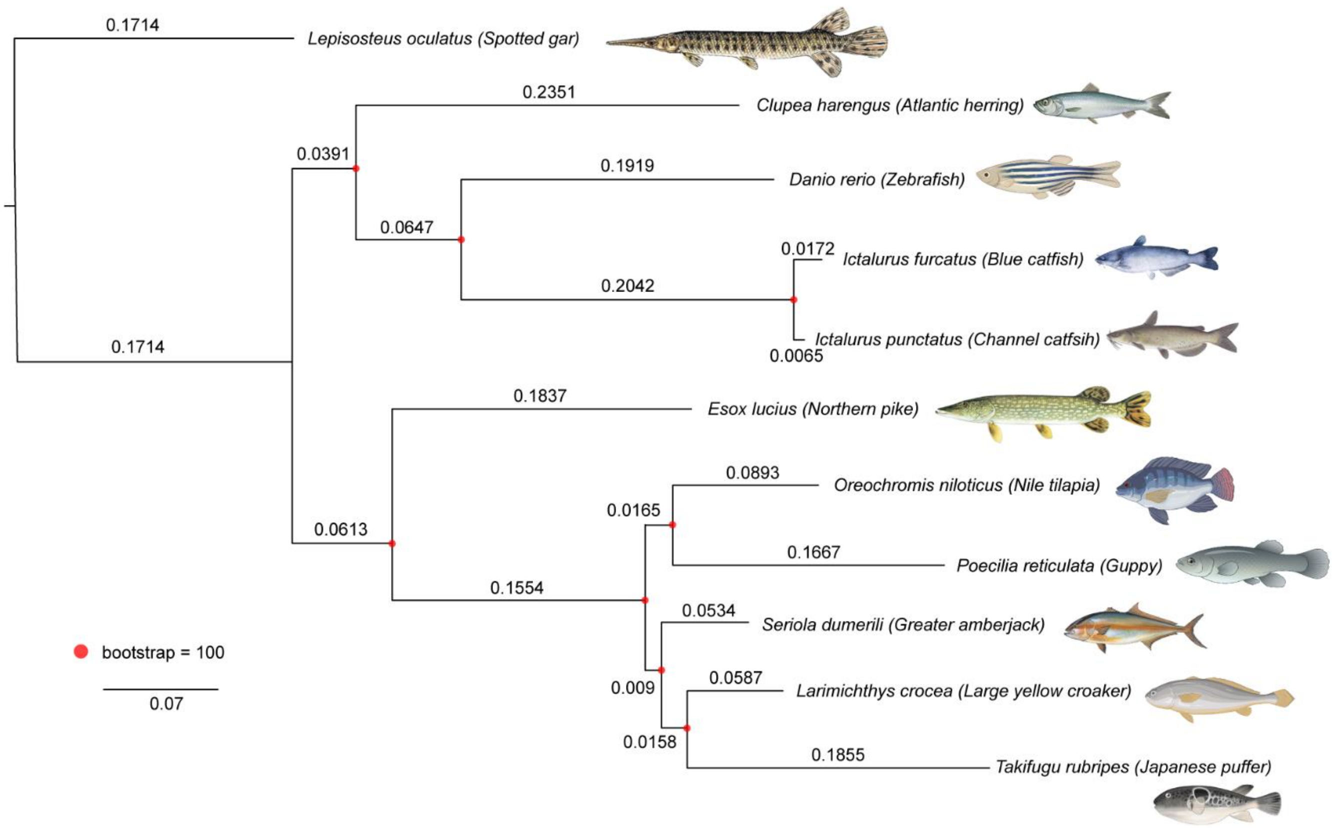

Figure 6. Gene family expansion and contraction analysis of blue catfish *Ictalurus furcatus*.

Gene family evolution was analyzed using CAFÉ. The number of gene family expansions and contractions are shown in bubble plot for each species. The phylogenetic tree of the blue catfish with other 10 Actinopterygii species was constructed using 4,698 single-copy orthologous genes. The species divergence time was estimated and labeled at each branch site (millions of years ago). The calibration nodes for divergence time are labeled in red from fossil evidence.

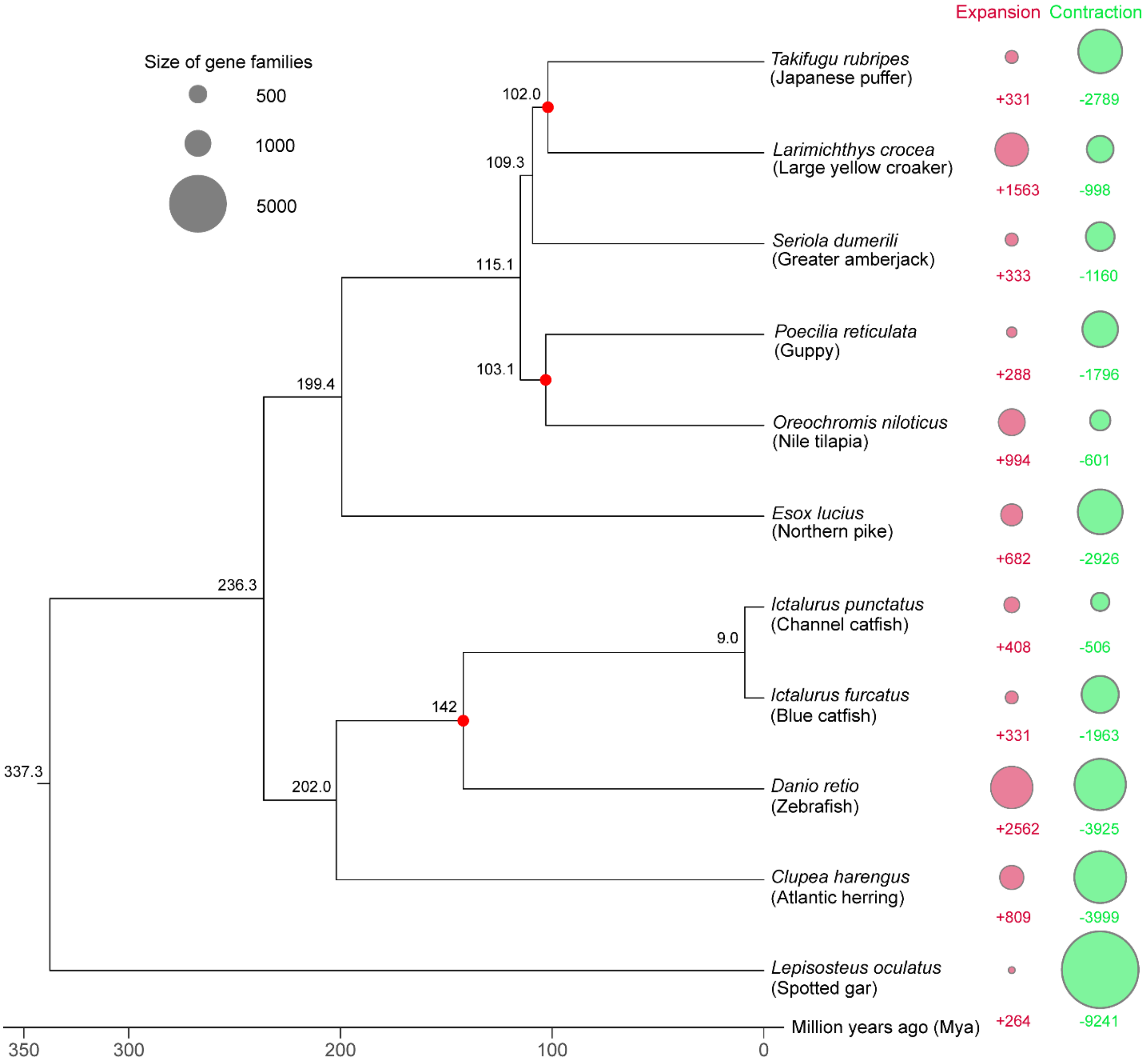

List of Supplemental Materials

Figure S1. Circos plot showing paralogous gene pairs in the blue catfish genome.

Figure S2. Synteny alignments of blue catfish and channel catfish chromosomes based on DNA sequence similarity.

Table S1. Summary of PacBio and Illumina (10× Genomics) sequencing data generated for blue catfish genome assembly.

Table S2. Chromosomal locations of telomeric regions in the blue catfish genome.

Table S3. Chromosomal locations of telomeric regions in the channel catfish genome.

Table S4. RNA sequencing data yield, quality control, and alignment statistics to channel catfish and blue catfish genomes.

Table S5. Summary nucleotide substitutions in the mitochondrial genome between blue catfish D&B and Rio Grande strains.

Table S6. Summary nucleotide substitutions in the mitochondrial genome between blue catfish and channel catfish.

Table S7. Summary of gene family expansion and contraction results.

Table S8. List of gene families underwent rapid expansion in blue catfish.

Data S1. Locations of 1800 linkage markers of *Ictalurus punctatus* × *Ictalurus furcatus* crosses in the channel catfish and blue catfish genomes.

Data S2. Annotation of tRNA genes in blue catfish genome.

Data S3. Annotation of rRNA gene clusters and 5S rRNA genes in blue catfish genome.

Data S4. Annotation of snRNA genes in blue catfish genome.

Data S5. Annotation of snoRNA genes in blue catfish genome.

Data S6. Annotation of miRNA genes in blue catfish genome.

Data S7. List of genes of significant expansion gene family in blue catfish and their annotation from orthoDB and eggNOG-mapper.

Click here to access/download
Supplementary Material
Supplemental_materials.pdf
