# Supplementary material for: Chromosome-level assembly and annotation of the blue catfish Ictalurus furcatus, an aquaculture species for hybrid catfish reproduction, epigenetics, and heterosis studies
Source: Gigascience. 2022 Jul 9;11:giac070. doi: 10.1093/gigascience/giac070 (PMC9270728; doi:10.1093/gigascience/giac070)
Supplement: giac070_Supplemental_Files [file giac070_supplemental_files.zip › Supplemental_materials.pdf]

Figure S1. Circos plot showing paralogous gene pairs in the blue catfish genome.

Colored lines join the gene pairs resulting from whole genome duplication. Chr = chromosome.

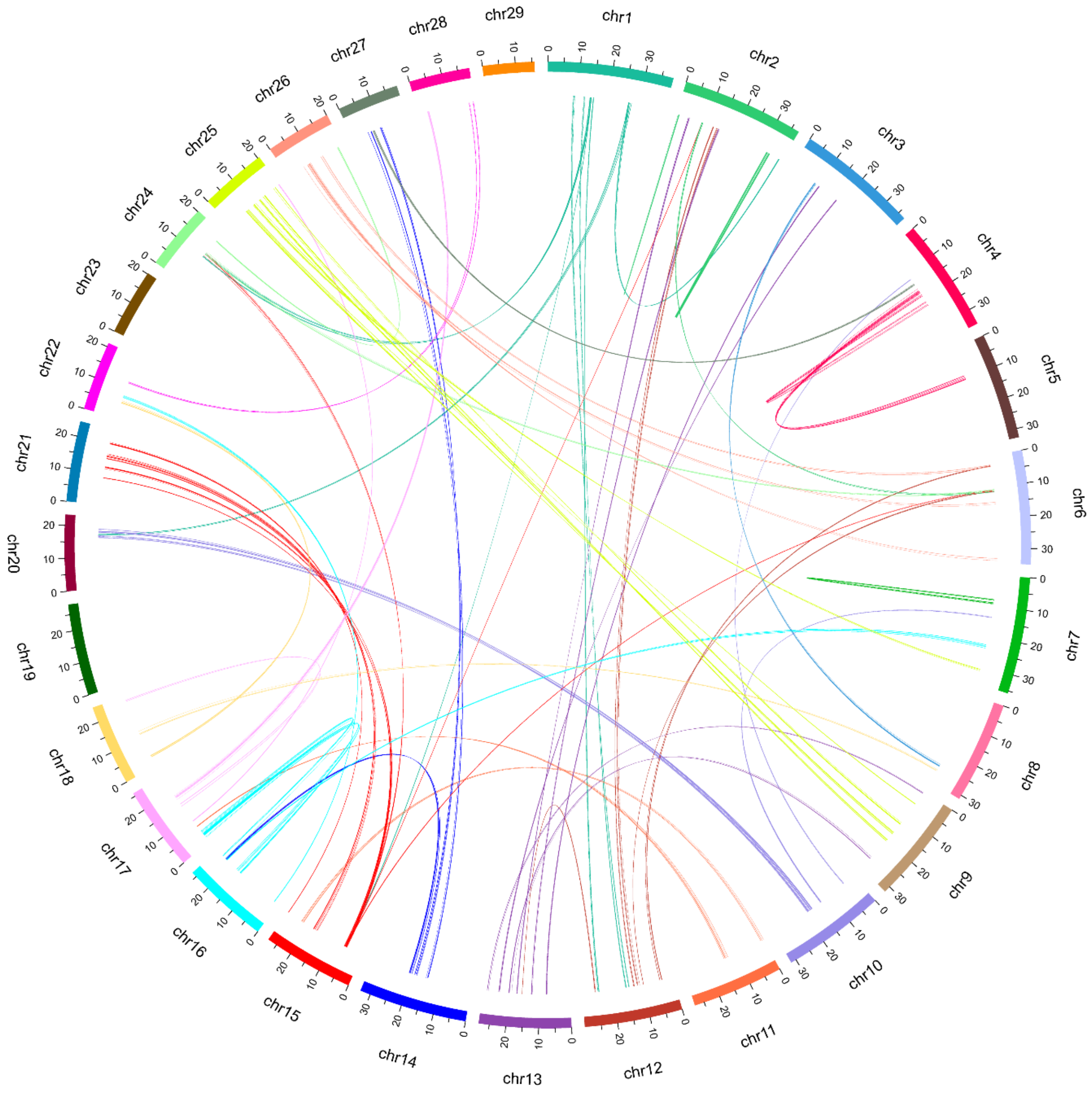

Figure S2. Synteny alignments of blue catfish and channel catfish chromosomes based on DNA sequence similarity.

(A). Synteny analysis of chromosome 3~8 between channel catfish and blue catfish (unit: Mbp).

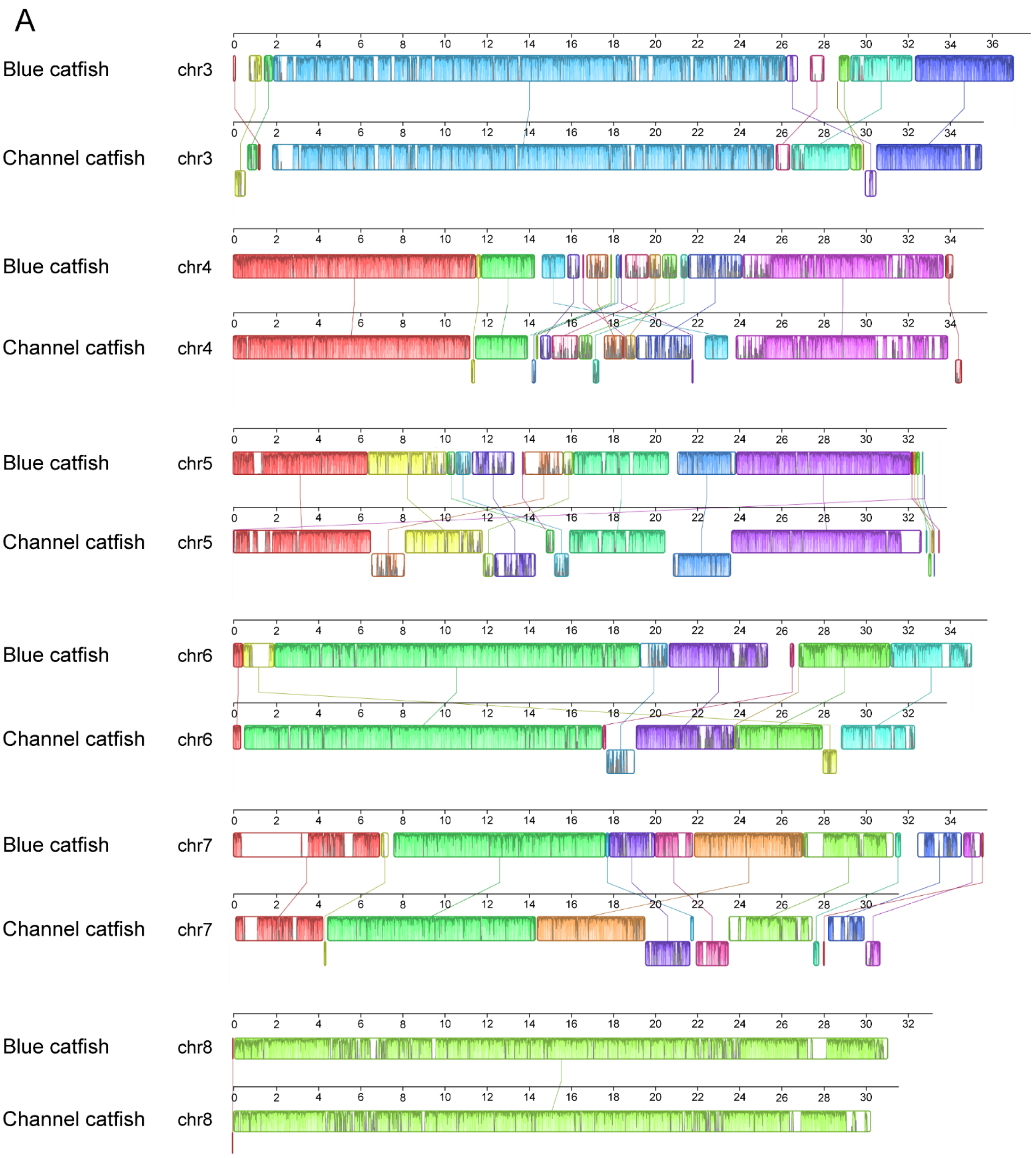

Figure S2 (continued). Synteny alignments of blue catfish and channel catfish chromosomes based on DNA sequence similarity.

(B). Synteny analysis of chromosome 9~15 between channel catfish and blue catfish (unit: Mbp).

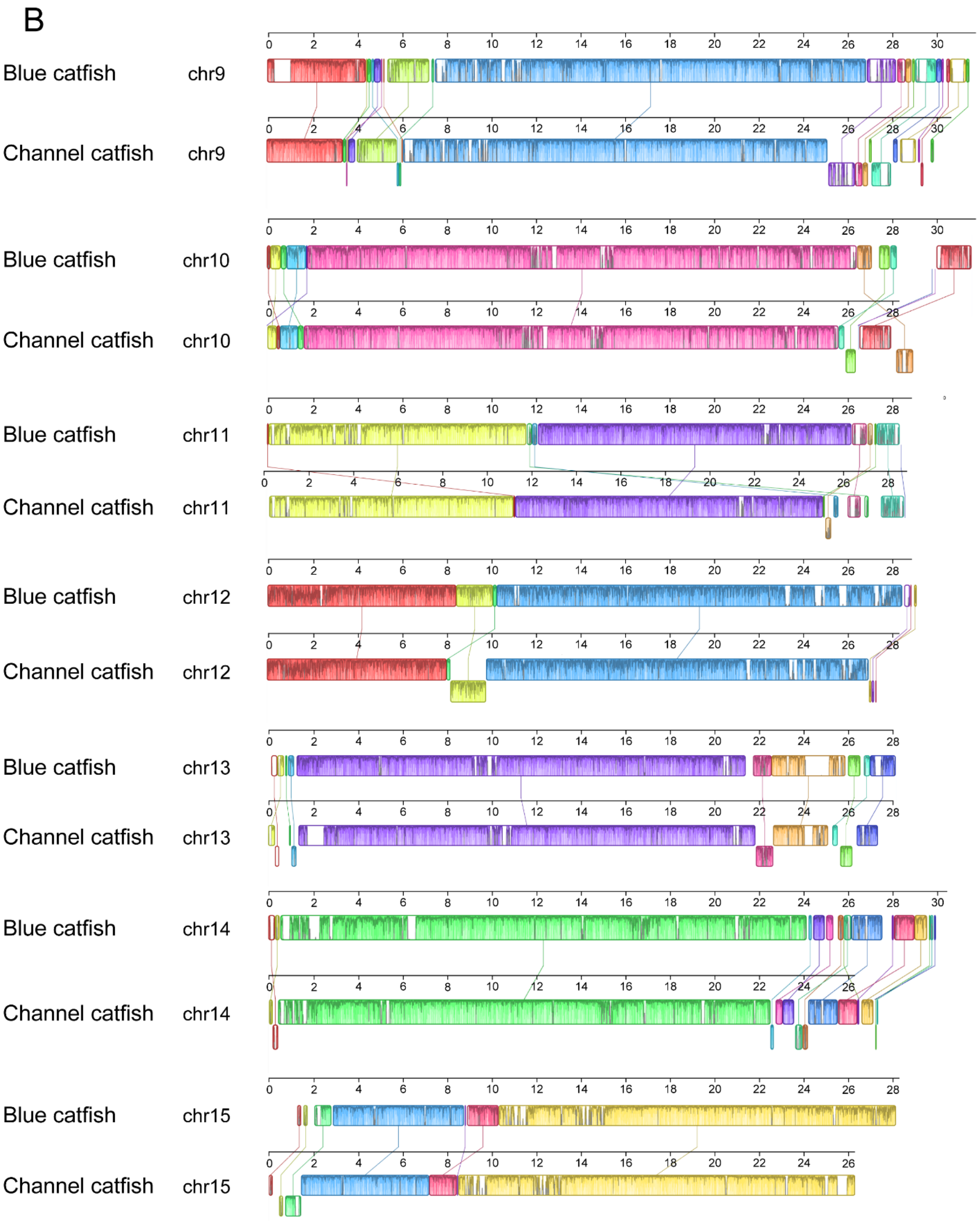

Figure S2 (continued). Synteny alignments of blue catfish and channel catfish chromosomes based on DNA sequence similarity.

(C). Synteny analysis of chromosome 16~22 between channel catfish and blue catfish (unit: Mbp).

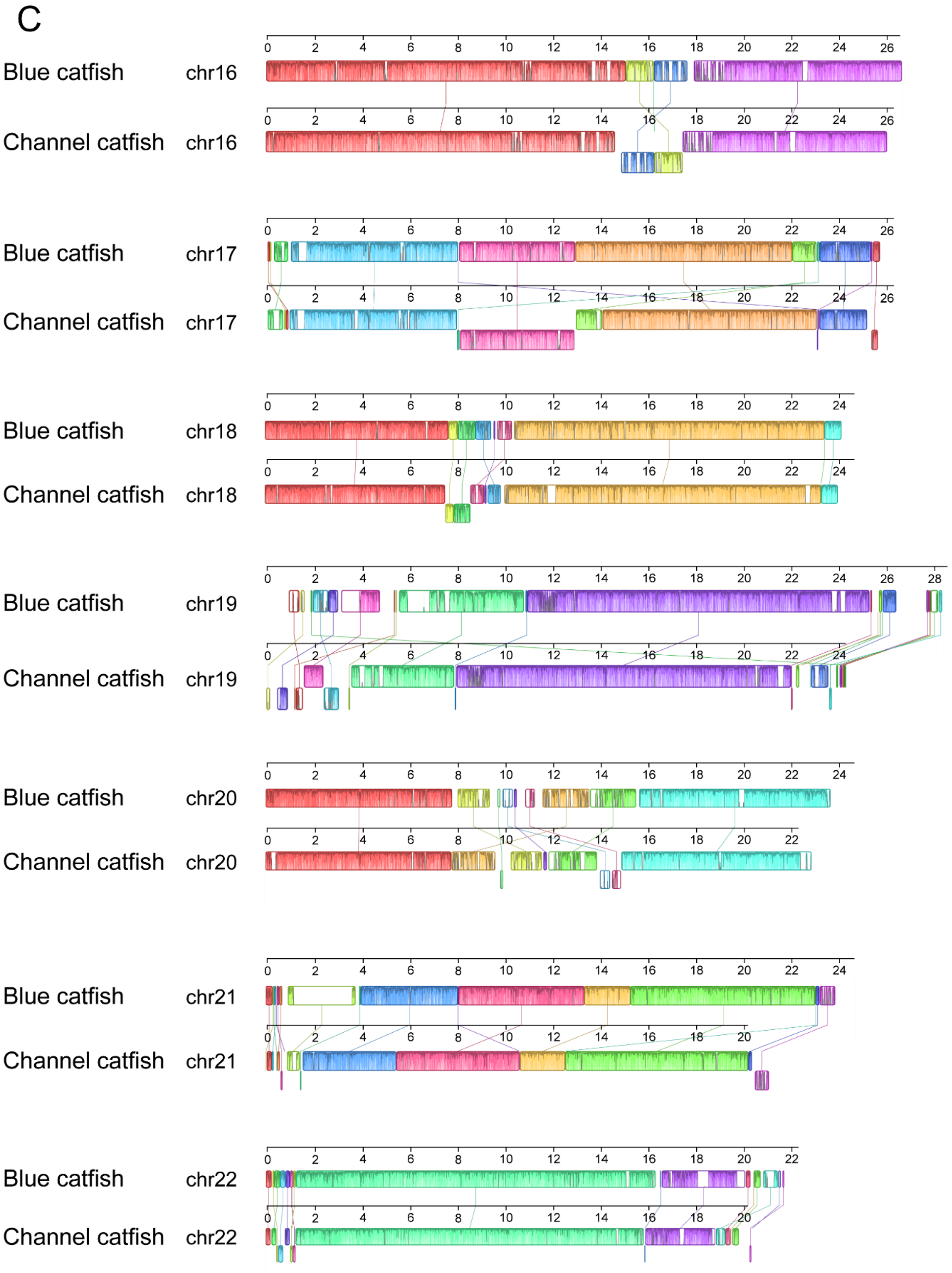

Figure S2 (continued). Synteny alignments of blue catfish and channel catfish chromosomes based on DNA sequence similarity.

(D). Synteny analysis of chromosome 23~29 between channel catfish and blue catfish (unit: Mbp).

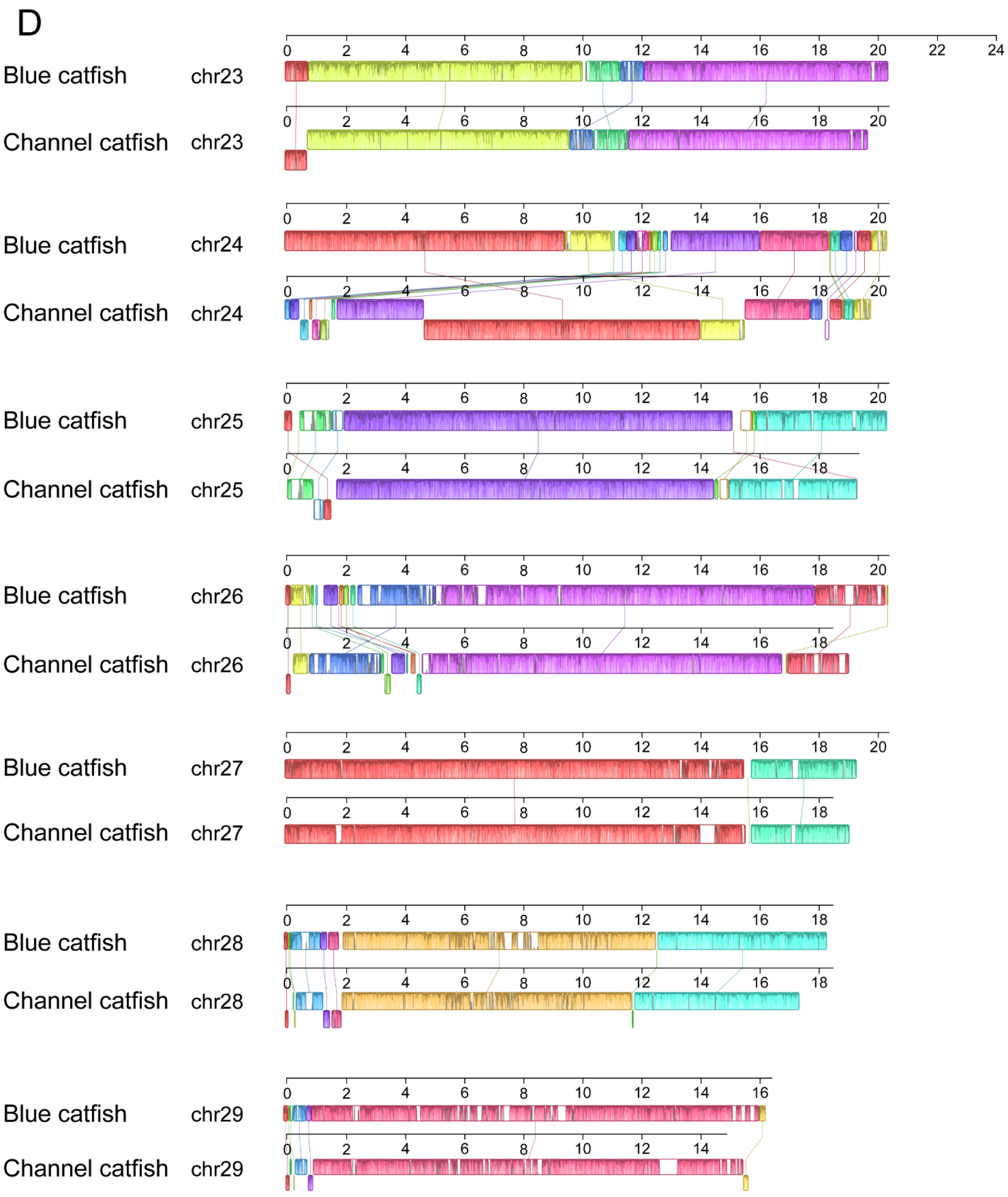

Table S1. Summary of Illumina (10× Genomics) sequencing data generated for blue catfish genome assembly.

	D&B_M	D&B_F	Rio_M	Rio_F
**Accession number**	SRR18966195	SRR18966196	SRR18966193	SRR18966194
**Strain**	D & B	D & B	Rio Grande	Rio Grande
**Sex**	Male	Female	Male	Female
**PacBio data**	No	Yes	No	No
**Illumina NovaSeq (10× Genomics)**	Yes	Yes	Yes	Yes
**Total Illumina sequence (Gb)**	16.55	32.85	18.67	21.87
**Illumina sequence depth**	13×	28.7×	14.2×	18.6×
**Raw reads**	110,359,012	219,030,872	124,453,646	145,777,450
**Filtered reads**	82,508,560	173,468,774	91,588,034	113,134,956
**Mapping rate (channel reference)**	76.30%	83.06%	73.45%	81.05%
**% genome mapped (channel)**	94.72%	96.76%	94.09%	95.88%
**Mapping rate (blue reference)**	85.17%	90.34%	83.97%	89.41%
**% genome mapped (blue)**	98.29%	99.46%	96.80%	98.19%

Table S2. Chromosomal locations of telomeric regions in the blue catfish genome.

Chromosome	Chromosome length (bp)	Telomeric region	TRM unit	TRM length (bp)
chr1	38,502,147	38,490,627-38,502,147	TTAGGG	11,521
chr2	37,347,697	37,328,958-37,347,697	TTAGGG	18,740
chr3	37,403,401	1-14,694	TTAGGG	14,694
chr3	37,403,401	37,388,652-37,403,401	TTAGGG	14,840
chr4	34,879,847	1-18,799	TTAGGG	18,799
chr4	34,879,847	34,876,224-34,879,847	TTAGGG	3,624
chr5	32,683,838	1-15,008	TTAGGG	15,008
chr5	32,683,838	32,672,972-32,683,838	TTAGGG	10,867
chr6	34,832,723	1-2,316	TTAGGG	2,316
chr6	34,832,723	34,817,154-34,832,723	TTAGGG	15,570
chr7	35,738,446	1-13,222	TTAGGG	13,222
chr7	35,738,446	35,733,978-35,738,446	TTAGGG	4,469
chr8	31,222,341	1-14,599	TTAGGG	14,599
chr8	31,222,341	31,213,178-31,222,341	TTAGGG	9,164
chr9	31,984,930	1-42	TTAGGG	17,035
chr9	31,984,930	31,972,799-31,984,930	TTAGGG	12,132
chr10	31,655,194	1-12,284	TTAGGG	12,284
chr12	29,997,346	29,982,864-29,997,346	TTAGGG	14,483
chr13	28,244,497	1-383	TTAGGG	383
chr13	28,244,497	28,228,599-28,244,497	TTAGGG	15,899
chr14	33,164,699	33,154,880-33,164,699	TTAGGG	9,820
chr15	27,933,702	27,917,339-27,933,702	TTAGGG	16,364
chr16	26,605,954	1-14,636	TTAGGG	14,636
chr17	25,991,458	1-7,157	TTAGGG	7,157
chr18	24,759,037	1-5,697	TTAGGG	5,697
chr18	24,759,037	24,743,022-24,759,037	TTAGGG	16,016
chr19	28,130,347	1-8,377	TTAGGG	8,377
chr19	28,130,347	28,117,110-28,130,347	TTAGGG	13,238
chr20	23,304,372	1-11,449	TTAGGG	11,449
chr20	23,304,372	23,291,168-23,304,372	TTAGGG	13,205
chr21	24,604,880	24,600,949-24,604,880	TTAGGG	3,932
chr22	21,156,995	1-15,567	TTAGGG	15,567
chr23	20,281,674	1-9,791	TTAGGG	9,791
chr24	20,306,282	1-6,740	TTAGGG	6,740
chr24	20,306,282	20,287,362-20,306,282	TTAGGG	18,921
chr25	20,550,178	20,530,827-20,550,178	TTAGGG	19,352
chr26	20,178,937	1-15,939	TTAGGG	15,939
chr26	20,178,937	20,171,519-20,178,937	TTAGGG	7,419
chr27	18,867,290	1-11,567	TTAGGG	11,567
chr27	18,867,290	18,852,505-18,867,290	TTAGGG	14,786
chr28	18,616,282	1-13,828	TTAGGG	13,828
chr29	15,876,424	1-11,389	TTAGGG	11,389
chr29	15,876,424	15,864,517-15,876,424	TTAGGG	11,908

TRM, telomeric repeat motif.

Table S3. Chromosomal locations of telomeric regions in the channel catfish genome.

Chromosome	Chromosome length (bp)	Telomeric region	TRM unit	TRM length (bp)
chr1	37,510,255	1-100	TTAGGG	100
chr1	37,510,255	37,509,904-37,510,255	TTAGGG	262
chr2	37,257,225	1-130	TTAGGG	130
chr4	34,595,840	1-225	TTAGGG	225
chr9	30,212,908	1-226	TTAGGG	226
chr9	30,212,908	30,212,557-30,212,908	TTAGGG	352
chr11	28,208,075	1-201	TTAGGG	201
chr16	25,964,114	25,963,788-25,964,114	TTAGGG	327
chr20	22,470,590	3-359	TTAGGG	357
chr20	22,470,590	22,470,389-22,470,575	TTAGGG	187

TRM, telomeric repeat motif.

Table S4. RNA sequencing data yield, quality control, and alignment statistics to channel catfish and blue catfish genomes.

Sample	Number of raw reads	Number of filtered reads	% of uniquely mapped reads to channel genome (IpCoco 1.2)	% of uniquely mapped reads to blue genome (this assembly)	% of uniquely mapped reads to blue pseudo genome*
Blue_catfish_liver_1_1	39,671,838	39,524,026	61.0%	83.2%	68.1%
Blue_catfish_liver_2_1	24,723,270	24,644,364	62.1%	85.4%	68.4%
Blue_catfish_liver_1_2	87,981,866	87,650,476	70.6%	87.5%	76.7%
Blue_catfish_liver_2_2	66,425,710	66,215,758	68.0%	84.4%	74.1%

* A pseudo blue catfish genome was constructed based on the channel reference genome IpCoco 1.2, by replacing the channel alleles with blue alleles at 15,685,661 channel-blue SNP positions.

NCBI GEO Accession numbers: SRR18989496 (Blue_catfish_liver_1_1); SRR18989495 (Blue_catfish_liver_2_1); SRR16609847 (Blue_catfish_liver_1_2); SRR16609846 (Blue_catfish_liver_2_2).

Table S5. Summary nucleotide substitutions in the mitochondrial genome between blue catfish D&B and Rio Grande strains.

Gene name	Type	# of substitutions	Synonymous	Non-synonymous	SNP positions
12S_rRNA	rRNA	1	-	-	378
16S_rRNA	rRNA	1	-	-	2527
tRNA-Cys	tRNA	1	-	-	5326
tRNA-Thr	tRNA	1	-	-	15489
ATP6	Coding	2	2	0	8151,8232
COX1	Coding	2	2	0	6168,6171
COX2	Coding	2	2	0	7509,7554
COX3	Coding	3	3	0	8903,9374,9413
CYTB	Coding	4	3	1	14452,14490,14830,15073
ND1	Coding	6	5	1	2928,3078,3443,3545,3644,3770
ND2	Coding	5	5	0	4114,4408,4478,4720,4756
ND3	Coding	2	2	0	9954,9966
ND4	Coding	3	2	1	10795,11231,11365
ND5	Coding	7	5	2	12385,12484,13159,13433,13436,13639,13720
D-loop	Intergenic	13	-	-	15698,15745,15750,15790,15840,15993,16054, 16065,16206,16207,16208,16284,16395

Table S6. Summary nucleotide substitutions in the mitochondrial genome between blue catfish and channel catfish.

Gene name	Type	# of substitutions	Synonymous	Non-synonymous
**RNA genes**				
12S_rRNA	rRNA	12	-	-
16S_rRNA	rRNA	49	-	-
tRNA	tRNA	48	-	-
**Protein-coding genes**				
ATP6	Coding	58	54	4
ATP8	Coding	9	8	1
COX1	Coding	131	129	2
COX2	Coding	43	39	4
COX3	Coding	53	50	3
CYTB	Coding	98	90	8
ND1	Coding	94	87	7
ND2	Coding	118	113	5
ND3	Coding	35	27	8
ND4	Coding	136	127	9
ND4L	Coding	26	24	2
ND5	Coding	197	173	24
ND6	Coding	43	41	2
Total (coding)		**1041**	**962**	**79**
**Intergenic regions**				
D-loop	Intergenic	95	-	-
Intergenic	Intergenic	3	-	-

Table S7. Summary of gene family expansion and contraction results.

Species	# of expanded gene families	# of contracted gene families	# of rapid expansion families	# of rapid contraction families
Blue catfish	331	1963	50	36
Large yellow croaker	1563	998	79	12
Nile tilapia	994	601	378	5
Zebrafish	2562	2296	175	8
Japanese puffer	331	2789	25	74
Greater amberjack	333	1160	59	22
Northern pike	682	2926	54	3
Channel catfish	408	506	101	6
Guppy	288	1796	23	31
Spotted gar	264	9241	7	0
Atlantic herring	809	3999	77	3

Table S8. List of gene families underwent rapid expansion in blue catfish.

Gene family ID	# of genes in blue catfish	# of genes in channel catfish	Description
GF_346	7	3	Immunoglobulin V-set domain / T cell receptor delta
GF_1067	5	1	Immunoglobulin
GF_1406	3	1	Immunoglobulin
GF_1741	11	0	Immunoglobulin C1set
GF_2055	7	0	Immunoglobulin
GF_15008	2	0	Immunoglobulin
GF_17665	4	0	Immunoglobulin like domain
GF_20405	3	0	Immunoglobulin
GF_21664	2	0	Immunoglobulin
GF_23	34	1	C-type lectin like
GF_4823	7	5	C-type lectin like
GF_20403	3	1	C-type lectin like
GF_913	3	1	Sushi/SCR/CCP domain
GF_16823	3	1	CD225/Dispanin family
GF_19387	2	0	Chemokine interleukin 8 like domain
GF_18525	2	1	Thrombomodulin like
GF_17672	2	0	Glucocorticoid receptor
GF_1024	3	2	Transcobalamin1 like
GF_1735	9	1	Reverse transcriptase domain
GF_4827	10	1	Reverse transcriptase domain
GF_19172	2	1	Reverse transcriptase domain
GF_20531	3	0	Transposase, Tc1 like
GF_88	27	2	Harbinger transposase derived nuclease
GF_17747	2	0	Reverse transcriptase, RNase H like domain
GF_19038	2	0	LINE1 type transposase domain containing protein 1
GF_4822	3	1	Essential MCU regulator, mitochondrial
GF_19438	2	0	Short transmembrane mitochondrial protein 1
GF_309	18	0	Kelch repeat type 1
GF_19460	3	0	Zinc finger C2H2 type
GF_20406	3	1	Zinc finger, RING type
GF_20550	3	1	Zinc finger, CCHC type
GF_17348	2	1	EF hand domain
GF_20549	2	0	EF hand, Ca insensitive
GF_12157	3	1	US22 like
GF_16886	2	1	uncharacterized protein LOC106590896
GF_20506	3	1	uncharacterized protein LOC108262470
GF_4829	2	1	uncharacterized protein
GF_482	7	1	uncharacterized protein LOC108274681
GF_721	18	0	uncharacterized protein LOC106511789
GF_1562	3	1	Centrosome and spindle pole associated protein 1
GF_1953	8	4	GPCR family 3, extracellular calcium-sensing receptor
GF_1954	3	1	GPCR family 3, extracellular calcium-sensing receptor
GF_4602	7	3	ATPase, AAA type, core
GF_11841	2	0	HMG domain containing protein 3, CxC4 like cysteine cluster
GF_14797	3	1	Beta thymosin
GF_15174	2	0	UDP glucuronosyl / UDP glucosyltransferase
GF_15670	2	0	parathyroid hormone
GF_16173	2	1	Glutathione S transferase, C terminal
GF_17368	2	0	Death associated protein kinase 2
GF_17671	2	1	Pleckstrin homology domain
