# Supplementary material for: Chromosome-level assembly and annotation of the blue catfish Ictalurus furcatus, an aquaculture species for hybrid catfish reproduction, epigenetics, and heterosis studies
Source: Gigascience. 2022 Jul 9;11:giac070. doi: 10.1093/gigascience/giac070 (PMC9270728; doi:10.1093/gigascience/giac070)
Supplement: giac070_Reviewer_1_Report_Original_Submission — Mikhail Ozerov -- 5/23/2022 Reviewed [file giac070_reviewer_1_report_original_submission.pdf]

Reviewer Report

Title: Chromosome-level assembly and annotation of the blue catfish Ictalurus furcatus, an aquaculture species for hybrid catfish reproduction, epigenetics, and heterosis studies

Version: Original Submission **Date:** 5/23/2022

Reviewer name: Mikhail Ozerov

Reviewer Comments to Author:

The manuscript of Wang et al. "Chromosome-level assembly and annotation of the blue catfish Ictalurus furcatus, an aquaculture species for hybrid catfish reproduction, epigenetics, and heterosis studies" describes the high quality assembly of the genome and provides valuable information on evolution and genomic variation of blue catfish. Overall, a very useful resource. However, before accepting the manuscript for publication, some minor issues should be addressed.
Minor revisions:
Material and Methods:
L.164. Placing description of PacBio library preparation and sequencing to a separate paragraph or even separate section (given that description of 10x sequencing is separated) would help readers.
L.181; L.228: "NovaSeq 6000"
L.188. It is not clear, were all four 10x assemblies combined with PacBio-based assembly using quickmerge or only the 10x assembly of the same female D&B sequenced with PacBio? It is not clear were these assemblies (PacBio, four 10x and combined PacBio+10x) further used or discarded. Please clarify.
L. 230: Please clarify the parameters used with Trimmomatic and BWA aligner software.
L.241: The authors mentioned "repeat masked assembly of blue catfish", however, the section describing repeat annotation occurs later (L.301-306). Consider to place "Repeat annotation" section before the description of SNP identification methods.
L.254-255: Similarly to the previous comment, repeat annotation description should appear before "Gene prediction and functional annotation" part.
Results:
L. 361-362; 410-411; 416-417; 454-457; 494-699; 505-507: This reads as methods, consider to place in the proper sections of Material and Methods or reword.
L. 389: Not clear, should this read as "A total of 55 complete clusters of 18S-5.8S-28S rRNA genes were predicted"? However, there are 55 of 28S, 55 of 18S and 57 of 5.8S in the Table 2.
L.434-435: It would be useful to indicate in which gene the non-synonymous mutation was observed (ND5?).
L. 442: space missing, "….estimated to be 13 to 15 SNPs…"
L.470-472. This should be placed in the Methods along with the rational of pseudogenome generation.
Discussion:
L. 520-523; L. 569-571: These parts in the Discussion read as introduction, referring to the lack of the blue catfish genome assembly, which sounds confusing. I would recommend to reword or remove these

sentences.

Methods

Are the methods appropriate to the aims of the study, are they well described, and are necessary controls included? Choose an item.

Conclusions

Are the conclusions adequately supported by the data shown? Choose an item.

Reporting Standards

Does the manuscript adhere to the journal's guidelines on minimum standards of reporting? Choose an item.

Choose an item.

Statistics

Are you able to assess all statistics in the manuscript, including the appropriateness of statistical tests used? Choose an item.

Quality of Written English

Please indicate the quality of language in the manuscript: Choose an item.

Declaration of Competing Interests

Please complete a declaration of competing interests, considering the following questions:

- Have you in the past five years received reimbursements, fees, funding, or salary from an organisation that may in any way gain or lose financially from the publication of this manuscript, either now or in the future?
- Do you hold any stocks or shares in an organisation that may in any way gain or lose financially from the publication of this manuscript, either now or in the future?
- Do you hold or are you currently applying for any patents relating to the content of the manuscript?
- Have you received reimbursements, fees, funding, or salary from an organization that holds or has applied for patents relating to the content of the manuscript?
- Do you have any other financial competing interests?
- Do you have any non-financial competing interests in relation to this paper?

If you can answer no to all of the above, write 'I declare that I have no competing interests' below. If your reply is yes to any, please give details below.

I declare that I have no competing interests

I agree to the open peer review policy of the journal. I understand that my name will be included on my report to the authors and, if the manuscript is accepted for publication, my named report including any attachments I upload will be posted on the website along with the authors' responses. I agree for my report to be made available under an Open Access Creative Commons CC-BY license (http://creativecommons.org/licenses/by/4.0/). I understand that any comments which I do not wish to be included in my named report can be included as confidential comments to the editors, which will not be published.

Choose an item.

To further support our reviewers, we have joined with Publons, where you can gain additional credit to further highlight your hard work (see: https://publons.com/journal/530/gigascience). On publication of this paper, your review will be automatically added to Publons, you can then choose whether or not to claim your Publons credit. I understand this statement.

Yes Choose an item.
