# Supplementary material for: Chromosome-level assembly and annotation of the blue catfish Ictalurus furcatus, an aquaculture species for hybrid catfish reproduction, epigenetics, and heterosis studies
Source: Gigascience. 2022 Jul 9;11:giac070. doi: 10.1093/gigascience/giac070 (PMC9270728; doi:10.1093/gigascience/giac070)
Supplement: giac070_Reviewer_2_Report_Original_Submission — Jie Mei -- 5/23/2022 Reviewed [file giac070_reviewer_2_report_original_submission.pdf]

Reviewer Report

Title: Chromosome-level assembly and annotation of the blue catfish Ictalurus furcatus, an aquaculture species for hybrid catfish reproduction, epigenetics, and heterosis studies

Version: Original Submission **Date:** 5/23/2022

Reviewer name: Jie Mei

Reviewer Comments to Author:

Wang et al. generated a chromosome-scale genome sequence assembly of blue catfish based on PacBio HiFi reads. This 842 Mbp genome assembly is distributed in 29 chromosomes, corresponding to a high degree of BUSCO completeness. The authors harnessed the highly contiguous assembly for analyses of synteny against channel catfish and within-species SNP density between D&B and Rio Grande. This is a valuable resource for comparison against various Siluriformes species. This assembly will certainly improve the genetic potential for commercial production of channel catfish â™€ ï‚´ blue catfish â™‚.
However, I have some comments that should be addressed:
1. It seems that the estimated genome size, heterozygosity rate and repeat ratio were not present in the manuscript. The authors should provide estimated genome feature results based on multiple k-mer sizes (e.g. 17-25) using short reads from the Illumina platform.
2. Line 201-203. Hifiasm is a haplotype-aware assembler, whose result is already highly accurate. Pilon is not required because short read mapping is more likely to be wrong and it would introduce errors by wrong alignment especially in repetitive regions.
3. Line 415-424. Since anchoring short contigs to chromosomes using low density genetics linkage maps (compared to channel catfish) is prone to inversions and translocations, rearrangements should exclude unrealiable sequences (e.g. short contigs with low number of markers).
4. Line 475-477. The RNA-Seq mapping rates seems still low for such a complete assembly. The authors should try some aligners which are aware of splicing (e.g. STAR and Hisat2) instead of bwa.
Minor corrections:
Figures 5 and 6 show duplicate information, and the two figures should be combined into one.
Line 464: 5 SNP > 5 SNPs
Line 684: 2.6 SNP > 2.6 SNPs

Methods

Are the methods appropriate to the aims of the study, are they well described, and are necessary controls included? Choose an item.

Conclusions

Are the conclusions adequately supported by the data shown? Choose an item.

Reporting Standards

Does the manuscript adhere to the journal's guidelines on minimum standards of reporting? Choose an item.

Choose an item.

Statistics

Are you able to assess all statistics in the manuscript, including the appropriateness of statistical tests used? Choose an item.

Quality of Written English

Please indicate the quality of language in the manuscript: Choose an item.

Declaration of Competing Interests

Please complete a declaration of competing interests, considering the following questions:

- Have you in the past five years received reimbursements, fees, funding, or salary from an organisation that may in any way gain or lose financially from the publication of this manuscript, either now or in the future?
- Do you hold any stocks or shares in an organisation that may in any way gain or lose financially from the publication of this manuscript, either now or in the future?
- Do you hold or are you currently applying for any patents relating to the content of the manuscript?
- Have you received reimbursements, fees, funding, or salary from an organization that holds or has applied for patents relating to the content of the manuscript?
- Do you have any other financial competing interests?
- Do you have any non-financial competing interests in relation to this paper?

If you can answer no to all of the above, write 'I declare that I have no competing interests' below. If your reply is yes to any, please give details below.

I declare that I have no competing interests

I agree to the open peer review policy of the journal. I understand that my name will be included on my report to the authors and, if the manuscript is accepted for publication, my named report including any attachments I upload will be posted on the website along with the authors' responses. I agree for my report to be made available under an Open Access Creative Commons CC-BY license (http://creativecommons.org/licenses/by/4.0/). I understand that any comments which I do not wish to be included in my named report can be included as confidential comments to the editors, which will not be published.

Choose an item.

To further support our reviewers, we have joined with Publons, where you can gain additional credit to further highlight your hard work (see: https://publons.com/journal/530/gigascience). On publication of this paper, your review will be automatically added to Publons, you can then choose whether or not to claim your Publons credit. I understand this statement.

Yes Choose an item.
