# Supplementary material for: Chromosome-level assembly and annotation of the blue catfish Ictalurus furcatus, an aquaculture species for hybrid catfish reproduction, epigenetics, and heterosis studies
Source: Gigascience. 2022 Jul 9;11:giac070. doi: 10.1093/gigascience/giac070 (PMC9270728; doi:10.1093/gigascience/giac070)
Supplement: giac070_Supplemental_Files [file Blue_catfish_genome_point-by-point_response_to_reviews_v1.pdf › Blue_catfish_genome_point-by-point_response_to_reviews_v1.pdf]

Manuscript Number: GIGA-D-22-00096

Reviewer #1

The manuscript of Wang et al. "Chromosome-level assembly and annotation of the blue catfish Ictalurus furcatus, an aquaculture species for hybrid catfish reproduction, epigenetics, and heterosis studies" describes the high quality assembly of the genome and provides valuable information on evolution and genomic variation of blue catfish. Overall, a very useful resource. However, before accepting the manuscript for publication, some minor issues should be addressed.

We thank Reviewer #1 for his positive comments, and we really appreciate his advice and suggestions to improve our manuscript. We revised our manuscript based on the reviewer's suggestions, and we are confident that the current form is suitable for publication at *GigaScience*.

Minor revisions:
Material and Methods:
L.164. Placing description of PacBio library preparation and sequencing to a separate paragraph or even separate section (given that description of 10x sequencing is separated) would help readers.

We agree with the reviewer. The PacBio library prep and sequencing method is now in a separate paragraph as the reviewer suggested.

L.181; L.228: "NovaSeq 6000"

These typos were corrected. Thank you for catching this.

L.188. It is not clear, were all four 10x assemblies combined with PacBio-based assembly using quickmerge or only the 10x assembly of the same female D&B sequenced with PacBio? It is not clear were these assemblies (PacBio, four 10x and combined PacBio+10x) further used or discarded. Please clarify.

Only the 10x assembly of the same female D&B sequenced with PacBio were attempted to merge with the PacBio assembly. However, no improvement was achieved, and the final PacBio+10x assembly is identical to PacBio assembly, suggesting that the PacBio is already in high contiuity. We now made this clear in the main text.

L. 230: Please clarify the parameters used with Trimmomatic and BWA aligner software.

These parameters have been added to the Method section.

L.241: The authors mentioned "repeat masked assembly of blue catfish", however, the section describing repeat annotation occurs later (L.301-306). Consider to place "Repeat annotation" section before the description of SNP identification methods.

We agree with the reviewer. The repeat annotation description subsection has been reordered as the reviewer suggested.

L.254-255: Similarly to the previous comment, repeat annotation description should appear before "Gene prediction and functional annotation" part.

We agree with the reviewer. The repeat annotation description subsection has been reordered as the reviewer suggested.

Results:
L. 361-362; 410-411; 416-417; 454-457; 494-699; 505-507: This reads as methods, consider to place in the proper sections of Material and Methods or reword.

We agree with the reviewer. These method-related sentences were either moved to the corresponding Method section, or deleted due to redundancy.

L. 389: Not clear, should this read as "A total of 55 complete clusters of 18S-5.8S-28S rRNA genes were predicted"? However, there are 55 of 28S, 55 of 18S and 57 of 5.8S in the Table 2.

The reviewer is right, and we corrected this typo. There are two extra copies of 5.8S rRNA, which may reflect incompletely assembled rDNA clusters.

L.434-435: It would be useful to indicate in which gene the non-synonymous mutation was observed (ND5?).

We agree. The genes with non-synonymous mutations were listed in Table S5.

L. 442: space missing, "….estimated to be 13 to 15 SNPs…"

We inserted the space. Thank you for catching this.

L.470-472. This should be placed in the Methods along with the rational of pseudogenome generation.

We have revised these sentences. We do think the pseudogenome mapping stats are part of the results, and it justifies the needs of a *de novo* blue catfish assembly.

Discussion:
L. 520-523; L. 569-571: These parts in the Discussion read as introduction, referring to the lack of the blue catfish genome assembly, which sounds confusing. I would recommend to reword or remove these sentences.

This is a great suggestion. We deleted these sentence as the reviewer suggested.

Reviewer #2
Wang et al. generated a chromosome-scale genome sequence assembly of blue catfish based on PacBio HiFi reads. This 842 Mbp genome assembly is distributed in 29 chromosomes, corresponding to a high degree of BUSCO completeness. The authors harnessed the highly contiguous assembly for analyses of synteny against channel catfish and within-species SNP density between D&B and Rio Grande. This is a valuable resource for comparison against various Siluriformes species. This assembly will certainly improve the genetic potential for commercial production of channel catfish ♀ ▯ blue catfish ♂.

We thank Reviewer #2 for his positive comments of our manuscript, and his suggestions to enhance our manuscript. We carefully addressed the specific comments and concerns below by additional analysis and manuscript revision.

However, I have some comments that should be addressed:

1. It seems that the estimated genome size, heterozygosity rate and repeat ratio were not present in the manuscript. The authors should provide estimated genome feature results based on multiple k-mer sizes (e.g. 17-25) using short reads from the Illumina platform.

We have performed genome size estimation to estimate the PacBio genome coverge before we did the PacBio sequencing. We thank Reviewer #2 for pointing this out. The estimated genome size and heterozygosity rate are now added to the maintext, as well as the corresponding methods.

2. Line 201-203. Hifiasm is a haplotype-aware assembler, whose result is already highly accurate. Pilon is not required because short read mapping is more likely to be wrong and it would introduce errors by wrong alignment especially in repetitive regions.

We agree with the reviewer that Hifisam already produced accurate assembly results, and this was reassured in our pilon results: only a few corrections have been made, and they are unlikely to affect the final assembly. To confirm that pilon correction did not introduce erroneous SNPs/structural variation, we mapped the PacBio reads to the genome assembly using minimap2, and no SNPs were found, indicating the assembly is accurate. In our next assembly, we will skip the pilon step as the reviewer suggested because it is not necessary.

3. Line 415-424. Since anchoring short contigs to chromosomes using low density genetics linkage maps (compared to channel catfish) is prone to inversions and translocations, rearrangements should exclude unrealiable sequences (e.g. short contigs with low number of markers).

We agree with the reviewer's point that insufficient amount of linkage markers can cause misorientiation of the scaffold assembly into chromosomes. Short contigs may cause issues. We are aware of this and we have addressed using the following strategies: 1) all of our assembled contigs are quite long (overall contig N50 = 8.6 Mb), and short contigs with ambiguous information were placed into unassembled scaffolds (chrUn); 2) on average, the number of markers per chr in blue catfish is 35. In contrast, we only have 11 scaffolds per chromosome,

which is much fewer than the number of markers. Therefore, the linkage marker density is sufficient to support this version of the chromosome assembly.

4. Line 475-477. The RNA-Seq mapping rates seems still low for such a complete assembly. The authors should try some aligners which are aware of splicing (e.g. STAR and Hisat2) instead of bwa.

We agree with the reviewer. We did use a splice-aware aligner (TopHat2), and the 85% RNA alignment rate is not super high because the total RNA sample were isolated a long-time ago. We recently generated additional RNA-seq data for a different project, and the mapping percentage is over 90%. In this manuscript, we just want to drive the point home that the alignment % improved from 60% to over 80% (Table S4).

Minor corrections:
Figures 5 and 6 show duplicate information, and the two figures should be combined into one.

We agree with the reviewer. The original Figure 5 and Figure 6 were combined to form the current Figure 5. The figure legends and citations in the maintext were modified accordingly.

Line 464: 5 SNP > 5 SNPs
We added the “s”. Thank you for catching this.

Line 684: 2.6 SNP > 2.6 SNPs
We added the “s”. Thank you for catching this.
